# Maize nodal root growth maintenance during water deficit: metabolic acclimation and the role of increased solute deposition in osmotic adjustment

**DOI:** 10.3389/fpls.2025.1566453

**Published:** 2025-06-09

**Authors:** Tyler J. McCubbin, Laura A. Greeley, Rachel A. Mertz, Sidharth Sen, Amelia E. Griffith, Shannon K. King-Miller, Kara Riggs, Nicole D. Niehues, Akanksha Pareek, Victoria J. Bryan, Shuai Zeng, Cheyenne Becker, Abdul Ghani, Trupti Joshi, Scott C. Peck, Melvin J. Oliver, Felix B. Fritschi, David M. Braun, Robert E. Sharp

**Affiliations:** ^1^ Division of Plant Science and Technology, University of Missouri, Columbia, MO, United States; ^2^ Plant Genetics Research Unit, USDA-ARS, Columbia, MO, United States; ^3^ Interdisciplinary Plant Group, University of Missouri, Columbia, MO, United States; ^4^ Department of Biochemistry, University of Missouri, Columbia, MO, United States; ^5^ Division of Biological Sciences, University of Missouri, Columbia, MO, United States; ^6^ MU Institute for Data Science and Informatics, University of Missouri, Columbia, MO, United States; ^7^ Department of Computer Science and Electrical Engineering, University of Missouri, Columbia, MO, United States; ^8^ Department of Biomedical Informatics, Biostatistics and Medical Epidemiology, University of Missouri, Columbia, MO, United States; ^9^ Christopher S. Bond Life Sciences Center, University of Missouri, Columbia, MO, United States

**Keywords:** kinematics, maize, metabolomics, nodal roots, root growth, transcriptomics, water deficit

## Abstract

Maize (*Zea mays* L.) nodal roots are characterized by their ability to maintain elongation under water deficit conditions that inhibit the growth of other organs. Physiological and molecular mechanisms underlying this response were investigated using a divided-container root growth system to impose uniform and steady water deficit (WD) conditions around the nodal roots of maize cv. FR697. Kinematic growth analysis demonstrated that continued nodal root elongation under water deficit involves maintenance of both growth zone length and rates of cell production from the meristem. Nodal roots that maintain growth during WD exhibit increased rates of net solute deposition throughout the growth zone that enable osmotic adjustment and continued tissue expansion. These abilities differ from the maize primary root, which exhibits impairment of both cell expansion and cell production when grown under similar water deficit conditions. Integration of transcriptomic and metabolomic profiling revealed molecular signatures of nodal root growth maintenance, including central transcriptional responses and metabolic pathways related to osmolyte accumulation, hormone signaling, and ROS homeostasis. Several metabolic responses differed from previous characterization of the primary root, including taurine accumulation and proline synthesis via the saccharopine pathway. Further, our analysis showed that metabolic acclimation rather than transcriptional control dominated the water deficit response of the nodal root growth zone. The study highlights novel insights into the interplay of morphogenic and metabolic responses that regulate the remarkable ability of nodal roots to maintain elongation under water deficit conditions.

## 1 Introduction

Drought is the primary limitation on agricultural yield worldwide (Kang et al., 2009). Since the uptake and acquisition of water is mediated by plant roots, understanding the mechanisms that govern root growth responses to water deficit (WD) are central to efforts to improve crop productivity (Voothuluru et al., 2024). Indeed, changes in root system architecture and water capture were likely a major contributing factor to the yield increases in the US corn (maize) belt over the last century (Hammer et al., 2009). With drought-related yield reductions for major crops projected to double by 2050, it is critical that the ability of plants to find and use the available soil water is improved (Li et al., 2009).

Maize is the most extensively cultivated cereal crop in the world, and is estimated to provide 20% of calories globally (Erenstein et al., 2022). Unlike the root system of a eudicot plant, in which a primary root (taproot) grows deep into the soil to find water, maize employs a fibrous root system, with two root types; the embryonic roots (primary and seminal) which supply water during seedling establishment, and stem-borne nodal roots (or “crown roots”) that provide most of the water after seedling establishment (McCully and Canny, 1988; Ahmed et al., 2018). The first nodal roots emerge from the coleoptilar node around 10–14 days after germination, when seedlings are around the V1-V2 stage, and are followed by nodal roots emerging from several consecutive nodes (Hochholdinger et al., 2004; Nielsen, 2013). Since they emerge from the base of the stem, nodal roots must grow through the upper strata of soil, which may include extremely dry top soil, before reaching water at depth (Ritchie, 1981; Sharp and Davies, 1985; Lynch, 2013). As reflected by measurements of tissue water potential (Ψ_W_), nodal roots can be the most water-stressed organ of a maize plant under soil drying conditions (Westgate and Boyer, 1985). Nodal roots are unique among plant organs for their capacity to maintain growth at Ψ_W_ sufficiently low to prevent growth of other plant tissues (Westgate and Boyer, 1985). This capacity is not limitless; when intense periods of drought result in excessively dry top soil, young nodal root tips may exhibit arrested growth before reaching the wetter soil lower in the profile (Sebastian et al., 2016). This response can result in compensatory development of the seedling root system but also can increase the vulnerability of the plant to lodging (Nielsen, 2022).

Root systems are known to exhibit high plasticity of growth and architecture in response to abiotic stresses (Kano et al., 2011; Fry et al., 2018), a response which underpins maintenance of yield in cereal crops (Ehdaie et al., 2012; Cai et al., 2017; Suralta et al., 2018). Increasing the root:shoot biomass ratio is a response to WD stress that has been described for more than a century (Opitz, 1904; Bünger, 1906; Sharp and Davies, 1989). To explore more soil area for retrievable water, a WD-stressed plant partitions a greater fraction of assimilated carbon to its root system; a reallocation of resources that is usually accompanied by inhibited shoot growth and leaf expansion (Nicolas et al., 1985; Van Volkenburgh and Boyer, 1985; Creelman et al., 1990; Pelleschi et al., 1997; Xu et al., 2015; Durand et al., 2016; Chen et al., 2022). The inhibition of shoot growth and maintenance of root growth involves the interplay of phytohormones including abscisic acid (ABA) and ethylene (Watts et al., 1981; Creelman et al., 1990; Saab et al., 1990; Sharp, 2002; Valluru et al., 2016).

The early nodal roots of the maize root system (first three emerging whorls) have been observed to grow the longest, and rooting depth is determined by more than simply the duration of root growth (Araki et al., 2000). When growing in a low Ψ_W_ environment, such as the dry topsoil, continued root growth requires osmotic adjustment via a decrease in the osmotic potential (Ψ_S_) of expanding cells so as to maintain turgor and thus expansive growth. This phenomenon has been demonstrated for both the maize primary and nodal root growth zones (Sharp and Davies, 1979; Westgate and Boyer, 1985; Sharp et al., 1990). While deeper nodal root growth is a key adaptation to retrieve water in dry soil (Gao and Lynch, 2016), and multiple genes controlling nodal root initiation have been identified (Jenkins, 1930; Hetz et al., 1996; Hochholdinger and Feix, 1998), relatively little is known about the underlying genetic and molecular mechanisms that maintain nodal root growth during WD.

The dynamic nature of soil drying presents a challenge to investigations of root physiology and drought responses. Variable root lengths can result in longer roots reaching wetter soil thus avoiding WD stress. Container size and plant water use efficiency both influence soil drying, confounding comparisons between experiments that differ in environmental conditions, pot size, and genotypes. To solve these challenges, a growth system was developed for this study in which the nodal roots are separated from the embryonic roots, allowing the precise manipulation of water available to the nodal roots, independent of the primary and seminal roots. In doing so, it was possible to supply a WD sufficient to deliver a mild reduction in leaf Ψ_W_ while imposing a substantial WD stress to the nodal roots, similar to field conditions, but precisely controlled. Although divided-container root growth systems are not new (Volkmar, 1997; Rostamza et al., 2013), the system developed for this study produced precise, repeatable, and carefully calibrated soil Ψ_W_ for the study of nodal root growth during WD stress. The system was also capable of imposing a steady state WD around the growing nodal roots, avoiding the need for a dynamic dry-down that often complicates studies of stress responses.

This paper presents the first of a series of studies in which the divided-root system approach was utilized to investigate the molecular mechanisms that underpin nodal root growth maintenance under low soil Ψ_W_ conditions. A multi-omics approach, profiling the transcriptional and metabolomic responses throughout the growing region of nodal roots, was employed, in combination with kinematic analysis of the spatial pattern of tissue expansion rates. The results revealed differentially accumulated transcripts (DATs) and metabolites (DAMs), some of which are shared in all regions and some that are distinct to different regions within the growth zone. Moreover, the limited concordance between the WD-responsive transcriptome and metabolome suggests that the stress responses are governed at both the transcriptomic and metabolic levels. The study provides a model in which nodal roots seek to maintain growth homeostasis in their response to the environment and in which growth maintenance is achieved by a multifaceted combination of transcriptomic and metabolomic controls to coordinate continued growth at low Ψ_W_.

## 2 Materials and methods

### 2.1 Design of the divided-container root growth system and soil preparation

**Figure 1:**
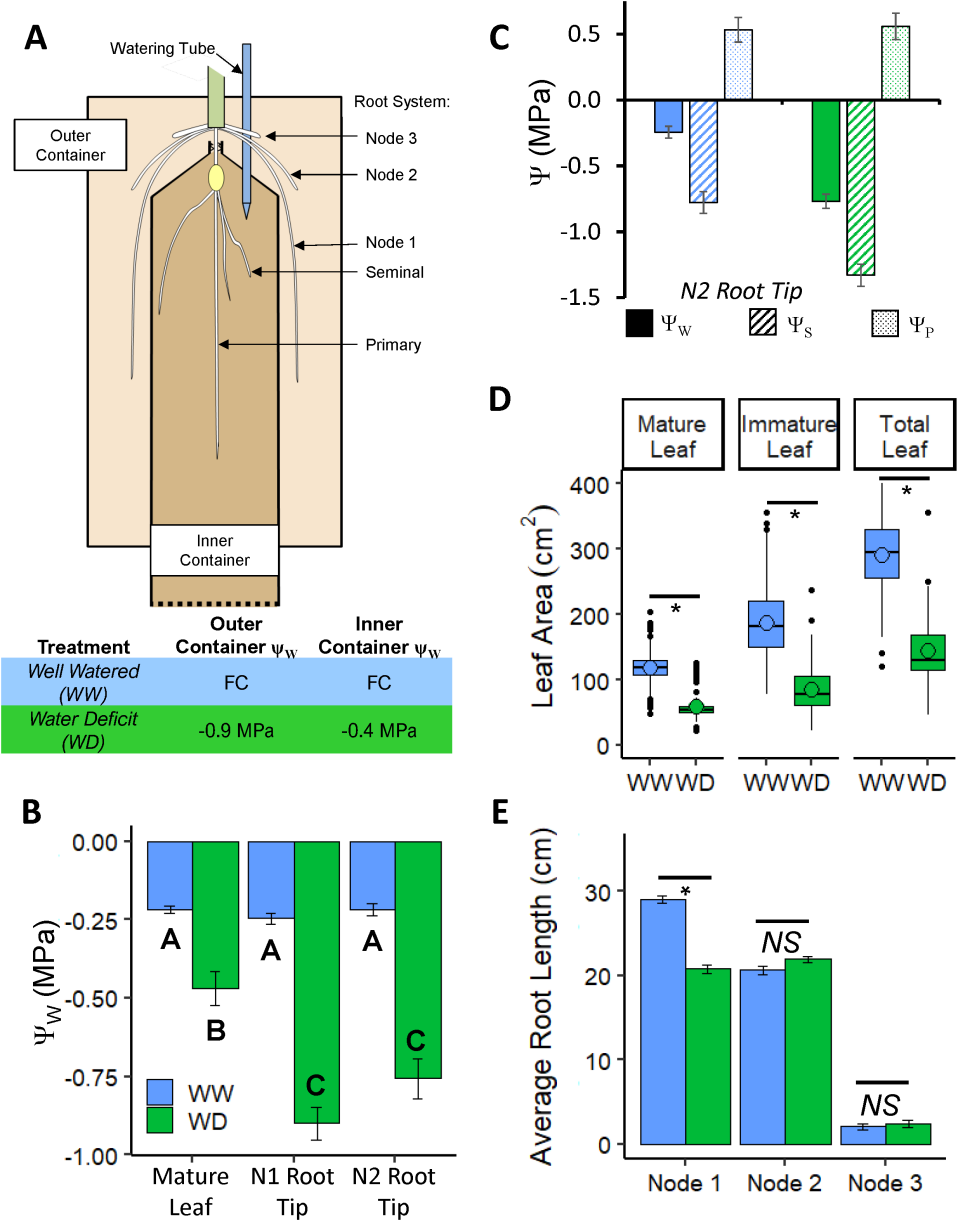
**(A)** Design of the divided-container root growth system. A germinated seedling was transplanted into the inner container before the emergence of the first whorl of nodal roots, which emerged into the soil in the outer container. Plants were then grown for 17 days before harvest. In the WW treatment, both containers contained soil at FC; transpired water was replenished every other day. In the WD treatment, the outer and inner containers were filled with soil at Ψ_W_ of -0.9 MPa and -0.4 MPa, respectively. **(B)** Tissue Ψ_W_ of mature leaf blade, N1 root tip, and N2 root tip at harvest. **(C)** N2 root tip Ψ_W_, Ψ_S_, and Ψ_P_. **(D)** Mature, immature and total leaf area per plant; mean values are denoted with open circles. **(E)** Average nodal root axial length per plant of nodes 1 - 3. Data in **(B, C, E)** are means ± SE; n = 21 combined from three experiments **(B, C)**, 146-169 plants from 16 experiments **(D, E)**. Statistical significance for **(B)** computed by two-way ANOVA with tissue and treatment as factors; both tissue, treatment, and their interaction term were determined to be significant factors (*P*< 0.03x10^-3^). Letters denote significant differences for treatment: tissue interaction by Tukey-Kramer multiple comparison test (*P*< 0.001). Statistical significance for **(D, E)** was calculated by Student’s *t-*Test, * *P* ≤ 0.001; *NS*, not significant.

The divided-container root growth system (**Figure 1A**; **Supplementary Figures S1A, B**) was constructed using a PVC tube to form an inner container measuring 1 m in length by 5 cm in diameter (0.001963 m^3^), and a consecutive series of PVC tube sections to form an outer container measuring 71 cm in length by 10 cm in diameter (0.004182 m^3^). Soil (PRO-MIX HP [high porosity]; Premier Tech, Québec, Canada) was sieved (4 mm screen size) for uniformity, and nutrition was provided as described previously (Dowd et al., 2019). The soil was dried in a forced-air oven at 55°C for a minimum of 50 h. For soil Ψ_W_ adjustments, soil and reverse-osmosis water were thoroughly mixed in plastic bags and allowed to equilibrate overnight. Water was added at 5 L per 4.5 kg soil to achieve a Ψ_W_ of -0.4 MPa, and 3 L per 4.5 kg soil to achieve a Ψ_W_ of -0.9 MPa. Sub-samples from each batch of -0.4 MPa and -0.9 MPa soil were measured in triplicate using an isopiestic thermocouple psychrometer to verify Ψ_W_ prior to using the soil in an experiment (Boyer and Knipling, 1965). If necessary, small additions of dry soil or water, followed by thorough mixing, were provided to adjust the Ψ_W_ as required. To achieve field capacity (FC, i.e. maximal water holding capacity) for the well-watered (WW) treatment, water was added to excess and allowed to drain for approximately 16 h.

### 2.2 Plant material and growth conditions

Maize (cv FR697) plants were grown in controlled environment growth chambers (model PGW40, Conviron, Winnipeg, Canada; conviron.com). The photoperiod was 16 h at a photon flux density of 750 µmol m^-2^ s^-1^ PAR. The air temperature was 27°C during the light period and 29°C during the dark period to maintain approximately constant soil temperature throughout the diurnal cycle (Riggs, 2016), and the relative humidity (RH) was constant at 80%. Seeds were surface sterilized and germinated as described previously (Dowd et al., 2019). After germination, seedlings were selected for uniformity (primary roots of 1–3 cm in length) and transplanted into the inner container with a “plug” of FC soil contained in a 5 x 4 cm plastic cone with a small hole such that the coleoptile had just emerged from the soil and the seedling roots were contained in the inner container; germination was defined as day 1, and transplanting was performed on day 5. A 21.7 cm x 9.2 cm plastic sheet was wrapped around the top of the inner container and filled with 4–5 cm of FC soil for the coleoptile to emerge into over the next 48 hours. All seedling handling and transplanting steps were conducted at near-saturation RH and using a dim green “safe” light (Saab et al., 1990). After 48 h (prior to the initiation of nodal roots), the outer container was assembled over the inner container. In the WD treatment, the outer and inner containers were filled with soil at Ψ_W_ of -0.9 MPa and -0.4 MPa, respectively. In the WW treatment, both containers were filled with soil at FC. Molding putty was used to prevent nodal roots from growing into the inner container. Soil was filled to the top of the outer container, entirely covering the inner container but allowing the seedling to protrude. The assembly was wrapped in insulating foam to reflect light and stabilize daytime soil temperatures. To maintain the soil water status during the WW treatment, plant/container assemblies were weighed every other day and transpired water was replenished. Water was added to the inner container using a plastic tube emanating from the central area of the container and protruding upwards through the soil; when appropriate, a syringe was used to add exactly the amount of water equating to the mass loss since the previous period of watering. Our method of replenishing water slightly underestimated the amount of water transpired due to the progressive fresh weight gain of the shoot. Also, we did not replenish water transpired from the outer container via the nodal root system. However, preliminary experiments showed that the maximum decreases in soil water potential over the course of the experiments were approximately -0.10 MPa and -0.04 MPa in the inner and outer containers, respectively (**Supplementary Figures S1C, D**). In preliminary experiments with plants growing with FC soil in both containers, the effect of aeration was assessed by inserting two perforated pipettes through the length of the inner container. The pipettes were connected by tubing to a fish tank aerator that provided 10 L·min^-1^ of air which was humidified (to minimize soil drying) by bubbling through deionized water. Inner container aeration had no effect on primary root (inner container) or nodal root (outer container) growth, or on leaf area development (**Supplementary Figures S1F, G**). Accordingly, supplementary aeration was not provided in subsequent experiments.

### 2.3 Physiological measurements

**Figure 2:**
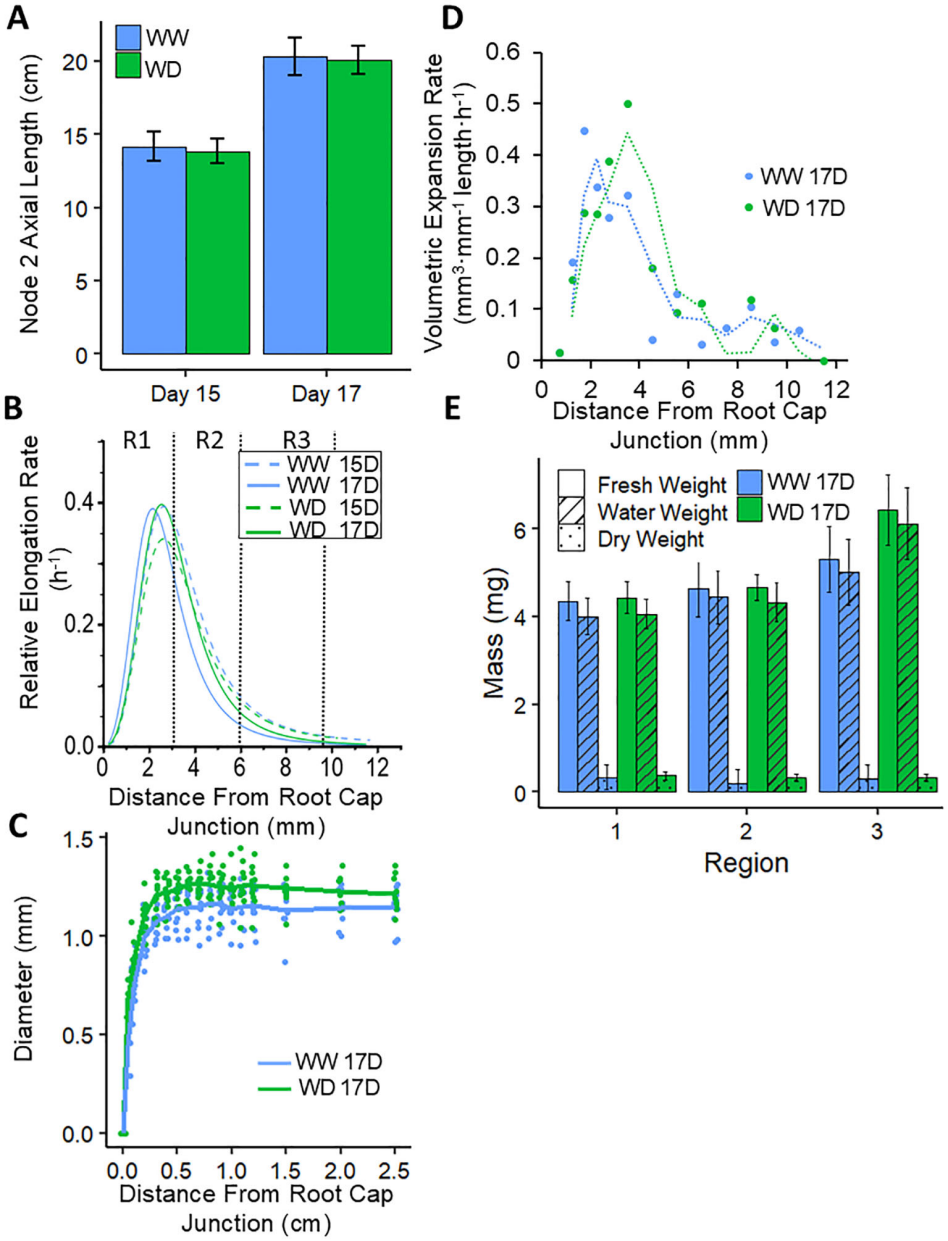
Kinematic profiling of the N2 root growth zone for WW and WD treatments. **(A)** Average N2 axial lengths per plant measured at day 15 and day 17 in the same experiment. Paired sample means were not significantly different at either time point (for both time points, *P* > 0.4 by Student’s *t-*Test). Root elongation rates were derived from the increase in length between the two harvests, and were combined with cortical cell length profiles on each day (**Supplementary Figure S3A**) to calculate displacement velocity profiles (**Supplementary Figure S3B**). **(B)** Derivatives of logistic curves fitted to the displacement velocity profiles provided the spatial distribution of relative elongation rate. R1, R2 and R3, as harvested for transcriptomic and metabolomic analyses, are indicated. **(C)** Spatial distribution of root diameter along the apical 2.5 cm on day 17. **(D)** Profiles of volumetric expansion rate on day 17, which were calculated from relative elongation rates **(B)**, radial plus tangential expansion rates (**Supplementary Figure S3C**), and profiles of root volume per unit length (data not shown); dotted lines denote a moving average regression fitted to the data points. **(E)** Fresh weight, water weight and dry weight of R1-R3 on day 17. Paired sample means were not significantly different for any weight in any region (for all comparisons, *P* > 0.3 by Student’s *t-*Test). Values in **(A, E)** are means ± SE; n = 9 roots from 6-7 plants (**A**, **B**; data from the same experiment), 22-25 roots from 9-10 plants **(C)**, 10-13 roots from 5-6 plants **(E)**.

Unless otherwise noted, plants were harvested after 12 days of growth in the divided-chamber system (i.e., on day 17). Disassembly of the growth apparatus was performed at ~95% RH under a green “safe” light. The nodal roots were excavated from the outer container by carefully removing each section and gently removing the surrounding soil. Immediately after excavation, node 1 (N1) and node 2 (N2) root tip Ψ_W_ were measured by isopiestic thermocouple psychrometry (Boyer and Knipling, 1965). The apical 20 mm region was sampled to include both the growth zone (approximately 12 mm; see **Figure 2B**) and some attached mature tissue. Because the growing region contains expanding cells, continued cell wall relaxation could potentially decrease tissue turgor following excision of the root. Therefore, the mature tissue was included to provide a source of water so as to avoid erroneously low water potential measurements (Nonami and Boyer, 1989). Leaf blade Ψ_W_ and N2 root tip osmotic potential (Ψ_S_) measurements were also made by isopiestic thermocouple psychrometry; root tip turgor (Ψ_P_) was calculated as the difference between Ψ_W_ and Ψ_S_ measurements. N1, N2 and node 3 (N3) numbers and lengths were measured. Seedling roots were recovered by washing the inner chamber contents on a mesh screen, and primary and seminal root lengths were measured (the primary root was severed above the scutellar node). Leaf areas were measured using a Li-3000A leaf area meter (LI-COR Environmental, Lincoln, NE, USA; licor.com).

In separate experiments, the spatial distribution of N2 root diameter (apical 2.5 cm) was measured using ImageJ from high-resolution photographs of freshly harvested roots, and the fresh weight (FW), dry weight (DW) and water content of three contiguous regions of the growth zone (see **Figure 2B**) were obtained by weighing root tip sections in a tared Eppendorf tube before and after drying at 65 °C overnight. Four to six plants per treatment were used.

### 2.4 Kinematic analysis

N2 root tips used for kinematic analysis were harvested from plants grown for 15 and 17 days and immediately placed into FAA fixative before vacuum infiltration and embedding into paraffin (Ruzin, 1999). The apical 2 cm of embedded root tips were prepared for light microscopy examination by microtome sectioning, staining with toluidine blue, and permanently mounting on microscope slides (IDEXX BioAnalytics, Columbia, MO, USA; idexxbioanalytics.com). High resolution images were stitched together to create a composite profile of the entire root tip and cortical cell length profiles were quantified using ImageJ (Schneider et al., 2012). Spatial distributions of displacement velocity (mm length h^-1^) and relative elongation rate (h^-1^, calculated as mm length h^-1^ mm^-1^ length) along the root growth zone were calculated from overall root elongation rates (calculated from the increase in length between days 15 and 17 of the roots used for cell length measurements) and cortical cell length profiles as described previously (Silk et al., 1989; Yamaguchi et al., 2010; Voothuluru et al., 2020). In brief, curves were fitted to displacement velocity values using the logistic curve fit ( $y=a2+\frac{a1-a2}{1+{\left(\frac{x}{x0}\right)}^{p}}$ ) function of the Origin statistical software package (OriginLab, Northampton, Massachusetts, USA) wherein R values exceeded 0.98 for all fit curves. The first derivative of displacement velocity with respect to position was plotted as the relative elongation rate. Average root elongation rates during the 48-h period encompassing the analyses were obtained from the difference between mean root lengths of plants harvested at 15 and 17 days. Spatial distributions of radial plus tangential expansion rates (h^-1^, calculated as mm^2^ h^-1^ mm^-2^) and volumetric expansion rates (mm^3^ mm^-1^ length h^-1^) were calculated as previously described (Sharp et al., 1988). Rates of cell flux (cells h^-1^) were calculated by dividing root elongation rates by final cell lengths (Silk et al., 1989).

### 2.5 RNAseq and data processing

N2 roots collected for RNA extraction were immediately placed in a high RH chamber and the growth zone was sectioned into three contiguous regions (see **Figure 2B**; distances are from the root cap junction): Region 1 (R1, 0–3 mm plus the root cap), Region 2 (R2, 3–6 mm), and Region 3 (R3, 6-9.5 mm) and flash frozen in liquid nitrogen. Only roots that fell within 1 standard deviation (SD) of the mean axial length value were collected; all qualifying roots from each plant were pooled. Five biological replicate samples for each of the treatments (WW and WD) were generated. Each sample was composed of the respective root regions obtained from four individual plants that were grown in different experiments. RNA was extracted from each replicated sample using the Qiagen RNAeasy plant kit. DNase treatment was performed according to the manufacturer’s protocol using TURBO DNase kit. After DNase treatment, RNA samples were assessed for quantity and concentration using a Qubit RNA assay kit (Thermo Fisher). All RNA samples assessed had high (≥8) RIN scores. RNAseq library construction and RNAseq sampling were performed by Novogene (novogene.com).

### 2.6 Metabolomic analysis

In separate experiments, N2 root tissues were collected as described for the RNA samples. Three replicates from pooled samples from each of the treatments (WW and WD) were used to obtain R1, R2, and R3 samples. Samples were flash frozen and ground in liquid nitrogen using a ceramic mortar and pestle, lyophilized and stored at -80°C. Aliquots for each sample (20 mg) were extracted in 400 mL of methanol containing recovery standards using an automated MicroLab STAR system (Hamilton Company). Extracts were subjected to global unbiased metabolic profiling by Metabolon (Morrisville, NC) using Ultrahigh Performance Liquid Chromatography-Tandem Mass Spectroscopy (UPLC-MS), both for positive and negative ions separately, and GC-MS followed by full-scan mass spectra to record retention time, molecular weight and MS/MS^2^ of all detectable ions as described (Oliver et al., 2011). Raw data was extracted, peak-identified, and QC processed using Metabolon’s hardware and software (metabolon.com). Metabolites were identified by automated comparison of the ion features in the samples to a reference library of chemical standard entries. A total of 569 compounds of known identity were identified in the three root regions. Median scaling was completed by dividing the raw area counts for each metabolite by the median value for the respective metabolite. Missing values for a given metabolite in a sample were imputed with the minimum observed value for that metabolite. Data was transformed using the natural log for statistical analyses. Differentially accumulating metabolites (DAMs), significantly different between WW and WD conditions in each root region, were identified using Welch’s two-way *t*-test. Resulting metabolites achieved statistical significance with a p-value<0.05, false discovery rate (FDR) q-value<0.10, and FC ≥1.5| and thus were deemed differentially accumulated.

### 2.7 Bioinformatics

Our RNAseq informatics analysis pipeline has been previously described (Wang et al., 2022), which leveraged a high-quality *de novo* FR697 transcript assembly (Sen et al., 2023). Functional annotation of transcripts was performed using the Michigan State University Rice Genome Annotation Project (https://plants.ensembl.org/biomart/). Metabolic pathway analysis was conducted using the Metabolomic Pathway Analysis tool within MetaboAnalyst 6 (https://www.metaboanalyst.ca/). The list of metabolites significantly altered in the given sample was entered into the tool and annotated using common compound names. To ensure accuracy, validation of identified metabolites was conducted through manual verification using HMDB, KEGG, and PubChem databases. Analyses were performed with the library specific to *Zea mays*, utilizing the Kyoto Encyclopedia of Genes and Genomes (KEGG) pathway database (http://www.genome.ad.jp/kegg/pathway.html). The significance of metabolite enrichment within pathways was determined using a statistical test based on hypergeometric distribution, with a threshold of raw p-values< 0.01 indicating significant enrichment. To account for multiple pathway testing, statistical p-values were further adjusted using FDR estimation. Pathway topological analysis assessed the importance of nodes within pathways using relative betweenness centrality, which in turn determined pathway impact.

Principal components analysis (PCA) for transcriptome and metabolome data was performed using the FactoMineR package (Lê et al., 2008) and GGPlot2 package (Wickham, 2016) for R (Team, R.C., 2013). Heatmaps were composed using the pheatmap package in the R computing environment (Kolde, 2019). GO enrichment analysis was performed using AgrigoV2.0 (Tian et al., 2017) and plotted as described (Bonnot et al., 2019). KEGG functional enrichment analysis for developmentally associated transcriptomic changes was performed using ShinyGO (Ge et al., 2020). Integration of metabolomic and transcriptomic data was performed using CornCyc 9.0 hosted at MaizeGDB (Caspi et al., 2014).

## 3 Results

### 3.1 A divided-container root growth system enables uniform and steady imposition of WD conditions to growing nodal roots

A divided-container root growth system (**Figure 1A**; **Supplementary Figures S1A, B**) was used to impose controlled WD conditions around the nodal roots independently of the seedling (primary and seminal) root system. The WD treatment was designed such that the water supply for transpiration was provided from the inner container which contained soil of a higher Ψ_W_ relative to the outer container. As a result, the outer container Ψ_W_ was maintained and the growing nodal roots were subjected to uniform and steady WD conditions during the course of the experiments.

A standard WD treatment was established that comprised outer and inner container soil Ψ_W_ of -0.9 MPa and -0.4 MPa, respectively. An outer container Ψ_W_ of -0.9 MPa was selected based on the study of Westgate and Boyer (1985), which showed that maize nodal root elongation was maintained at WW rates over this range of Ψ_W_ (Westgate and Boyer, 1985); preliminary experiments showed that the outer container soil Ψ_W_ did not significantly change during the 17-day experiments (**Supplementary Figure S1C**). An inner container soil Ψ_W_ of -0.4 MPa was selected from preliminary experiments which demonstrated that the soil Ψ_W_ remained uniform throughout the vertical dimension of the container over the course of the experiments following initial soil water treatments of FC, -0.25 MPa, or -0.4 MPa (**Supplementary Figure S1D**). These results indicate that the bulk of water that was utilized for transpiration was provided by the “plug” of FC soil in which the seedlings were transplanted, allowing the maintenance of steady soil Ψ_W_ around the majority of the seedling root system throughout the experiments. In contrast, the soil Ψ_W_ became stratified following initial treatment at -0.85 MPa. In both the -0.4 MPa and -0.85 MPa treatments, leaf area development was significantly inhibited compared with the FC control (**Supplementary Figure S1E**), as is typically observed in water-stressed plants. However, plants grown in the -0.85 MPa treatment consistently wilted at 15 days, indicating progressive stress development. To avoid potential complications arising from non-steady shoot water stress, the less stressful scenario obtained with a -0.4 MPa inner container, together with the -0.9 MPa outer container, was selected.

In the standard WD treatment, N1 and N2 root tip Ψ_W_ (apical 20 mm, encompassing the growth zone) were -0.9 and -0.75 MPa, respectively, but were not significantly different from each other (**Figure 1B**). The substantial reductions in root tip Ψ_W_ from the WW values illustrate that the treatment successfully imposed a significant water stress to the growing root tissues. In contrast, leaf Ψ_W_ was only approximately -0.5 MPa, compared with approximately -0.25 MPa in the WW treatment (**Figure 1B**). Taken together, these data indicate that nodal root tip water relations were influenced more by the water status of the surrounding soil than by that of the plant as a whole. In N2, the decrease in root tip Ψ_W_ was balanced by an equivalent decrease in Ψ_S_, resulting in maintenance of root tip Ψ_P_ (**Figure 1C**) and indicative of complete osmotic adjustment. Osmotic adjustment was not assessed in N1 roots. As already noted, leaf area was attenuated by approximately 50% in the WD treatment; this effect was consistent for mature and immature leaves (**Figure 1D**).

### 3.2 Nodal root elongation beyond the first emerging whorl is completely maintained under WD conditions

While the WD treatment resulted in shorter N1 root lengths (approximately 28% inhibition), axial elongation was fully maintained in N2 and N3 roots compared to the WW controls (**Figure 1E**). The average number of N1 and N2 roots per plant was slightly (5-7%) reduced by the WD treatment, whereas N3 root number was unchanged (**Supplementary Figure S2A**). Given our interest in root growth maintenance under WD conditions, we focused on N2 roots for the remainder of the study as a model for the study of growth maintenance mechanisms. While it is possible that subsequent whorls of nodal roots maintain elongation similarly, our divided-chamber growth system was calibrated for the study of the early nodal roots; investigation of subsequent whorls would require a larger apparatus and larger soil volume.

Seminal root elongation also was not altered in the WD treatment, whereas primary root length was reduced by approximately 27% (**Supplementary Figure S2B**). However, the soil water potential in the inner container of the WD treatment was only about -0.4 MPa throughout the experiment (**Supplementary Figure S1D**), In contrast, previous work on maize seedlings showed that seminal root elongation was more inhibited than primary root elongation at a water potential of -0.8 MPa (Sharp et al., 1988). Accordingly, we chose to focus on N2 nodal roots, which showed complete maintenance of elongation at a root tip water potential of -0.75 MPa (**Figures 1B, E**).

The overall rate of root elongation is determined by the rate of cell production from the meristem and the rate and duration of cell elongation. These parameters can be evaluated by kinematic analyses based on cell length profiles (Silk et al., 1989; Dowd et al., 2020; Voothuluru et al., 2020), which are useful to define the length of the growth zone, the spatial pattern of relative elongation rate within the growth zone, and the cell flux (the rate per cell file at which fully elongated cells leave the growth zone; under steady conditions, cell flux equals the rate of cell production from the meristem). Accordingly, a detailed kinematic analysis of the growing region of the N2 root was performed to assess whether the complete maintenance of N2 root elongation in the WD treatment was associated with maintenance of both cell elongation and cell production.

Axial lengths of N2 roots were measured at 15 and 17 days after germination (**Figure 2A**), from which overall root elongation rates were derived and showed that rates were not significantly different between the WW and WD treatments (WW, 3.08 cm day^-1^; WD, 3.13 cm day^-1^). Cortical cell length profiles were measured along the apical 12 mm (**Supplementary Figure S3A**) and were similar between the treatments or timepoints, indicating a steady pattern of cell expansion in both treatments. Average cell lengths were not different from 10–12 mm (as measured from the root cap junction), indicating that the growth zone comprised the apical cm of the root. The root elongation rates and cell length profiles were used to calculate displacement velocity profiles (representing rates of cellular displacement through the growth zone; **Supplementary Figure S3B**) and relative elongation rate profiles (derivative of displacement velocity with respect to position; **Figure 2B**), both of which were similar between the treatments and timepoints. Accordingly, the results show that the spatial and temporal patterns of cellular elongation throughout the root growth zone were unchanged by the WD treatment. In both treatments, the relative elongation rate exhibited rapid acceleration to reach peak rates by approximately 2.5 mm from the root cap junction, followed by deceleration leading to cessation of cell elongation at approximately 10 mm (**Figure 2B**). The relative elongation rate profile was used to define three distinct zones within the N2 root growth zone that were harvested for transcriptomic and metabolomic analyses (distances from the root cap junction): R1 (0–3 mm plus the root cap), encompassing the meristem and region of acceleration and maximal elongation; R2 (3–6 mm), where cells are decelerating from peak elongation; and R3 (6-9.5 mm), where the cells are approaching cessation of elongation.

Rates of cell flux were calculated by dividing overall root elongation rates by final cell lengths. Since both parameters were unchanged between the WW and WD treatments (**Figure 2A**; **Supplementary Figure S3A**), rates of cell flux were also similar between the treatments, with values of 8.18 and 7.68 cells h^-1^, respectively (averages of days 15 and 17). Thus, the results indicate that rates of both cell production in the meristem and cell elongation throughout the growth zone were maintained under WD in N2 nodal roots.

During growth, roots expand in width as well as length. Measurement of root diameter profiles showed that the WD treatment resulted in N2 roots that were slightly wider throughout the apical 2 cm (**Figure 2C**). By combining the spatial distributions of displacement velocity and root radius, profiles of radial plus tangential expansion rate were obtained, which represent the rate of increase in cross-sectional area with distance from the root cap junction. In both the WW and WD treatments, rates were maximal closest to the root cap junction, reflecting the conical shape of the root tip, and then progressively decelerated until approximately 12 mm; rates were slightly increased in the WD compared to the WW treatment at all locations (**Supplementary Figure S3C**). The relative rates of elongation and radial plus tangential expansion are the essential components for determining the overall rate of volumetric expansion along the growth zone (Silk and Haidar, 1986; Silk et al., 1986). Addition of the two parameters gave the relative rates of volumetric expansion, which were then multiplied by root volume per unit length to calculate profiles of volumetric expansion rate (calculated as mm^3^ mm^-1^ length h^-1^). The results showed that in both the WW and WD treatments, the peak rate of volumetric expansion was observed between 2–4 mm from the root cap junction (**Figure 2D**). However, the peak rate was shifted basally by approximately 1.5 mm in the WD treatment and remained slightly elevated compared to the WW treatment throughout the middle section of the growth zone, corresponding to R2 in our subsequent analyses.

Consistent with the maintenance of root width in the WD treatment, FW and DW along the root tip were not statistically different between the WW and WD treatments (**Figure 2E**). As a result, water weights per region were also similar between the treatments, although mean water weight was slightly greater for WD plants in R3. This is consistent with the increased root diameter of WD roots in this region (**Figure 2C**).

### 3.3 WD elicits substantial changes in metabolite abundance in the N2 root growth zone

To investigate the metabolic responses that were associated with the maintenance of cellular elongation and volumetric expansion throughout the growth zone of water-stressed N2 roots, the metabolome was surveyed in R1–3 of the growth zone of roots growing under WW and WD conditions. It is important to divide the growth zone into different regions for study because in addition to the spatially varying profile of cell expansion rates (**Figures 1B, D**), it is well established that the growth zone encompasses distinct tissue types and cellular activities (Sharp et al., 1988; Voetberg and Sharp, 1991; Zhu et al., 2007; Voothuluru and Sharp, 2013). For example, the meristem and root cap are contained within R1 whereas root hair initiation begins around R3. Therefore, a comprehensive understanding of the molecular processes that enable continued expansive growth could be masked if assaying metabolite abundances on a whole growth zone basis rather than dividing the root into different regions where these activities might be reasonably expected to differ.

This untargeted metabolic analysis revealed a broad range of differentially accumulated metabolites (DAMs) across all regions of WD-treated roots, including increased sugar and sugar alcohol accumulation, reduced accumulation of sugar-phosphates, and altered amino acid accumulation (**Supplementary Dataset S1**). WD-treated roots accumulated the hallmark stress-induced osmolytes betaine and proline at over 15-fold higher levels compared to the WW roots, and ABA levels were increased approximately four-fold (**Supplementary Figure S4**). KEGG enrichment analysis indicated that the most enriched pathways in the WD-treated roots were related to glutamate, aspartate and glutathione metabolism, while the most significantly reduced pathways were related to purine, pyrimidine, and phospholipid metabolism (**Supplementary Figure S5**).

**Figure 3:**
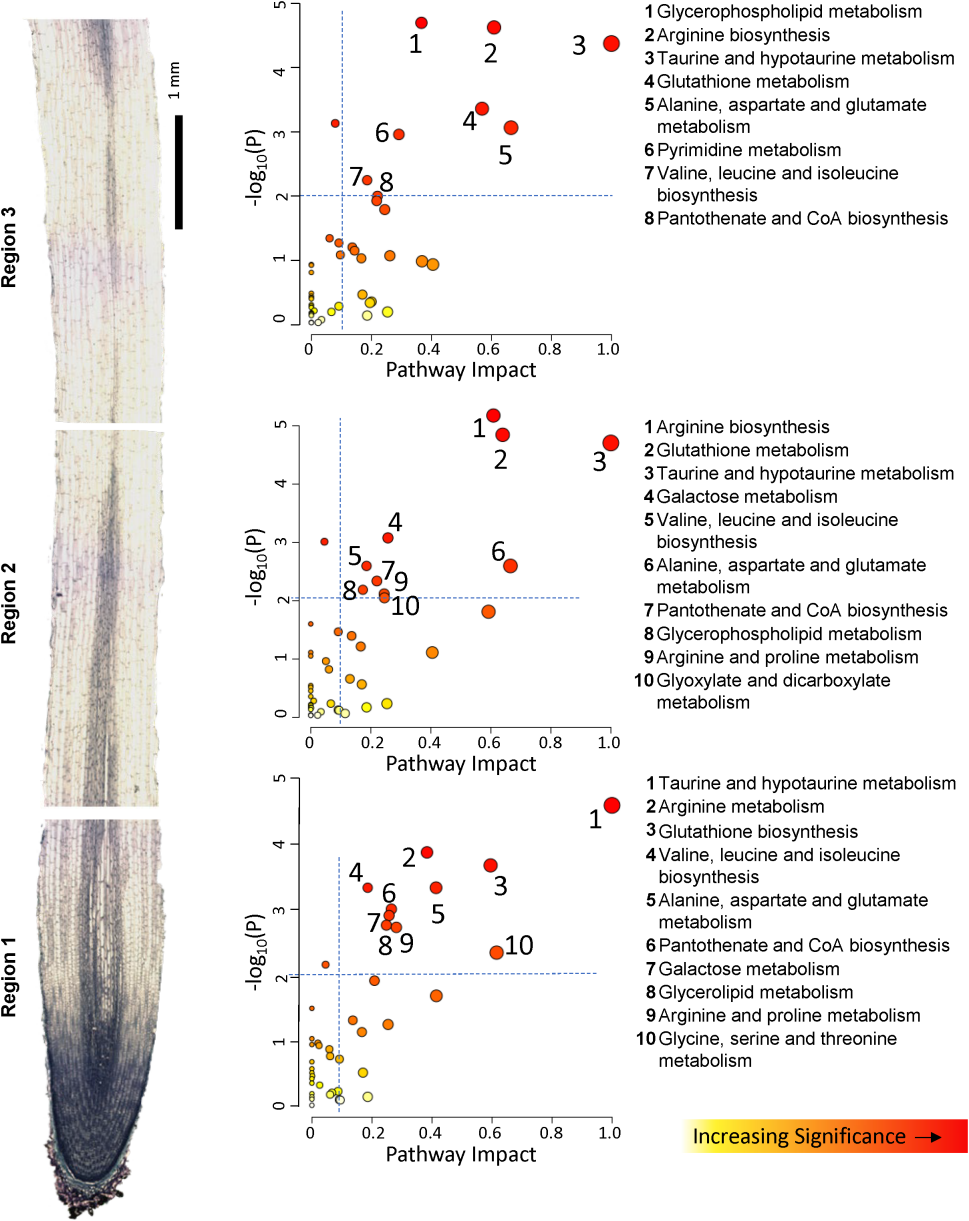
Overview of metabolic pathways associated with significantly increased metabolite abundances in R1, R2 and R3 under WD compared to WW conditions. Pathway impact (x axis) signifies the topology-based enrichment of a given KEGG pathway, accounting for the interconnections of related metabolites in a given pathway. Y axis signifies FDR corrected statistical significance. See **Supplementary Table S1** for statistical details of pathway identification. Increasing circle sizes denote greater pathway impact score, color denotes increasing significance.

**Figure 4:**
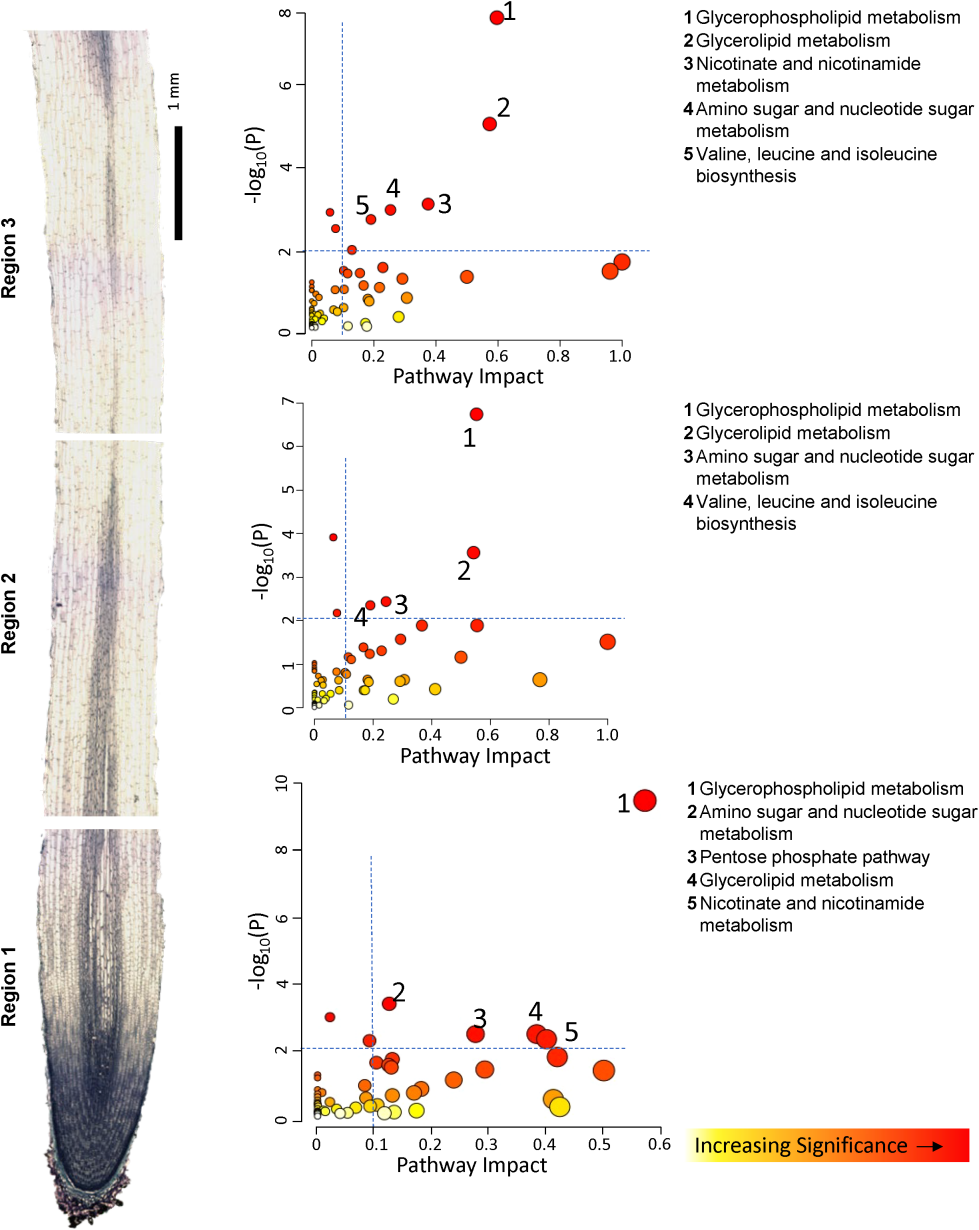
Overview of metabolic pathways associated with significantly decreased metabolite abundances in R1, R2 and R3 under WD compared to WW conditions. Pathway impact (x axis) signifies the topology-based enrichment of a given KEGG pathway, accounting for the interconnections of related metabolites in a given pathway. Y axis signifies FDR corrected statistical significance. See **Supplementary Table S1** for statistical details of pathway identification. Increasing circle sizes denote greater pathway impact score, color denotes increasing significance.

To more completely evaluate changes in entire metabolic pathways which were associated with the response to WD, we employed the topology-based pathway analysis tools embedded in the MetaboAnalyst 6 software, in which the DAMs were used to determine which pathways were altered in each region of the growth zone and which of these dominate the metabolic response to the WD stress both with respect to increased and decreased metabolite abundance (Wieder et al., 2021). Pathways were considered dominant if they exhibited significance levels of -log_10_ p-values greater than 2, pathway impact values exceeding 0.1, and false detection rate (FDR) values below 0.1. The predominant pathways associated with increased metabolite abundances in the metabolic response to WD in each of the three regions of the growth zone were similar, with amino acid metabolism dominating the response (**Figure 3**; **Supplementary Table S1**). The specific DAMs identified for each implicated pathway are listed in **Supplementary Table S2**. All three regions showed that the taurine, arginine, proline, alanine/aspartate and valine/leucine/isoleucine pathways were significantly enriched among increasing metabolites in response to WD. All three regions also appeared to mount a significant glutathione metabolism response along with alterations in pantothenate and coenzyme A (CoA) biosynthesis pathways and glycerophospholipid metabolism. Beyond these common pathways, several region-specific pathways were observed among the significantly accumulating metabolites. These included glycine/serine/threonine metabolism in R1, glyoxylate and decarboxylate metabolism in R2, and pyrimidine metabolism in R3. The pathways associated with decreased metabolite abundances were fewer in number, and glycerophospholipid metabolism was the most significant pathway in all regions. Glycerolipid metabolism and amino sugar and nucleotide sugar metabolism were also among the pathways associated with decreased metabolites in all regions, while nicotinate and nicotinamide metabolism and valine biosynthesis pathways were significantly reduced in R1 and R3, but not R2 (**Figure 4**; **Supplementary Table S1**). In a somewhat contradictory manner, the analysis indicated that valine, leucine, and isoleucine metabolism significantly contributed to metabolite declines in R2 and R3 (**Figure 4**) even though their metabolism also significantly contributed to increases in metabolite abundances in all regions of the growth zone (**Figure 3**). In general, the metabolites showing increased abundances are primarily intermediates of isoleucine biosynthesis, whereas the metabolites with decreased abundances are mostly intermediates of leucine biosynthesis (**Supplementary Table S3**).

### 3.4 Region-specific transcriptomic responses to WD in the N2 root growth zone

To investigate the potential for regulation of transcript accumulation to underpin the metabolite accumulation patterns in the N2 growth zone under WD stress, the response of the transcriptome to the WD treatment was assessed in the different regions. The resulting dataset mapped 45,764 unique transcripts (**Supplementary Dataset S2**) and from these a total of 1,019 differentially accumulating transcripts (DATs), exhibiting at least a log_2_FoldChange (FC) ≥ |2| in abundance between WD-treated and WW root samples, were identified (**Supplementary Dataset S3**). The R1 transcriptome responded to WD stress with relatively few DATs compared to R2 and R3 (104 DATs for R1 compared to 550 and 365 for R2 and R3, respectively). R2 exhibited the largest number of DATs, the majority of which (nearly 70%) exhibited a decrease in abundance (**Table 1**).

**Table 1:** Number of DATs with increased (up) and decreased (down) abundance in R1, R2 and R3 of the N2 root growth zone under WD compared to WW conditions (at a threshold of significance of |log_2_FC| ≥2 or |log_2_FC| ≥1).

Threshold	R1			R2			R3		
	Total DAT	DAT *up*	DAT *down*	Total DAT	DAT *up*	DAT *down*	Total DAT	DAT *up*	DAT *down*
|log_2_ FC |≥ 2	104	63	41	550	168	382	365	182	183
|log_2_ FC |≥ 1	195	104	91	1229	384	845	889	396	493

To understand the effect of WD on the transcriptome of each region of the growth zone, DATs from each region were examined by gene ontology (GO) enrichment analysis. GO enrichment described 68 and 59 categories among the populations of positively and negatively accumulating transcripts, respectively (**Supplementary Figure S6**). In R1, with the fewest number of enriched GO categories for both increased and decreased transcript abundances, those transcripts that increased in abundance appear to be associated primarily with ion transport, response to various abiotic factors, and response to ABA. Those transcripts that decreased in abundance in R1, though fewer in number, were largely involved with various aspects of metabolic regulation. For R2, which had the largest transcriptomic response to the WD stress, the GO enrichment analysis indicated a very broad transcriptomic response in both the increased and decreased abundance transcript pools. For the transcripts that increased in abundance, the categories that contained both transcripts with high read counts and transcripts exhibiting significantly higher differential levels of abundance were those involved in response to abiotic stress and other abiotic stimuli, including those that respond to water deprivation. R2 also exhibited a much higher representation of transcripts that respond to ABA. Other categories that were well represented in the more abundant transcript pool were those involved in ion transport, carbohydrate catabolism, and developmental and morphogenesis processes. The categories of transcripts that decreased in abundance in the transcriptome response were also very broad. These were populated by a large percentage of the GO categories recorded, the most prominent (highly abundant transcripts and number of genes) being the categories for responses to chemical and general stimuli. The R3 GO enrichment analysis largely mirrored that of R2, but with less intensity with regards to overall transcript abundance. R3 did differ from the other two regions in one significant set of GO categories for increased abundance of transcripts, namely the categories that relate to changes in epidermal and cell wall related processes.

Given the unique growth activities that predominate regions 1–3 of the N2 growth zone, as described by our kinematic analysis (**Figures 2B–D**), we assessed changes to the transcriptome associated with the developmental maturation program inherent in the elongation of the nodal root. Comparing the transcriptomes of R1 and R2 as well as R2 and R3, within a treatment, revealed DATs associated with the maturation processes (**Supplementary Dataset S4**). In WW roots, the DATs significantly enriched (log_2_ FC ≥ |2|) in R1 (which encompassed the meristem) compared to R2 were primarily associated with basic cellular/nuclear functions or transcription and translation machinery (including such KEGG functional categories as ‘Ribosome’, ‘Ribosome biogenesis’, ‘DNA replication’, ‘Mismatch repair’, ‘RNA degradation’, and ‘Homologous recombination’; **Supplementary Figure S7A**). Beyond these activities, which are indicative of cell division processes, biosynthesis and metabolic activity were indicated by the functional categories ‘Fatty acid biosynthesis’ and ‘Fatty acid metabolism’, ‘Alanine, aspartate, and glutamate metabolism’, and others. The transition from R1 to R2 is associated with cells beginning to decelerate from their peak elongation rate (**Figure 2B**). The transition from meristematic and peak elongation to maturation was reflected by those DATs significantly enriched in R2 as compared to R1, which encompassed metabolic and biosynthesis associated transcripts as well as signaling and signal transduction functional categories (**Supplementary Figure S7A**). Using a pairwise comparison, the transition from R1 to R2 in WW roots was associated with 3453 DATs that were enriched in R1 as compared to R2, and 3271 DATs that were enriched in R2 as compared to R1. Only two transcripts were significantly different (log_2_ FC ≥ |2|) in the pairwise comparison between R2 and R3. These transcripts, a cupredoxin superfamily protein and a protein kinase, were both enriched in R2 compared to R3 (**Supplementary Dataset S4**).

The transcriptomic changes associated with the R1 to R2 transition in the WD-treated roots (**Supplementary Figure S7B**) were similar to those in WW roots. Twenty functional categories were enriched in WD R1 compared to WD R2, which were not also enriched in the WW comparison. These included the KEGG functional categories ‘Base excision repair’ and ‘Nucleotide metabolism’, as well as ‘Biosynthesis of amino acids’, ‘Spliceosome’ and ‘Biosynthesis of secondary metabolites’, which were the three least enriched functional categories in WD R1 with respect to WD R2. The enriched DATs in WD R2 compared with WD R1 were likewise very similar to the corresponding WW comparison, although absent were the categories ‘Flavonoid biosynthesis’, ‘Diterpenoid biosynthesis’, and ‘Glutathione metabolism’. Unlike the WW R2 to R3 transition, 160 transcripts were significantly enriched in WD R2 compared to R3 with 406 enriched in R3 with respect to R2 (**Supplementary Dataset S4**). Of these DATs, KEGG enrichment analysis identified no functional categories enriched in those transcripts significantly accumulating in R2 as compared with R3. The functional categories ‘Phenylalanine metabolism’, ‘Phenylpropanoid biosynthesis’, ‘Biosynthesis of secondary metabolites’, and ‘Metabolic pathways’ were enriched in transcripts significantly accumulating in WD R3 as compared with R2.

### 3.5 Transcriptomic underpinnings of hormone changes during WD

The regulation of root growth during WD involves interactions between ABA and several other phytohormones including ethylene and auxin (Kim and Mulkey, 1997; Spollen et al., 2000; Xu et al., 2013; Rowe et al., 2016; Zhang et al., 2022). The WD treatment in this study induced significant accumulation of ABA in all regions of the N2 root growth zone (**Supplementary Figure S4**). Accordingly, we explored transcriptomic changes associated with hormone-related signaling. While the WD treatment induced a near five-fold increase of ABA compared to WW roots, ABA biosynthesis appeared to be attenuated at the transcriptomic level (**Supplementary Figure S8A**). The rate limiting step of ABA synthesis is determined by the activity of 9-cis-epoxycarotenoid dioxygenase (NCED) (Xiong and Zhu, 2003), for which three transcripts (isozymes) were mapped with an average normalized transcript count above 1 in any region or treatment combination (**Supplementary Dataset S2**; **Supplementary Figure S8**). A single transcript, corresponding to the causal locus in the maize *viviparous14* (*vp14)* mutant (Tan et al., 1997; Matusova et al., 2005; Venado et al., 2017), was enriched during WD, although not exceeding the threshold of statistical significance used in this study (log_2_ FC ranging from 1.24-1.64). The putative *Vp14* transcript was the least abundant transcript of the three NCED isozymes in all regions of the growth zone of WW roots, and the only one to increase in abundance during WD. All corresponding transcripts for the following biosynthetic step, xanthoxin dehydrogenase, were significantly attenuated. Beyond the biosynthesis of ABA, transcriptomic data suggested attenuation in ABA degradation. The three most abundant transcripts encoding isozymes of abscisic acid 8-hydroxylase, which facilitates catabolism of 2-*cis*-abscisate to a phaseic acid intermediate, were all attenuated although below the threshold of statistical significance. The data also suggested alterations in the perception of ABA in the growth zone, with changes in transcript abundances for associated signal transduction components: the PYL receptors, interacting PP2C protein phosphatases, and the SnRK2 protein kinases, which comprise the core ABA signaling network (Umezawa et al., 2010; Bhaskara et al., 2012; Wang et al., 2018). Transcripts for all but two ABA receptors were observed (mean transcript count above 1 in all regions by treatments), and all but the low abundance transcript PYL6 were attenuated by WD (**Supplementary Figure S8B**). In particular, PYL7 was the most abundant ABA receptor transcript in R2 and R3 and was significantly attenuated by WD. In general, transcripts of SnRK2 protein kinases and the interacting PP2C protein phosphatases increased in abundance during WD. Transcript abundances of PRH3 and PRH4 increased significantly in R2 and R3 while PRH8 transcript increased in R3; PRH10 transcript significantly decreased in abundance in the WD stressed R3. SnRK2.5 was the only SnRK2 kinase transcript for which abundance diminished in response to WD and the magnitude of change was below statistical significance.

While ethylene and its immediate precursor ACC require specialized assays and, therefore, were not quantified in the untargeted metabolomic analysis, the abundances of transcripts encoding the biosynthetic enzymes of the pathway along with the reduced abundance of the precursors to ACC and the byproduct of ACC production, 5′-methylthioadenosine, suggest that ethylene production in the N2 root growth zone was likely attenuated during the WD treatment (**Supplementary Figure S9**).

To further explore the transcriptomic response to WD in the N2 root growth zone, we surveyed the abundances of transcription factor (TF) transcripts. Transcripts for 675 TFs were considered present by exhibiting a mean transcript count ≥5 in at least one region by treatment combination; of these, 93 exhibited a log_2_FC ≥1.2, which was used as a threshold for identifying significantly accumulating TFs (**Supplementary Dataset S5**). In general, little correlation was observed between TF family and change in TF transcript abundance in response to WD, with most TF transcripts clustering on or about the medial line denoting 1:1 transcript abundance in WW and WD conditions (**Supplementary Figure S10**). Three TF families appeared to be exceptions, and exhibited non-linear transcript abundance: transcripts for the AP2/ERF family and C2C2-DOF family TFs appeared consistently lower in abundance in all regions of WD-treated roots compared with the WW controls, whereas transcripts for orphans, i.e. TF transcripts not categorized as members of any other TF family (Arendsee et al., 2014; Jiang et al., 2022), were more abundant in the WD treatment. Further investigation of ethylene-responsive TF transcripts revealed that except for a small number of low abundance transcripts, the DREB, AP2, and ERF subfamilies were attenuated by WD (**Supplementary Figure S11**).

The untargeted metabolomic survey indicated that in the WD-treated roots, auxin (indoleacetate, IAA) levels were unchanged in R1 but significantly increased in R2 and R3 (92% and 75% respectively, **Supplementary Dataset S1**). Transcript abundances of the enzymes in the IAA biosynthesis pathway were slightly enriched although below statistical significance (maximum log_2_ FC change ≤ 1) in R2 and R3 but not R1, which correlated with the respective changes in IAA abundance. Transcripts were detected for only a fraction of the isozymes catalyzing the tryptophan to IAA biosynthesis pathway; transcript abundances of *Vegetative tassel2* (*Vt2*), tryptophan-oxoglutarate aminotransferase, and indole-3-pyruvate monooxygenase were increased in the WD-treated roots, which may suggest the primary mechanism of synthesis occurred via these steps rather than through the tryptamine to indoleacetonitrile route (**Supplementary Figure S12A**). A survey of the transcripts for the components of auxin signaling, including the PIN-FORMED (PIN) family of auxin efflux carriers, AUX/IAA transcriptional repressors, auxin response factors (ARF) and small auxin upregulated RNAs (SAUR), suggested that auxin perception and signaling were minimally affected by transcript abundance in R1 compared to R2 and R3. Moreover, transcript abundance data suggested that these processes are perhaps facilitated by a small number of genes within each step of this signaling pathway (**Supplementary Figures S12B, C**).

### 3.6 The N2 root growth zone metabolome exhibits a more significant WD response than the transcriptome

**Figure 5:**
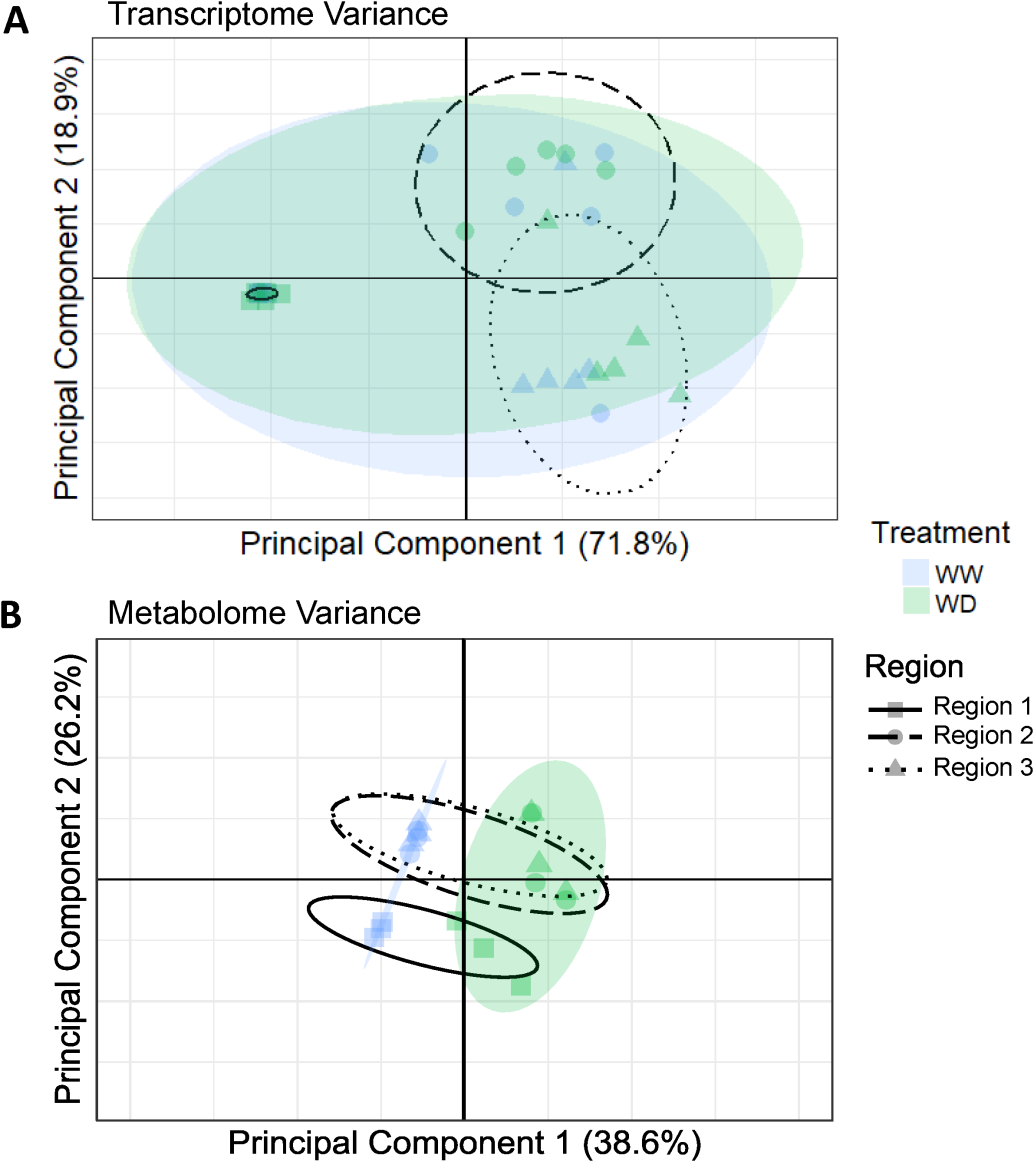
**(A)** Principal component analysis (PCA) clustering of RNAseq reads in R1, R2 and R3 samples of roots grown under WW and WD conditions. Principal Component (PC) 1 and PC2 encompass approximately 90% of the variation between all samples. 90% confidence intervals for treatment and region variables are represented by colored or dashed ellipses, respectively. **(B)** PCA clustering of metabolite abundances in R1, R2 and R3 samples of roots grown under WW and WD conditions. PC1 and PC2 encompass approximately two-thirds of total variation between all samples. 95% confidence intervals for treatment and region variables are represented by colored or dashed ellipses, respectively.

Principal components analysis (PCA) was performed for both the transcriptome and metabolome (**Figure 5**). The first two PCs explained approximately 90% of total transcriptomic variance or 65% of total metabolomic variance. While R1 appeared distinct in both the transcriptome and metabolome PCAs, the transcriptome clustered predominantly by region and did not resolve a treatment effect, whereas the metabolome clustered primarily by treatment along the PC1 axis, and then further resolving R1 from R2 and R3 along the PC2 axis. Aquaporin transcripts comprised 10 of the top 20 contributors to PC1 and PC2 loadings of the transcriptome PCA, contributing more than 50% of total variance, including more than 25% contribution from *tip1* transcripts (**Supplementary Figure S13A**). More than half of all metabolome PCA variation was accounted for by proline accumulation (**Supplementary Figure S13B**).

### 3.7 Metabolic and transcript abundance control of amino acid accumulation during WD

Given the contrast between the N2 root growth zone transcriptome, which appeared primarily governed by maturation programming with a less significant WD effect, and the nodal root metabolome, which exhibited a strong WD treatment effect and a much less significant maturation effect, we sought to determine to what extent the change in abundance of significant metabolites in response to WD is coordinated via transcriptomic adjustment, versus predominantly metabolic control. Recent work showed that metabolic control rather than transcript abundance was the principal mode governing the WD stress response in the primary root of the same maize genotype, FR697, as used in the present study (Kang et al., 2023). Given that elongation of the primary root is more sensitive to WD than the uninhibited N2 nodal roots, we hypothesized that 1) the metabolomic response to WD in the nodal root may involve further transcriptome coordination than the primarily metabolic control observed in the primary root, at least for the major metabolomic responses; and 2) the major metabolomic responses to WD in the nodal root are likely to differ substantially from the primary root.

**Figure 6:**
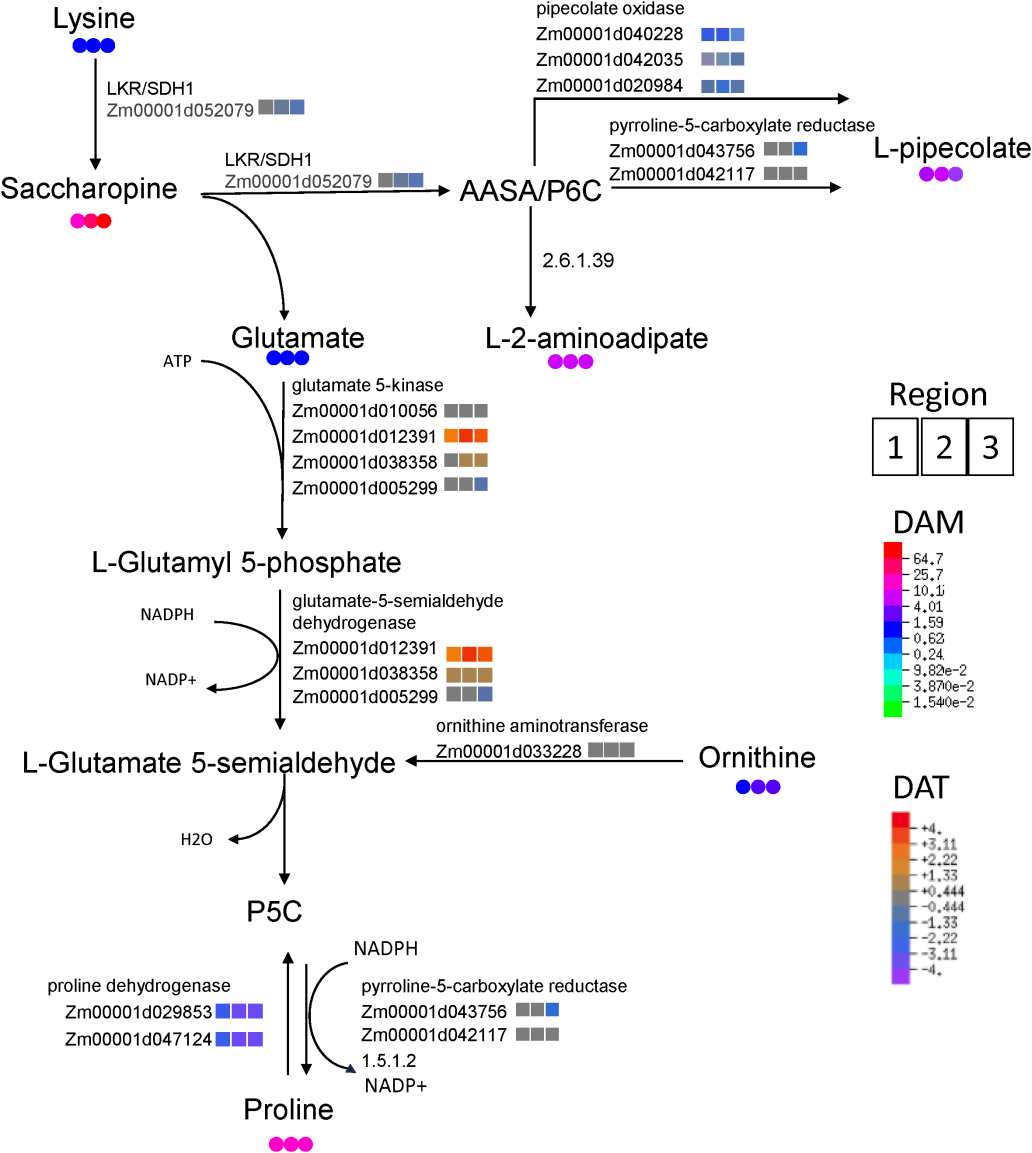
Integration of transcriptomic and metabolomic data shows transcriptional coordination of key steps in proline biosynthesis by the saccharopine pathway in R1, R2 and R3 (left to right, respectively). Colored circles indicate metabolite fold-changes of WD compared to WW (value >1 indicates enrichment in WD compared to WW). Colored squares indicate log_2_ fold-change between transcript abundances of WD compared to WW (positive value indicates enriched in WD relative to WW).

The above aims were pursued via integrating metabolite abundances with corresponding transcript abundances for key genes in the associated pathways. This approach was taken with the highly impacted pathways identified among significantly accumulating metabolites (**Figure 3**), as well as for proline which accounted for the majority of variance in the metabolome PCA (**Supplementary Figure S13B**). Moreover, proline and saccharopine were among the most significantly accumulating metabolites identified in the untargeted metabolomic analysis (**Supplementary Dataset S1**); indeed, saccharopine was the metabolite most responsive to WD (approximately 65-fold higher than WW in R3). Therefore, we explored the metabolic pathway which might link these two amino acids and explain their mechanism of significant accumulation during WD. Saccharopine accumulation from lysine appeared to occur without an increase in transcript abundance for the corresponding enzyme (LKR/SDH1). In contrast, the enzymatic steps from saccharopine to the proline precursor glutamyl semialdehyde involved significant increases in the major transcripts corresponding to associated enzymes, glutamate 5-kinase and glutamate-5-semialdehyde dehydrogenase. Transcripts encoding proline dehydrogenase (which converts proline to pyrroline-5-carboxylate, P5C) were significantly attenuated by WD (**Figure 6**). Furthermore, transcripts encoding pipecolate oxidase, which facilitates the conversion of saccharopine through a competing (non-proline producing) pathway, were significantly attenuated by WD. Although transcriptomic adjustments were observed for proline synthesis, similar concordance between transcriptomic and metabolomic WD response was not observed for the synthesis of arginine, alanine, valine or isoleucine (**Supplementary Figures S14A–C**). A small enrichment was observed for those transcripts corresponding to the metabolism of aspartate to malate, although neither metabolite accumulated significantly during WD (**Supplementary Figure S14E**). However, transcript abundance responses to WD were observed at key steps for some of these pathways: the final step of arginine degradation to glutamate by pyrroline-5-carboxylate dehydrogenase was increased by WD, while transcripts encoding glutamate synthases (which facilitate the conversion of glutamate to glutamine and oxoglutarate) were attenuated and transcripts encoding glutamate decarboxylase A, which converts glutamate to aminobutanoate, were enriched by WD (**Supplementary Figure S14D**). Taken together, these changes may suggest a coordinated reprograming of metabolic flux to shunt glutamate to 4-aminobutanoate. In addition to altered glutamate metabolism, a paradoxical relationship was observed between the transcriptomic response corresponding to the enzymatic steps of leucine from isopropylmalate (**Supplementary Figure S14C**). While isopropylmalate accumulated significantly during WD, and leucine along with its immediate precursor methyl oxopentanoate were substantially reduced in abundance, transcripts corresponding to the first two enzymatic steps of leucine synthesis from isoproylmalate were significantly enriched during WD.

### 3.8 Metabolic and transcript abundance control of ROS homeostasis during WD

Reactive oxygen species (ROS) accumulate at high levels during abiotic stress, with possible signaling roles, but they can be damaging to cells if ROS homeostasis is not maintained (Miller et al., 2010; Baxter et al., 2014; Sun et al., 2020). While the change in proline abundance in WD-treated N2 roots did not vary substantially across the growth zone, reduced glutathione (GSH) accumulated most dramatically in R2 and R3 (8.7- and 25-fold, respectively), with oxidized glutathione (glutathione disulfide; GSSG) accumulation varying from 5.9- to 8.6-fold across the growth zone. Unlike glutathione synthesis, the transcripts encoding enzymes in the glutathione-peroxide reactions exhibited increased abundance in R2 and R3 (**Supplementary Figure S15A**). WD-induced metabolic and transcriptomic changes suggest that the regeneration of GSSG may have occurred both metabolically (that is, without changes in associated transcripts) via dehydroascorbate reductase in the glutathione cycle, as well as through the coordinated transcriptomic adjustment of glutathione peroxidase and glutathione reductase (**Supplementary Figure S15A**).

**Figure 7:**
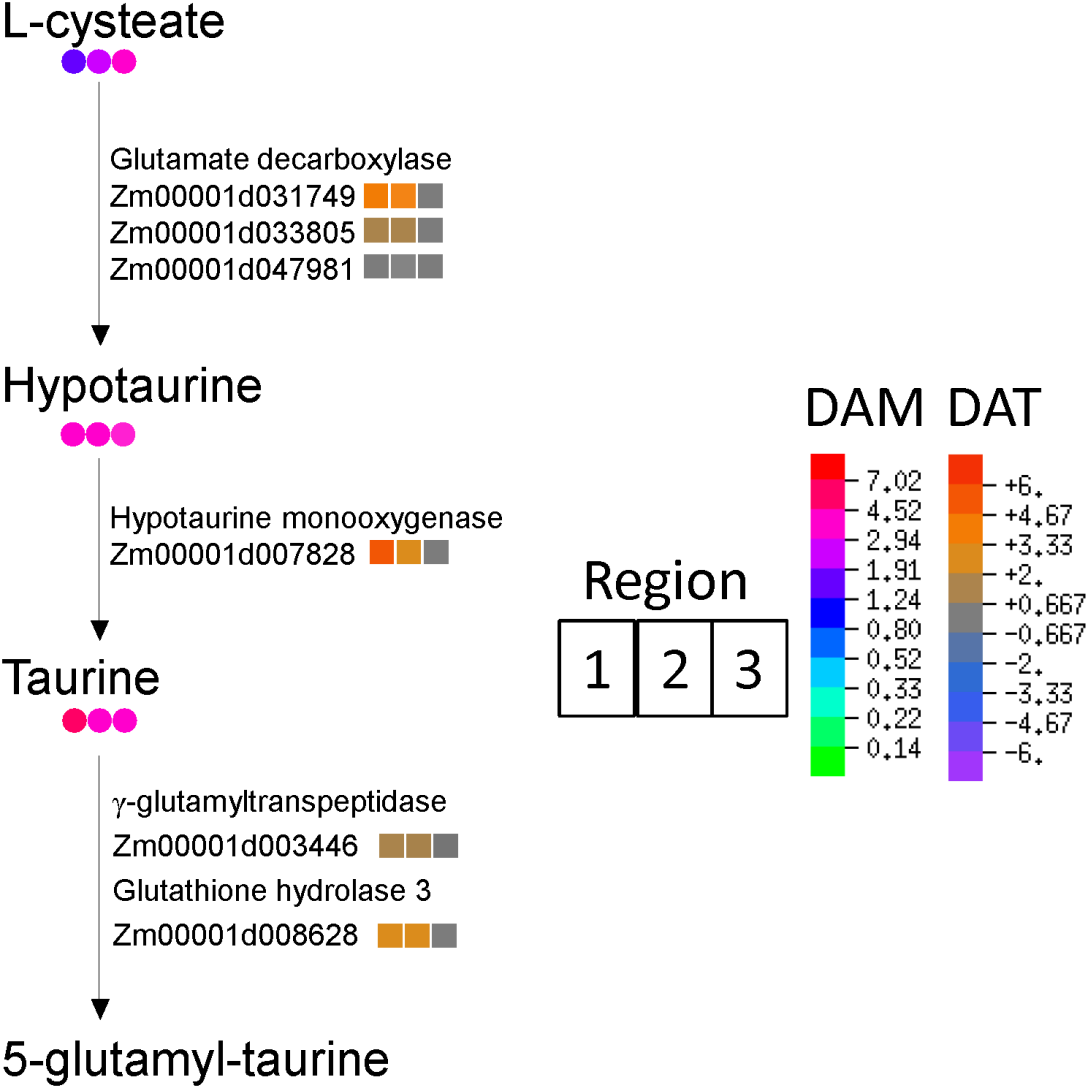
Biosynthesis of taurine and hypotaurine in R1, R2 and R3 (left to right, respectively). Colored circles indicate metabolite fold-changes of WD compared to WW (value >1 indicates enrichment in WD compared to WW). Colored squares indicate log_2_ fold-change between transcript abundances of WD compared to WW (positive value indicates enriched in WD relative to WW).

Taurine and hypotaurine metabolism were among the most impacted pathways associated with significantly increasing antioxidant metabolites in the WD-treated roots (**Figure 3**). Increased transcript levels were observed for all the enzymes that catalyze the steps of taurine synthesis from L-cysteate and conversion of taurine to 5-glutamyl-taurine in R1 and R2; concomitantly, taurine and hypotaurine increased in abundance in all regions of the growth zone by 3.5 to 7.0-fold (**Figure 7**).

KEGG pathway enrichment of metabolomic data (**Supplementary Figure S5**) and pathway impact analysis (**Figure 4**) both suggested that nicotinate and nicotinamide metabolism was overrepresented among metabolites reduced in abundance by WD. While WD increased the abundance of NAD^+^ in R3, this regeneration of NAD^+^ likely occurred via shunting of nicotinate, β-nicotinate D-ribonucleotide, and β-D ribofuranosyl nicotinamide (**Supplementary Figure S15B**). While WD significantly attenuated transcripts encoding nicotianamine synthase, preserving carbon flux for competing pathways, the surmised metabolite regeneration of NAD^+^ from nicotinamide and nicotinate species was not correlated with significantly increased abundance of associated enzymatic transcripts.

### 3.9 Phospholipid metabolism is mostly attenuated during WD

Metabolite abundances indicate that WD imparted significant reorganization of phospholipid metabolism in the elongating region of N2 roots. KEGG enrichment suggested that metabolism of phospholipids and related fatty acids, including diacyl glyerophospholipid, hydroxy fatty acids, and galactolipids, was significantly repressed in the N2 root growth zone during WD (**Supplementary Figure S5**). Pathway impact analysis identified glycerophospholipid metabolism as the most impactful metabolic pathway associated with significantly decreasing metabolites for all regions of the N2 growth zone (**Figure 4**). Glycerolipid and glycerophospholipid metabolism were also among the most impactful metabolic pathways associated with significantly accumulating metabolites in regions R1 and R2-R3, respectively (**Figure 3**). To further investigate this response, we queried the abundances of all transcripts encoding enzymes involved in galactolipid biosynthesis, glycolipid desaturation, free fatty acid biosynthesis, phospholipid biosynthesis, and biosynthesis of phosphatidylcholine, phosphatidylethanolamine and phosphatidylglycerol (**Supplementary Figure S16**). Among the 157 queried transcripts, six exhibited statistically significant changes in abundance (log_2_FC ≥ |2|) for one or more regions; two transcripts encoding 1-lysophosphatidylcholine acylhydrolase exhibited increased abundance in R2-R3 or R3, while two transcripts encoding phosphatidylcholine 2-acylhydrolases exhibited decreased abundance in R2-R3, a palmitoyl-[acyl-carrier-protein] hydrolase transcript was attenuated in R1, and a stearoyl-[acyl-carrier-protein] 9-desaturase was attenuated in R2-R3. Beyond these six transcripts, the majority were of low abundance but many showed small magnitude changes (below statistical significance) in region-specific patterns and WD did not appear to impart a consistent effect on any particular biosynthesis pathway. An acyl-CoA synthetase was the most abundant transcript, which was substantially enriched by WD in R2 and R3, but below the threshold of statistical significance used in this study.

## 4 Discussion

### 4.1 Complete maintenance of nodal root elongation during WD – contrasting responses to the primary root

The shoot-borne nodal root system in maize and other cereals comprises the majority of root length and, thus, provides the main pathway for water uptake in the mature plant (Ahmed et al., 2018). Accordingly, maintained growth of nodal root axes through dry upper soil layers is indispensable for drought avoidance and adaptation to arid climates (Uprety et al., 1981; Salih et al., 1999; Steinemann et al., 2015; Hazman and Brown, 2018). In this study, N2 nodal root axes exhibited complete maintenance of elongation during exposure to uniform and constant WD conditions that reduced the root tip (encompassing the growth zone) Ψ_W_ to -0.75 MPa (**Figures 1B, E**, **2A**). The maintenance of elongation in WD-treated nodal roots is congruent with a previous study by Westgate and Boyer (1985), who reported that elongation rates of approximately 1 mm h^-1^ were maintained until the growth zone Ψ_W_ fell to around -1.0 MPa, after which elongation progressively declined. While the WD treatment reduced the elongation of N1 relative to WW, the developmental programming of shoot-borne nodal roots (i.e., N2 and beyond) is at least partially distinct from N1, which develops from the coleoptilar node (Hochholdinger et al., 2004). Given this, it is likely that the growth maintenance exhibited by N2 and N3 roots in this study is characteristic of the later post-embryonic whorls of below-ground nodal roots.

Continued elongation of nodal roots during WD is predicated by maintenance of the growth zone, which encompasses both cell production in the meristem and cell expansion activities. Our kinematic analysis revealed that rates of both cell flux, which is indicative of cell production, and longitudinal expansion throughout the growth zone were fully maintained in the water-stressed roots; as a result, the length of the growth zone was maintained under WD conditions (**Figure 2B**). Furthermore, root diameter was slightly increased (**Figure 2C**) resulting from enhanced rates of radial plus tangential expansion (**Supplementary Figure S3C**). Consequentially, both the water content per unit length and volumetric expansion rate were slightly enhanced in the growth zone of N2 nodal roots under WD compared with WW conditions (**Figures 2D, E**).

These spatial growth characteristics of water-stressed N2 nodal roots contrast with those exhibited by the maize primary root under WD conditions. Although primary root elongation is partly maintained even under severe water stress (Sharp et al., 1988; Kang et al., 2022), elongation is nevertheless progressively inhibited over the range of soil Ψ_W_ in which nodal root elongation is fully maintained (Sharp et al., 1988). The inhibition of primary root elongation is associated with premature deceleration of longitudinal expansion and progressive shortening of the growth zone, with only the apical few mm (equivalent to R1 in the present study) maintaining relative elongation rates equal to those of WW roots (Sharp et al., 1988). This response has been consistently observed in primary roots of several genotypes, including FR697 as used in the present study (Kang et al., 2022). Cell production rate (Sacks et al., 1997) and root diameter (Sharp et al., 1988) were also substantially inhibited in water-stressed maize primary roots at levels of WD similar to the present study. Consequently, the overall rate of volumetric expansion in the primary root growth zone was shown to be reduced by almost 80% when growing at a soil Ψ_W_ of -0.8 MPa (Sharp et al., 1988), in stark contrast to the slightly increased rate of volumetric expansion exhibited in nodal roots growing under closely equivalent WD conditions.

### 4.2 Implications for the mechanism of osmotic adjustment in the nodal root growth zone

By enabling continued root growth and exploration of the soil for water, turgor maintenance by osmotic adjustment in roots is likely to be an important factor in maintaining crop yield under water-limited conditions (Sharp and Davies, 1979; Serraj and Sinclair, 2002; Voothuluru et al., 2024). Consistent with previous reports of maize nodal roots (Sharp and Davies, 1979; Westgate and Boyer, 1985), the N2 nodal root growth zone exhibited a high capacity for osmotic adjustment, resulting in full turgor maintenance under the imposed WD condition (**Figure 1C**). In the primary root, although the rate of tissue expansion is much more sensitive to water-stressed conditions than in the N2 growth zone, substantial osmotic adjustment is also exhibited (Sharp et al., 1990).

The differing morphogenic responses to WD within the nodal and primary root growth zones have important implications for the mechanism of osmotic adjustment in the two root types. To analyze this, it is important to recognize that increases in solute concentration can result from two distinct overall processes: a) increases in the net rate of solute deposition (net addition of solutes to the osmotic pool, encompassing uptake, import, local generation, utilization); and b) decreases in the net rate of water deposition (and, therefore, of solute dilution) that result from decreased rates of volumetric expansion (Sharp et al., 1990). In the maize primary root, Sharp et al. (1990) showed that osmotic adjustment in the basal regions of the growth zone (equivalent to R2 and R3 in the present study) could be quantitatively accounted for by reduced rates of water deposition that resulted from the inhibition of both longitudinal and radial expansion. In fact, net rates of osmoticum deposition were substantially decreased in these regions – increases in solute concentration nevertheless occurred because the rate of water deposition decreased to a greater extent. As a result, increases in solute concentration were attributable largely to decreases in water content rather than solute accumulation. Only in the apical region of elongation maintenance did the results provide evidence of increased solute deposition – specifically, the net rate of proline deposition increased by as much as 10-fold (Voetberg and Sharp, 1991), resulting in substantially increased proline contents per unit length of root (20-fold in the apical mm) compared with WW controls. In addition, decreased water deposition was again an important contributing factor due to the root thinning response.

In the water-stressed nodal root growth zone, in contrast, the maintenance of both root tip water content and rate of volumetric expansion indicate that rates of water deposition were maintained throughout the growth zone. Therefore, these results imply that the osmotic adjustment of the nodal root growth zone must have resulted from increased rates of net solute deposition, and increases in solute concentration were attributable to solute accumulation per unit root length throughout the growth zone rather than decreased water content. We therefore conclude that osmotic adjustment in the nodal root growth zone is likely an indispensable process for the complete maintenance of root growth under water-stressed conditions. Insights into the contributing metabolic pathways and accumulating solutes are provided by our metabolomic analysis, and are discussed in the following sections.

### 4.3 Regional patterns of osmolyte accumulation in the nodal root growth zone – comparison with the primary root

A subset of similar metabolic changes were observed in each region of the N2 root growth zone in response to WD. In all regions, a consistent set of metabolic pathways was identified as associated with those DAMs that exhibited altered abundances during WD (**Figures 3**, **4**; **Supplementary Figure S5**). In contrast, comparison of the metabolomes of the FR697 N2 nodal root and primary root (Kang et al., 2022) growth zones highlights several metabolites with distinct accumulation patterns under WD conditions (**Table 2**). Proline is one of the primary compatible solutes that accumulates in plant cells in response to osmotic stress (Yoshiba et al., 1997) and was among the highest fold-increase DAMs throughout the N2 root growth zone. As already mentioned, substantial proline accumulation also occurred in the growth zone of water-stressed primary roots (Voetberg and Sharp, 1991; Kang et al., 2022); in the apical 3 mm of the primary root, the fold-change increase was similar to that observed in R1 of N2 roots, but proline accumulation in the basal region of the primary root (which did not maintain elongation) was only approximately 25% of that of the comparable region in N2 roots. Similarly, sucrose content was also highest in the apical region of both WD-treated primary roots (Sharp et al., 1990; Kang et al., 2022) and nodal roots (**Table 2**); however, sucrose accumulation (compared to WW controls) throughout the N2 growth zone was nearly double that in comparable regions of the primary root.

**Table 2:** Statistically significant fold-changes of metabolite abundances in response to WD treatments in different regions of the growth zone in N2 nodal roots and the maize primary root.

Metabolite	N2 nodal root			Primary root (Kang et al., 2022)	
	R1 (WD/WW)	R2 (WD/WW)	R3 (WD/WW)	R1 (WD/WW)	R2 (WD/WW)
taurine	6.98	3.83	3.48	3	1.945
proline	18.6	17.87	19.25	16.34	4.6275
ornithine	1.06	1.95	1.83	0.665	1.0075
serine	1.61	1.68	1.74	2.58	1.82
tyrosine	0.65	0.79	0.89	0.915	0.5
arginine	0.86	0.82	0.87	1.465	1.345
citrulline	3.7	3.49	3.59	0.8	0.57
tryptophan	1.07	1.02	1.04	1.545	1.205
glutamate	1.04	1.24	1.36	0.705	1.0225
saccharopine	20.38	34.64	67.5	19.505	16.39
GABA	1.83	1.51	1.75	2.785	3.965
phenylalanine	0.65	0.72	0.76	0.92	0.6525
cysteine	5.59	7.01	8.11	1.7	1.175
methionine	1.04	1.04	1.26	0.77	0.31
sucrose	19.53	8.18	8.59	11.945	4.8775
glucose	1.13	1.29	1.4	1.765	1.345
fructose	1.59	1.84	2.3	2.61	2.3
raffinose	24.23	34	22.81	10.42	6.965
myo-inositol	3.71	5.57	6.91	1.45	1.7525
Reduced glutathione (GSSG)	5.91	6.28	8.6	6.395	1.9675
Oxidized glutathione (GSH)	1.58	8.72	25.02	41.375	5.57

Primary root data are from Kang et al. (2022), **Supplementary Data S1**. Primary root values are averages calculated as described by the authors. Red and green shaded cells indicate that the mean values are significantly higher or lower for that comparison, respectively (p ≤ 0.05). Light red and light green shaded cells indicate that the mean values trend higher or lower for that comparison, respectively (0.05< p< 0.10).

Increased proline accumulation during water stress can occur as the result of several factors including increased or decreased import, synthesis, catabolism, or utilization. Based on studies of metabolism of radiolabeled precursors, proline synthesis from glutamate and ornithine did not substantially increase in the apical region of the growth zone of water-stressed maize primary roots nor did catabolism decrease relative to synthesis, suggesting that enhanced translocation supplied the accumulated proline (Verslues and Sharp, 1999). While proline translocation was not evaluated in this study, transcriptomic abundances of key enzymatic steps in proline synthesis from glutamate and catabolism suggest that proline accumulation during WD may be at least partially accomplished through regulation of endogenous metabolic processes in the nodal root growth zone (**Figure 6**). In particular, we found evidence that proline synthesis may occur via lysine catabolism through the saccharopine pathway, which has the advantage of generating both proline and pipecolate that are both valuable for osmotic homeostasis (Arruda and Barreto, 2020).

Taurine accumulated in the growth zone of WD-stressed N2 nodal roots by approximately 3.5 to 7-fold compared with WW controls (**Figure 7**; **Table 2**). Taurine functions as both an organic osmolyte, accumulating during drought, salinity and freezing stresses, and in protection from oxidative stress (Yancey, 2005; Honjoh et al., 2007; Lambert et al., 2015; Tevatia et al., 2015; Rasheed et al., 2022; Ashraf et al., 2023). Taurine accumulation in the N2 growth zone during WD was approximately double that of the maize primary root grown under more severe water stress conditions (Ψ_W_ of -1.6 MPa) (**Table 2**; Kang et al., 2022).

WD-stressed N2 nodal roots exhibited altered arginine metabolism in a manner distinct from the primary root response. In every region of the N2 growth zone, arginine biosynthesis was among the top two significantly increasing metabolic pathways (**Figure 3**). However, arginine accumulation in the N2 growth zone decreased during WD, whereas primary roots of the same cultivar exhibited increased arginine content during WD (**Table 2**; **Supplementary Dataset 1**). Instead of increasing arginine accumulation as in the primary root, N2 roots increased in abundance of two precursors, ornithine and citrulline (**Table 2**; **Supplementary Figure S14A**).

In the basal region of the maize primary root growth zone, the majority of osmotic adjustment was attributable to increases in hexose concentration (Sharp et al., 1990). In the growth zone of water-stressed N2 roots, fructose and glucose accumulation increased during WD and the fold-change responses were similar to the levels reported in maize primary roots under WD conditions (Kang et al., 2022). However, as discussed above, N2 roots maintained the same water content in the growth zone during WD, whereas the primary root achieves increased solute concentrations largely by means of decreased water content (Sharp et al., 1990). Therefore, similar increases in hexose content in the primary root will achieve a lower Ψ_S_ (Sharp et al., 1990), suggesting that hexoses do not contribute as significantly to osmotic adjustment in the growth zone of nodal roots compared to the primary root.

### 4.4 Hormone signaling in the nodal root growth zone during WD

With regard to root growth regulation, ABA is of particular interest for the present study because it has been shown to be required for continued elongation of the maize primary root at low Ψ_W_ (Sharp et al., 1994). In this study, the WD treatment increased ABA content by 3 to 5-fold throughout the N2 nodal root growth zone. Interestingly, transcript profiling showed that ABA synthesis in the N2 root growth zone appeared, at least by transcript abundances, to be reduced during WD. It is possible that having effectively initiated the ABA signaling cascade (as evidenced by increased SnRK2 kinase transcripts in WD-treated roots), the ABA “signal” was fine-tuned in order to achieve homeostasis. Previous work suggests that roots do not synthesize sufficient ABA to sustain accumulation during WD stress, suggesting that stress induced ABA accumulation requires import from leaves (Ren et al., 2007). Additionally, reciprocal grafting experiments using labeled ABA demonstrated that, at least in the case of citrus and tomato, ABA accumulation in WD-stressed roots is largely transported from leaves (Manzi et al., 2015). It is important to note that the experiments in maize conducted by Ren et al. (2007) utilized young seedlings and, therefore, characterized ABA dynamics in the primary root, and it is possible that the mechanism of ABA accumulation in nodal roots is not reflected by these findings. However, if the majority of ABA accumulation in the nodal root is imported from leaves, then the WD-induced transcriptomic changes to ABA synthesis and catabolism we observed may be less relevant than changes in ABA perception and signal transduction.

Plant cells perceive ABA via receptors of the PYR-like (PYL) family. Upon ABA binding, these receptors interact with PP2C protein phosphatases, leaving the PP2Cs unable to dephosphorylate the SnRK2 kinases, which are then free to initiate a signaling cascade that includes phosphorylation targets such as transcription factors and membrane proteins (Umezawa et al., 2010; Soon et al., 2012). Transcriptomic profiling of this core signaling complex showed that WD resulted in increased transcript abundance for both PP2Cs and SnRK2 kinases, whereas abundance of PYL receptors was significantly reduced (**Supplementary Figure S8**). The dominant PYL receptor in the N2 root growth zone is annotated as ZmPYL7, and published whole plant RNAseq datasets suggest that this transcript is highly specific to nodal roots (Stelpflug et al., 2015; Walley et al., 2016). The stability and turnover rates of these ABA receptors is unknown, although some PYL receptors have been shown to be targeted by E2-E3 ubiquitin ligases for degradation (Lee et al., 2010).

In the maize primary root growing at low Ψ_W_, ABA acts to promote root elongation by restricting ethylene production and when ABA production is inhibited by chemical or genetic interference, ethylene production is greatly increased (Spollen et al., 2000). Application of exogenous ethylene reduces maize root elongation (Kim and Mulkey, 1997), and the root cap is required for ethylene-induced growth inhibition (Hahn et al., 2008). Transcript profiling and *in situ* hybridization experiments have previously demonstrated that the enzymes responsible for ethylene synthesis from its immediate precursor, ACC synthase (ACS) and ACC oxidase (ACO), are both present in the maize root cap and the basal region of the growth zone corresponding to R3 in our kinematic analysis. Maize mutants deficient in ethylene production exhibited higher cell production in the primary root tip meristem as well as longer cells, both of which were reversible with the application of exogenous ethylene (Gallie et al., 2009). The kinematic analysis of N2 nodal roots demonstrated that final cell length as well as cell production from the meristem were unaltered during WD, suggesting no ethylene-induced growth reduction or promotion in the N2 growth zone. The gaseous nature of ethylene requires specialized assays for its detection (Cristescu et al., 2012), hence ethylene was not assayed in our untargeted metabolome profile. However, neither metabolite abundance of the ethylene precursor S-adenosyl methionine nor transcript abundances of ACS or ACO suggested that ethylene production increased during WD (**Supplementary Figure S9**). Indeed, transcript abundances of ethylene responsive transcription factors were significantly attenuated during WD (**Supplementary Figure S11**).

WD stress has been shown to induce accumulation of indole acetic acid (IAA) in root apical regions, although divergent effects on growth have been reported. Increasing IAA concentration in the apical 5 mm of the maize primary root was correlated with decreased growth and evidence suggested that the accumulating auxin was likely synthesized locally in the root tip (Ribaut and Pilet, 1994). In contrast, IAA transport to the tip of rice and Arabidopsis primary roots was shown to activate the plasma membrane H^+^-ATPase during moderate water stress (Ψ_W_ of -0.47 MPa), thereby contributing to growth maintenance (Xu et al., 2013). In Arabidopsis, auxin transport is required for both the inhibitory effects on root growth induced by high ABA concentrations as well as the stimulatory effects of lower ABA concentrations (Li et al., 2017). Moreover, administration of exogenous auxin has been shown to partially rescue root growth of ABA-deficient tomato mutants (Zhang et al., 2022).

In the present study, IAA abundance was unchanged in R1 of WD-stressed N2 nodal roots, and increased 92% and 75% in R2 and R3, respectively, while transcript abundances of genes in the auxin biosynthetic pathway were not significantly changed (**Supplementary Dataset S2**; **Supplementary Figure S12A**). The effect of increased auxin on downstream signaling was assessed by querying transcript abundances of PIN effluxers, AUX/IAA repressors, ARFs and SAURs. The abundance of PIN1a was very slightly increased in abundance in R3 of WD-stressed roots, which exhibited the maximal increase in IAA accumulation during WD. The predominant IAA-related transcript belonged to IAA7, sometimes referred to as IAA10 in the literature (Wang et al., 2010, 2024), which was enriched in both R2 and R3 during WD (**Supplementary Figure S12B**). However, downstream auxin responsive elements did not exhibit a clear pattern reflecting either increased or decreased auxin signaling.

### 4.5 ROS homeostasis mechanisms in the nodal root growth zone are distinct from the primary root

Reactive oxygen species (ROS) accumulation during abiotic stress has been observed in many species. At high levels, ROS can impart oxidative damage to DNA and cellular structures (Sharma et al., 2012; Qiu et al., 2019). In addition, ROS are thought to play a key role in stress signaling, including adaptation to both biotic and abiotic stresses (Foyer and Noctor, 2005; Miller et al., 2010; Baxter et al., 2014; Ranjan et al., 2022). During WD stress, the maize primary root accumulates apoplastic ROS specifically in the apical region of the root growth zone that maintains elongation, but not in the basal region in which elongation is inhibited relative to WW roots (Voothuluru and Sharp, 2013). Apoplastic H_2_O_2_ imparts differing growth modulation effects to the maize primary root depending on whether growth is in WW conditions or under WD stress (Voothuluru et al., 2020). During growth in WW conditions, H_2_O_2_ acts to positively regulate cell production in the root apical meristem, thereby increasing root elongation; however, under WD conditions, apoplastic H_2_O_2_ acts to attenuate cell production leading to decreased root elongation (Voothuluru et al., 2020). The balance of ROS production and scavenging is altered spatially by WD in the growth zone of the maize primary root. Superoxide dismutase (SOD) proteins (which contribute to ROS production) were detected in the cell wall proteome in greater abundance in the apical region, while ascorbate peroxidases (APX, which scavenge ROS) were identified in lower abundances in the basal region (Zhu et al., 2007). Subsequent work showed that while WD increased H_2_O_2_ content in the primary root growth zone relative to WW plants, neither catalase (CAT), APX nor SOD activity increased concomitantly. Instead, glutathione levels increased specifically in the apical region of the FR697 primary root growth zone, suggesting that glutathione redox reactions are the primary means by which the maize primary root maintains ROS homeostasis during WD (Kang et al., 2022).

Similar to observations of the maize primary root, the N2 growth zone exhibited significant accumulation of both reduced (GSSG) and oxidized (GSH) glutathione under WD conditions (**Table 2**). While the primary root exhibited significant accumulation of GSH and GSSG only in R1, accumulation of both glutathione species was significantly increased throughout the entirety of the N2 growth zone. The changes in GSSG levels were similar to the primary root while GSH levels exhibited a more dramatic increase in the more basal regions, but only reaching approximately 60% of the increase exhibited in R1 of WD-stressed primary roots (when compared to the appropriate WW controls). Intriguingly, N2 roots appear to utilize a different strategy for employing glutathione in ROS homeostasis. Compared to the primary root, the redox balance of glutathione is reversed: while WD-stressed primary roots maintained a GSSG: GSH ratio of about 1:6.5 in the apical 3 mm, that ratio was roughly 3.75:1 in the comparable region of WD-stressed N2 roots, and then progressed to nearly 1:1 in R2 before reversing to 1:2.9 in the basal R3. The redox balance in the apical region may suggest that the glutathione pool has been exhausted, while a favorable balance is maintained in the more basal regions (Noctor and Foyer, 1998). Whereas the FR697 primary root only exhibited significant increases in glutathione accumulation (both reduced and oxidized species) in the apical region that maintained elongation during WD, the nodal root maintained elongation with much lower changes in reduced glutathione in the apical region (**Supplementary Dataset S2**). In the primary root, only glutathione peroxidase1 (Glpx1) exhibited significant change in transcript abundance (log2FC>1) (Kang et al., 2023), while Glpx1, Glpx3, Glpx6 and Glpx7 all increased significantly (log2FC>2) in R2 and R3, but not R1, of N2 roots (**Supplementary Figure S15A**). This increased transcript accumulation may be the cause of the increasing balance of GSH: GSSG in the distal parts of the growth zone. While the lack of transcript abundance changes associated with the glutathione cycle in the primary root suggest that glutathione-mediated ROS homeostasis is metabolically controlled (Kang et al., 2023), the significant changes in Glpx transcripts observed in N2 nodal roots during WD suggest a more coordinated regulation which might include transcriptomic adaptations as well as metabolic regulation.

### 4.6 Metabolic control dominates the WD response of the nodal root growth zone, although transcriptomic adjustments are observed for key pathways

The discordance between WD-associated changes in the transcriptome and metabolome (**Figure 5**) suggest that the majority of WD response is under metabolic rather than transcriptional control, as previously concluded for the maize primary root (Kang et al., 2023). Within the N2 nodal root growth zone, the transcriptome was best described by root region, rather than treatment (**Figure 5A**). The variance partitioned across root regions exceeded the variance partitioned across treatments to a substantial degree, suggesting that maturation within the growth zone, i.e., changes associated with the developmental progression of cells as they were displaced through the apical centimeter of the root, was the major determinant of transcriptomic-based programs within the root growth zone, rather than the responses to WD. Furthermore, developmental-related changes in transcriptomic based programs were markedly similar between roots grown under WW and WD conditions (**Supplementary Figure S7**). Although the metabolome exhibited a clear developmental effect with a R1 cluster clearly resolved from the R2 and R3 cluster (**Figure 5B**), the WD effect was more pronounced than the regional effect and more pronounced than the WD effect in the transcriptome PCA (**Figure 5A**). Thus, the nodal root transcriptome appears predominantly conditioned by maturation, whereas the metabolome appears primarily conditioned by the effect of WD stress.

For many significant metabolic responses to WD in the N2 root growth zone, transcriptomic adaptations were not observed despite significant changes in metabolite abundance (**Supplementary Figures S14**-**S16**). Discordance between mRNA abundance and protein abundance is well documented (Peng et al., 2015; Xing et al., 2018; Xu et al., 2021). A similar discordance between metabolomic and transcriptomic datasets, wherein only a fraction of the DAMs are present that would be predicted by enriched transcripts, was observed in the desiccation-tolerant grass *Sporobolus stapfanus* in response to dehydration as well as subsequent rehydration (Yobi et al., 2017, 2019). Given the discrepancy described by Yobi and colleagues, it is likely that the majority of DATs observed in the WD transcriptome in this study do not result in changes at the metabolite level. However, we identified several exceptions to this conclusion. Glutamate 5-kinase and glutamate-5-semialdehyde dehydrogenase, which catalyze the synthesis of the proline precursor glutamate 5-semialdehyde from glutamate, were significantly enriched by WD in the N2 root growth zone. In addition, transcripts encoding pipecolate oxidase, which serves to divert carbon flux from the saccharopine away from proline through a competitive pipecolate forming pathway, were strongly attenuated throughout the growth zone. Taken together, transcript and metabolite abundances suggest that the accumulation of proline in the N2 root growth zone during water stress is coordinated by the abundance of key enzymes that divert flux towards proline through the saccharopine pathway (**Figure 6**). R1 and R2 were also enriched in transcripts encoding the metabolic steps of hypotaurine and taurine synthesis from l-cysteate and the formation of 5-glutamyl-taurine from taurine (**Figure 7**). Concomitantly, l-cysteate accumulated at 1.4 to 3-fold higher in the N2 growth zone during WD while hypotaurine and taurine accumulated 3.4 to 6.9-fold higher. Thus, taurine and hypotaurine accumulation in the nodal root growth zone during WD appears to have a transcriptomic basis. Taken *in toto*, our data indicate that nodal root growth maintenance during WD stress encompasses a delicate combination of transcriptomic and metabolomic controls to coordinate the complexity of activities within the growth zone to facilitate continued root elongation.

## Data Availability

The datasets presented in this study can be found in online repositories. The names of the repository/repositories and accession number(s) can be found in the article/**Supplementary Material**.
