# Supplementary material for: Maize nodal root growth maintenance during water deficit: metabolic acclimation and the role of increased solute deposition in osmotic adjustment
Source: Front Plant Sci. 2025 Jun 9;16:1566453. doi: 10.3389/fpls.2025.1566453 (PMC12183189; doi:10.3389/fpls.2025.1566453)
Supplement: Supplementary file 6 [file DataSheet6.docx]

Supplemental Dataset 1-Metabolome Data

Supplemental Dataset 2- All Mapped Reads

Supplemental Dataset 3-Significant DATs With Annotation

Supplemental Dataset 4-Transcriptomic Changes Associated With Nodal Root Maturation

Supplemental Dataset 5-Transcription Factor Survey
