# Supplementary material for: Maize nodal root growth maintenance during water deficit: metabolic acclimation and the role of increased solute deposition in osmotic adjustment
Source: Front Plant Sci. 2025 Jun 9;16:1566453. doi: 10.3389/fpls.2025.1566453 (PMC12183189; doi:10.3389/fpls.2025.1566453)
Supplement: Supplementary file 10 [file Presentation1.pdf]

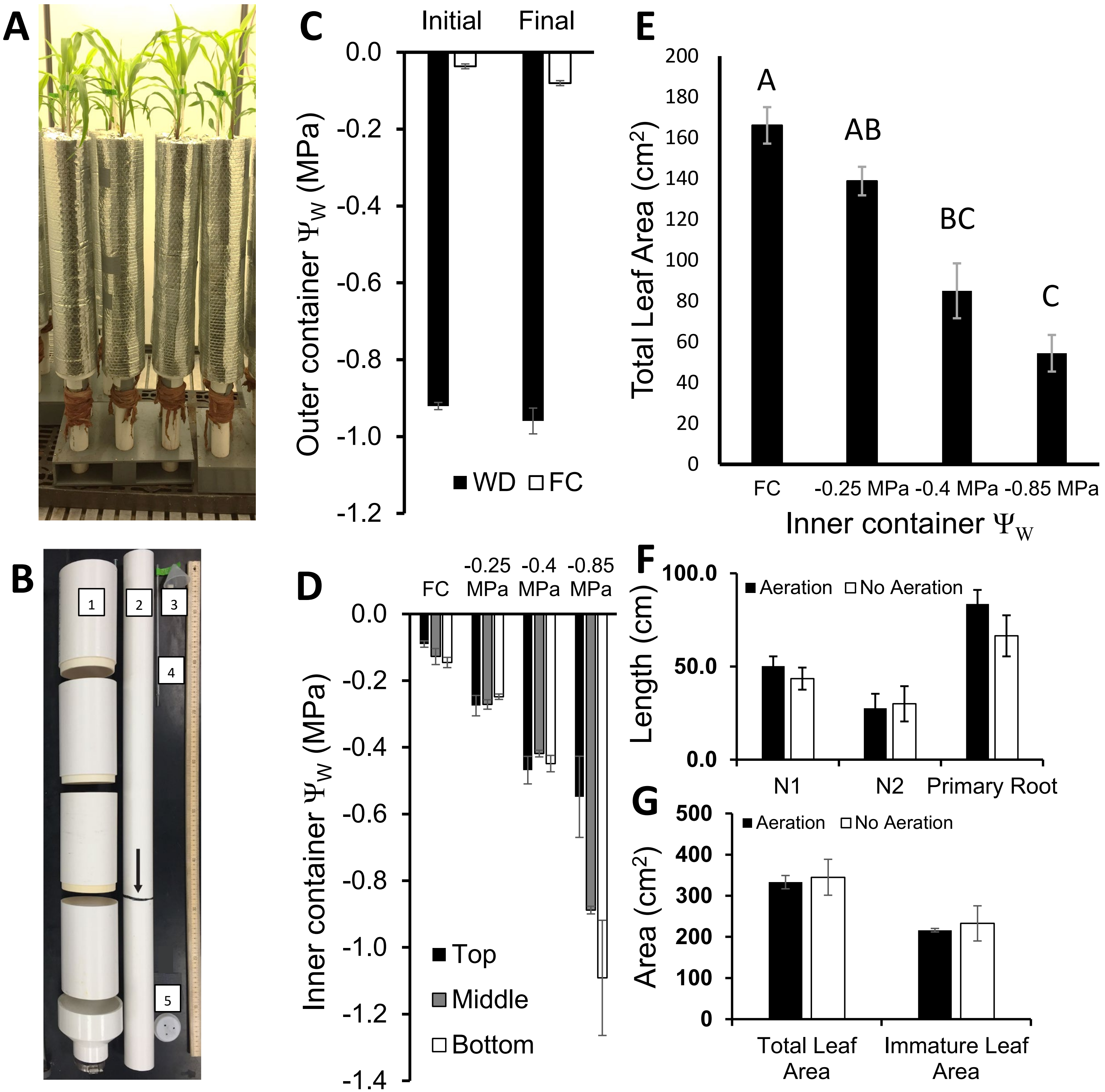

Supplementary Figure S1.

Supplementary Figure S1. (A) Plants growing in the divided-container root growth system. (B) The components of the divided-container root growth system: 1, separated sections of the PVC outer container; 2, PVC inner container; the arrow indicates the location of the bottom of the outer container when the system was assembled; 3, inner container cap; 4, watering tube; 5, inner container bottom plug with holes for drainage. (C) Outer container soil Ψ_W at the beginning and end of the 17-day growth periods for initial soil water treatments of FC or Ψ_W of -0.9 MPa (WD); the inner container contained FC soil in both treatments. For both treatments, the change in soil Ψ_W from the beginning to end of the growth period was not statistically significant (Student's *t*-test $P > 0.05$). (D) Vertical distribution of soil Ψ_W in the inner container after 15 days of growth with initial inner container soil water treatments of FC (transpired water replenished throughout the experiment) or Ψ_W of -0.25 MPa, -0.4 MPa, or -0.85 MPa; the outer container soil Ψ_W was -0.9 MPa in all treatments. (E) Leaf area after 15 days of growth for treatments with initial inner container soil water treatments of FC or Ψ_W of -0.25 MPa, -0.4 MPa, or -0.85 MPa; the outer container soil Ψ_W was -0.9 MPa in all treatments. Significance determined by one way ANOVA ($P < 0.05$); letters denote groups as determined by Tukey-Kramer post hoc analysis. (F) N1, N2 and primary root axial lengths and (G) leaf area for plants grown with FC soil in inner and outer containers with or without aeration of the inner container for 17 days. Data in (C-E) are means ± SE; n = 4 inner containers per treatment (C), 2-3 outer containers per treatment from each of 4 replicate experiments (D), 5-7 plants (E), 5 plants (F-G).

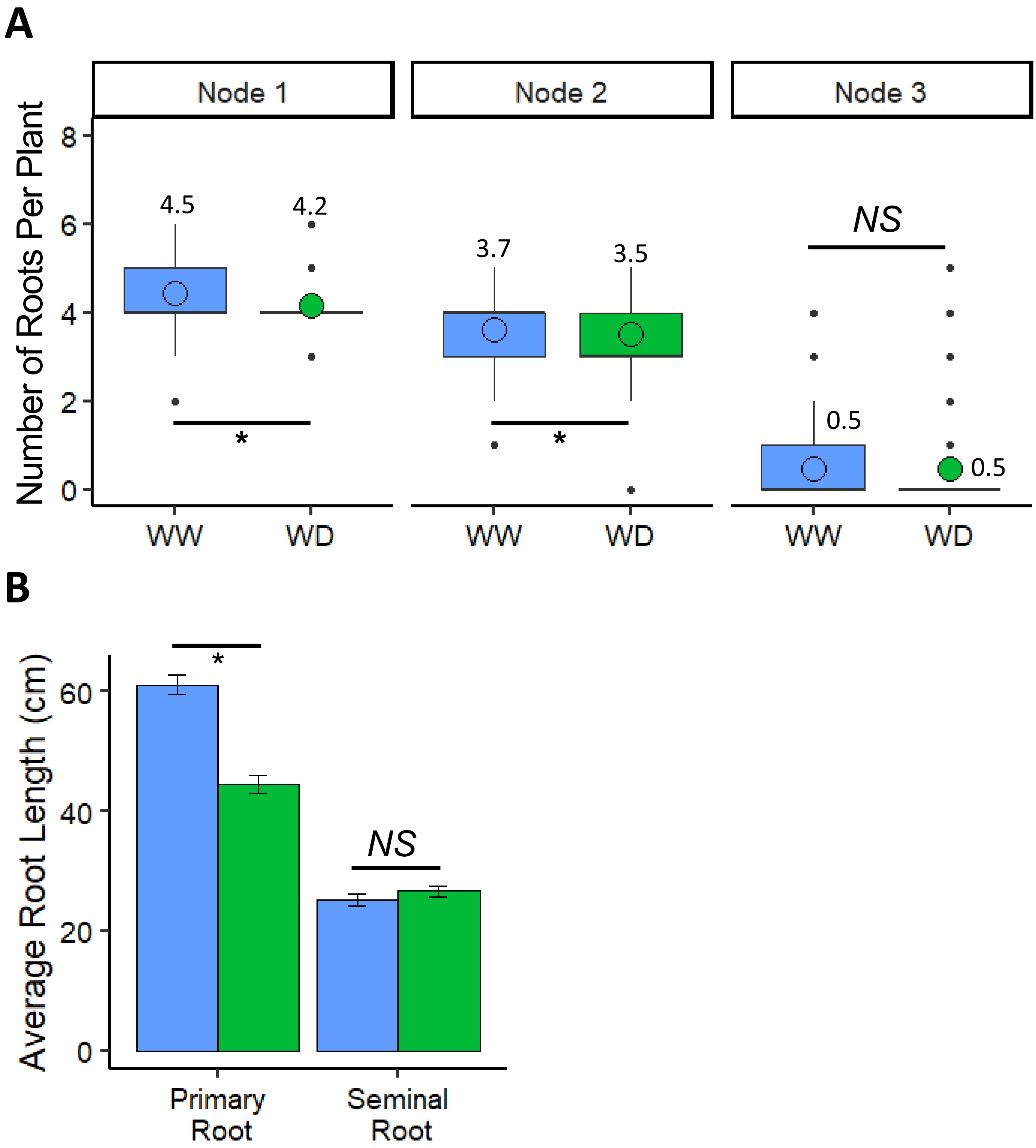

Supplementary Figure S2.

Supplementary Figure S2. (A) The average number of nodal (N1, N2, N3) roots per plant for WW and WD treatments. Mean values represented with open circles with values specified. (B) Average axial lengths per plant of primary and seminal roots for WW and WD treatments. Data are means ± SE; n = 146-149 plants. Significance calculated by Student's *t*-test, * $P \leq 0.001$, *NS* = not significant.

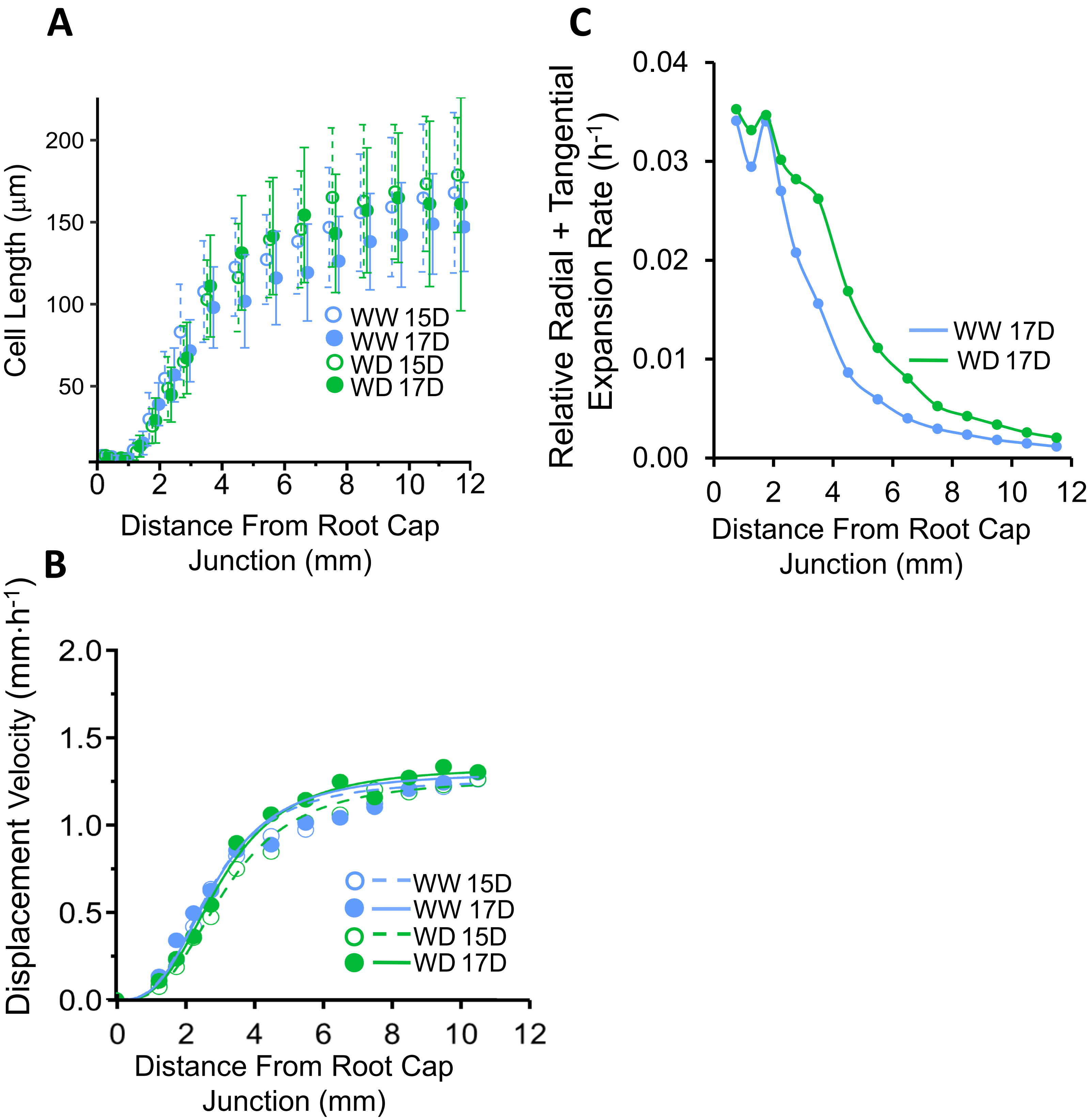

Supplementary Figure S3.

Supplementary Figure S3. (A) Cortical cell length profiles along the growth zone of N2 roots at 15 and 17 days after transplanting to WW and WD treatments. Data are means ± SD of 10 cells measured from each of 9 roots collected from 6-7 plants per treatment along each half millimeter increment from the root cap junction. (B) Displacement velocity profiles were calculated from root elongation rates (see Figure 2A) and mean cell lengths at each position. C) Radial plus tangential expansion rate profiles on day 17, calculated from spatial distributions of relative elongation rate (Figure 2B) and root diameter (Figure 2C).

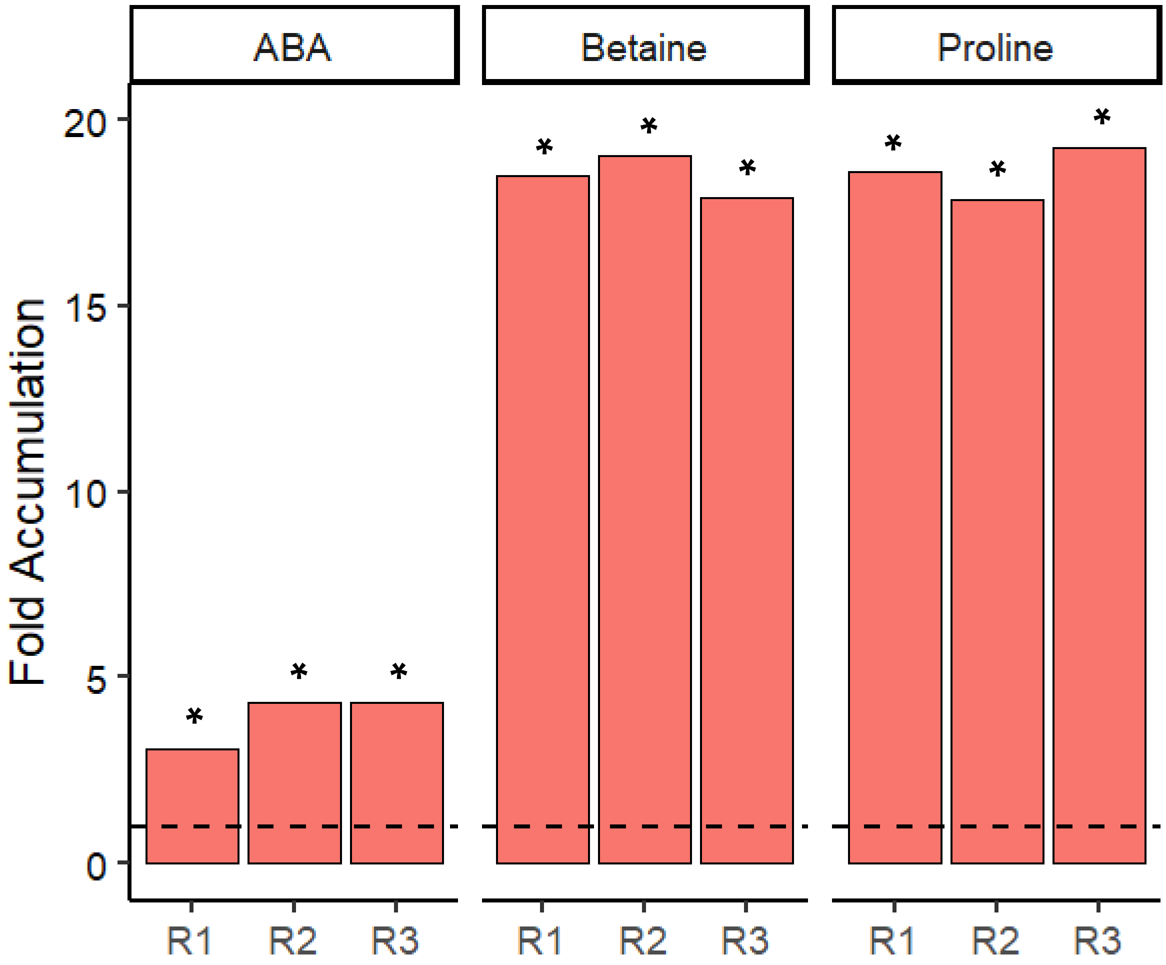

Supplementary Figure S4.

Supplementary Figure S4. Fold-change accumulation of ABA, betaine and proline in R1, R2 and R3 of N2 roots in the WD compared to the WW treatment. Dashed line indicates a fold accumulation value of 1 (no increase). Asterisks (*) denote FDR corrected statistical significance ($Q \leq 0.0001$) compared to mean WW values.

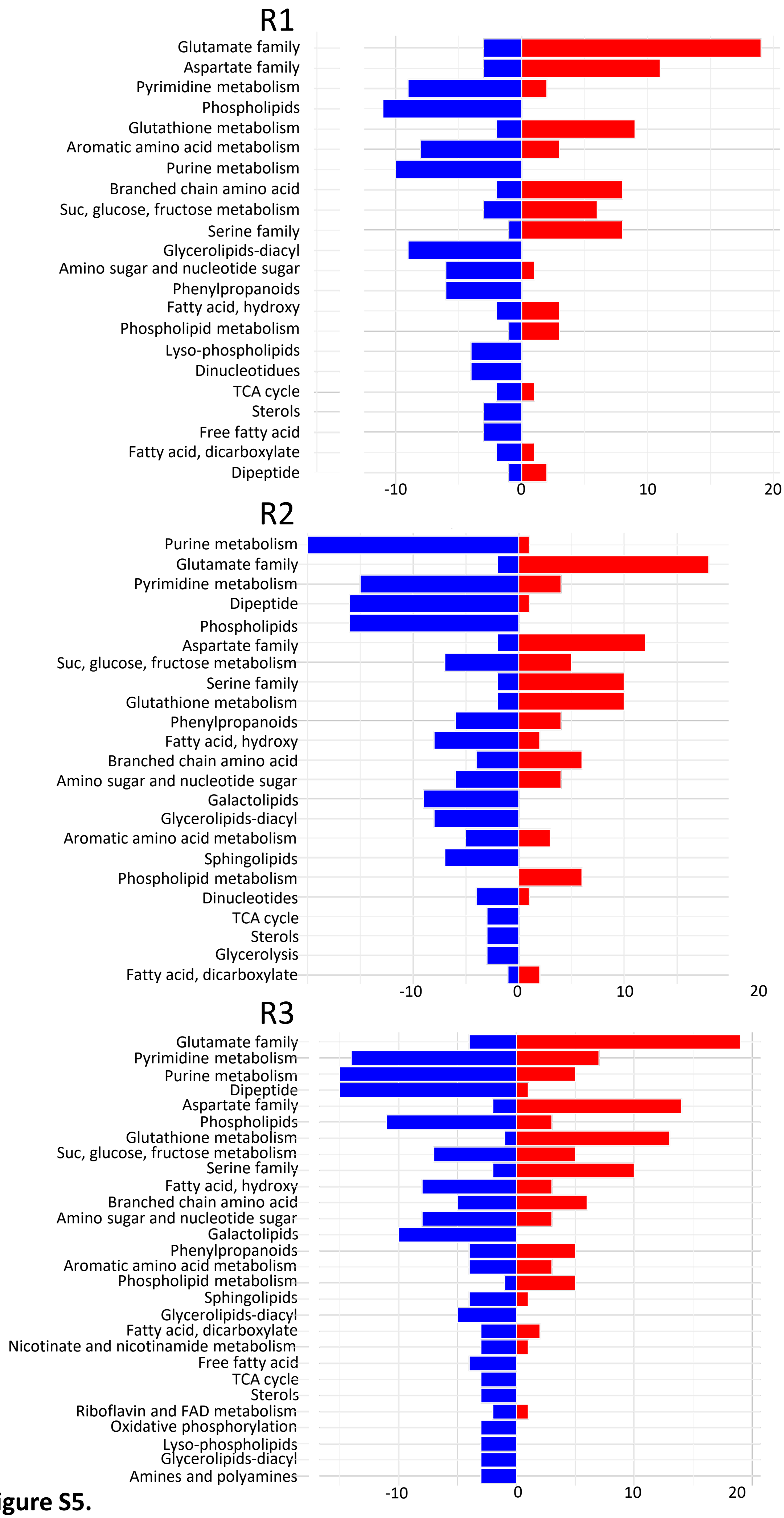

Supplementary Figure S5.

Supplementary Figure S5. Effect of the WD treatment on the metabolome in R1, R2 and R3 of N2 roots. Colored bars show the number of differentially accumulating metabolites (DAMs) within a given KEGG pathway. Blue denotes DAMs lower in WD compared to WW, red denotes DAMs higher in WD compared to WW.

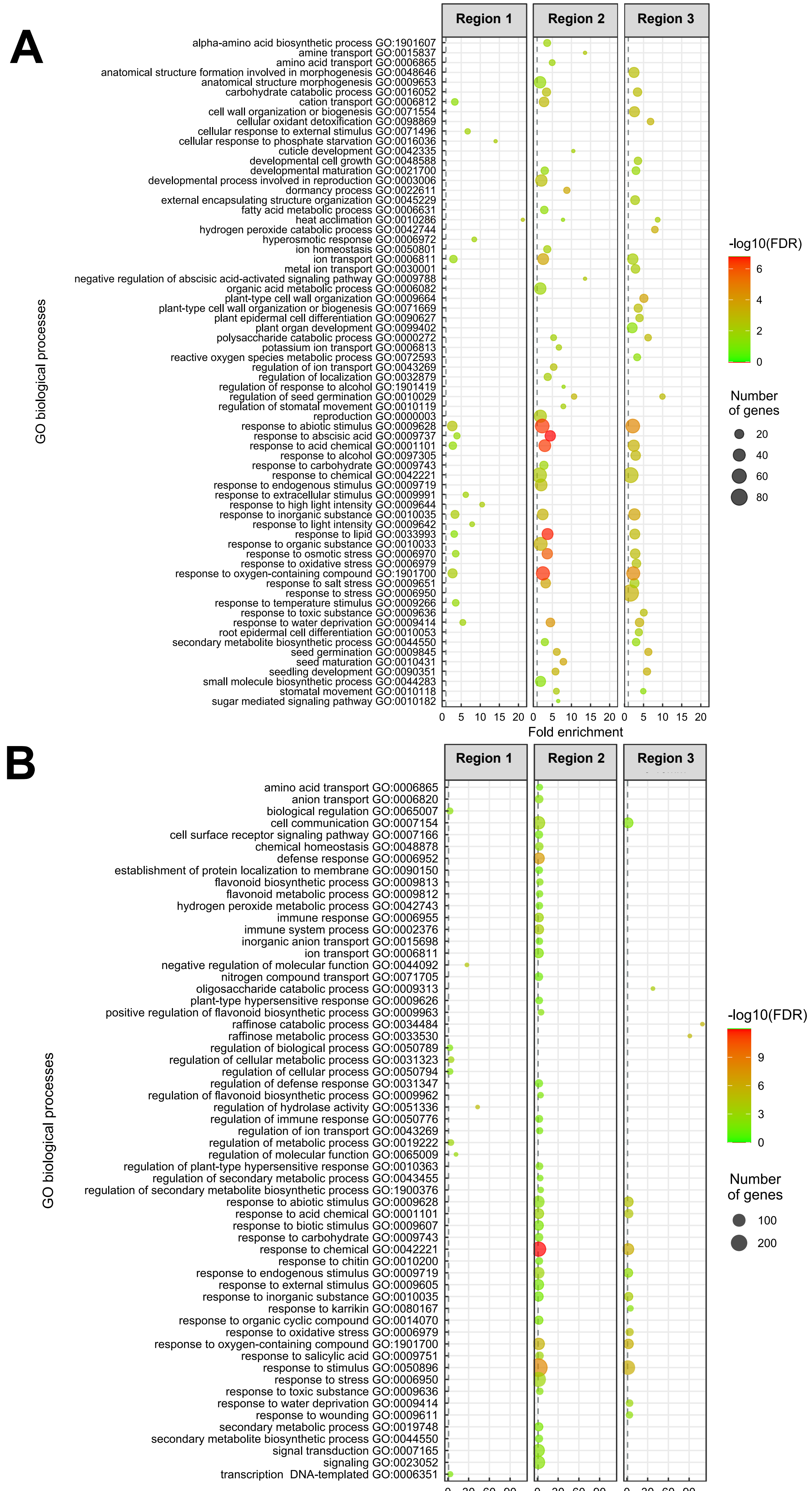

Supplementary Figure S6.

Supplementary Figure S6. Summary of enriched gene ontology (GO) categories describing highly WD-responsive transcripts ($log_2FC \geq 2$) in R1, R2 and R3 of N2 roots. (A) Transcripts that increased in abundance in WD compared to WW. (B) Transcripts that decreased in abundance in WD compared to WW. Larger bubbles denote more genes in this category, color denotes significance by FDR corrected *p*-values (Fischer's exact test).

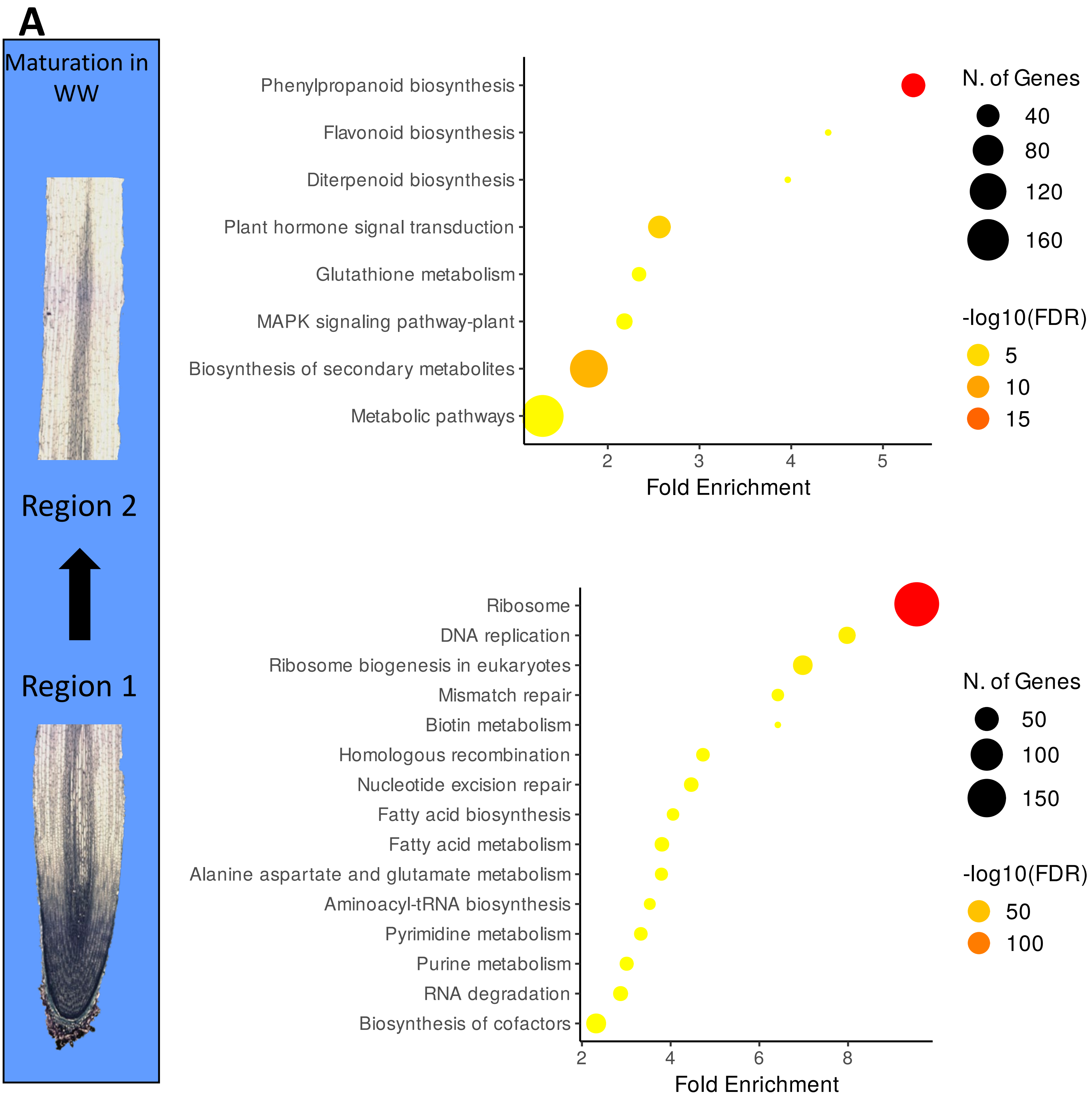

Supplementary Figure S7.

B

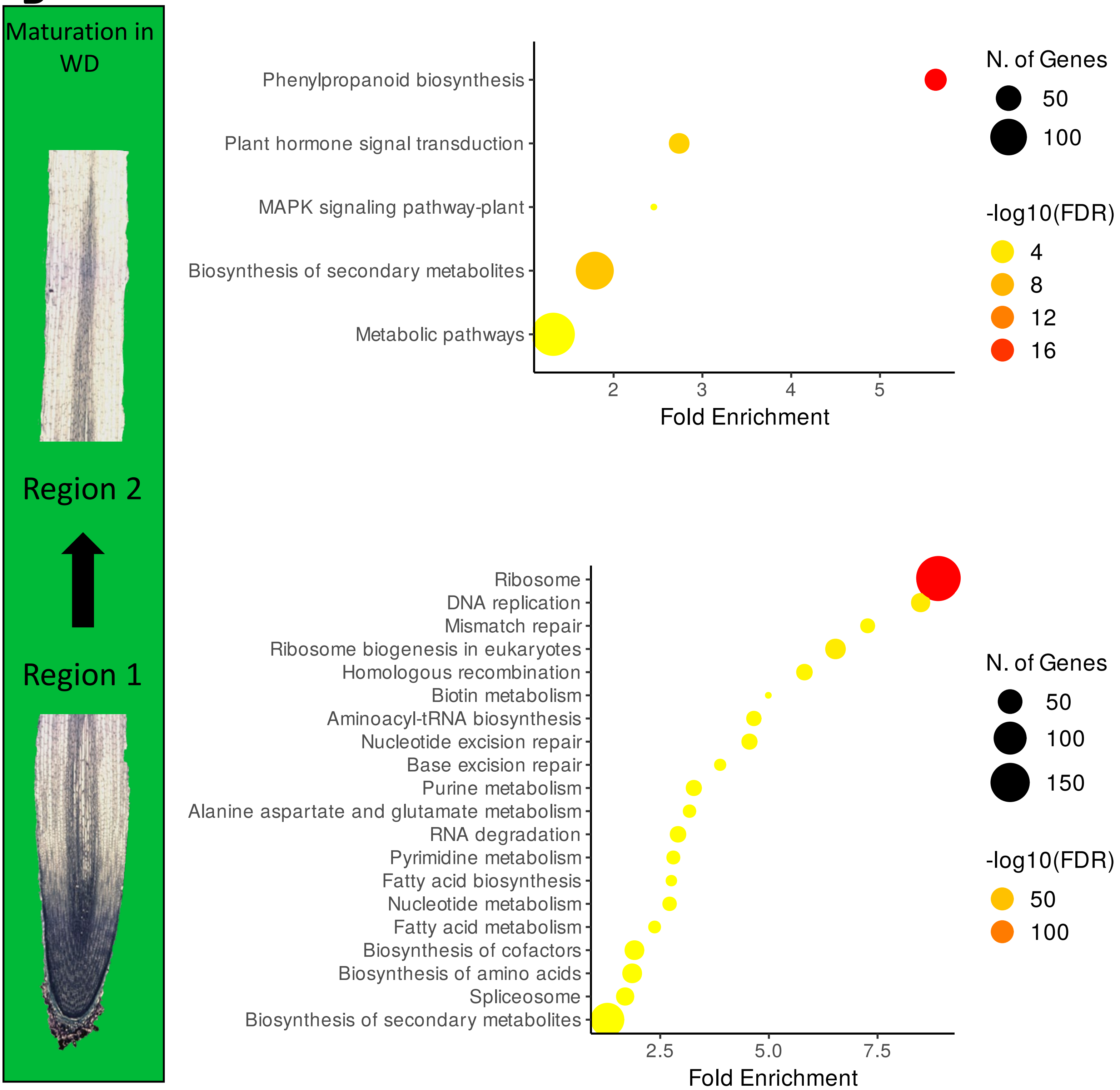

Supplementary Figure S7.

Supplementary Figure S7. Summary of transcriptomic changes associated with the transition from R1 to R2 in WW N2 roots (A) and WD N2 roots (B). Lower plot represents pathways enriched in R1 compared with R2, upper plot represents pathways enriched in R2 compared with R1. KEGG functional enrichment analysis was performed for transcripts with significant ($log_2FC \geq |2|$) differences between the two regions. Larger bubbles denote more genes in this category, color denotes significance by FDR corrected P-values.

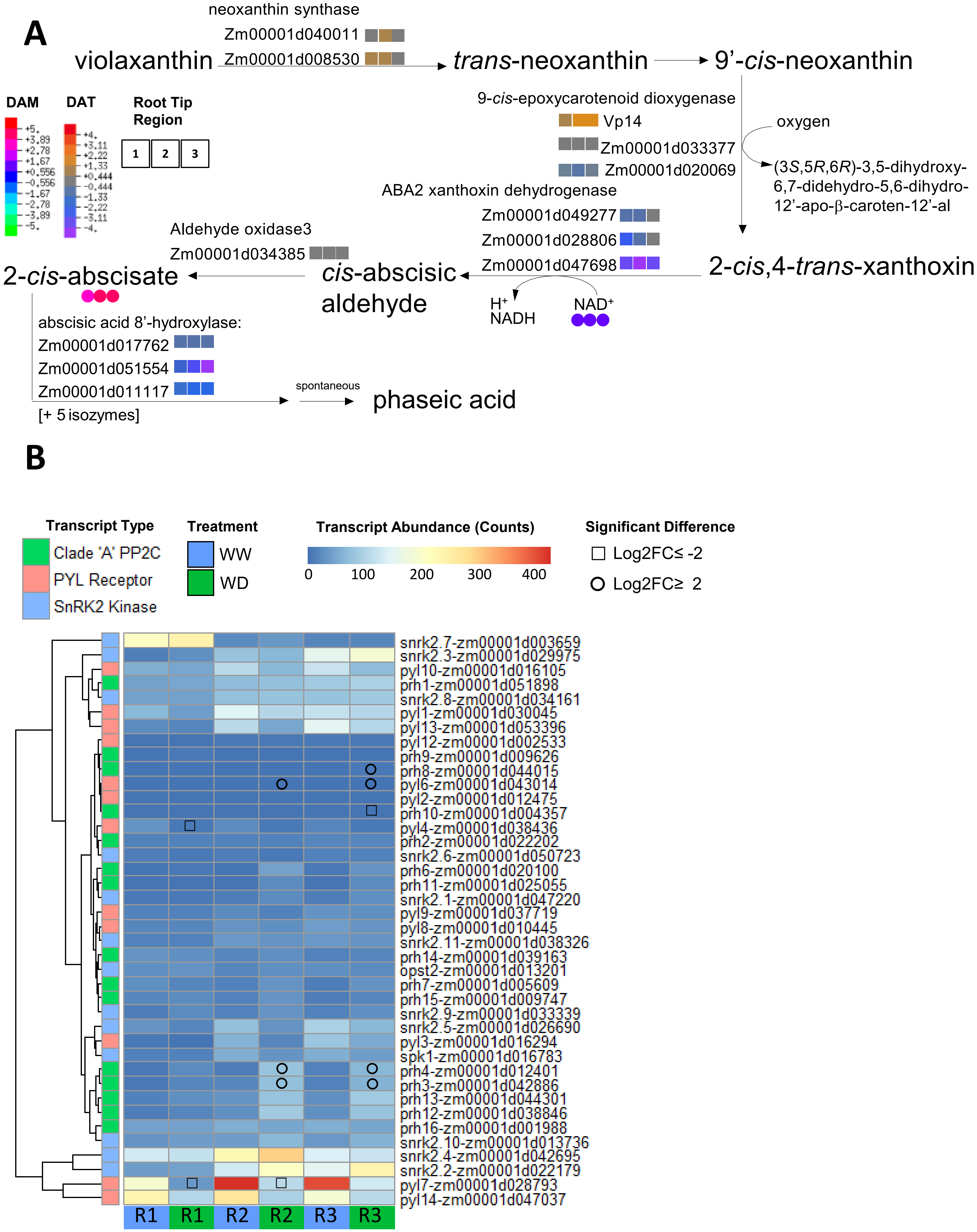

Supplementary Figure S8.

Supplementary Figure S8. The effect of WD on ABA perception and signaling in the N2 root growth zone. (A) ABA accumulation increases during WD but transcripts encoding the biosynthetic steps are not uniformly enriched, while transcripts encoding ABA degradation isozymes are significantly attenuated. Colored circles indicate DAM fold-change (value >1 indicates enrichment in WD compared to WW); colored squares indicate differentially accumulating transcript (DAT) $\log_2$ fold-change (positive value indicates enrichment in WD relative to WW) in R1, R2 or R3 (left to right, respectively). (B) Transcript abundances of genes in the PYL receptor- PP2C- SnRK2 signaling pathway. Colored rectangles denote mean normalized transcript abundances (counts). Squares and circles in heatmap cells indicate significantly different transcript abundance (log2 FC $\leq$ -2 or $\geq$ 2, respectively) between treatments. Root regions indicated at bottom of heatmap.

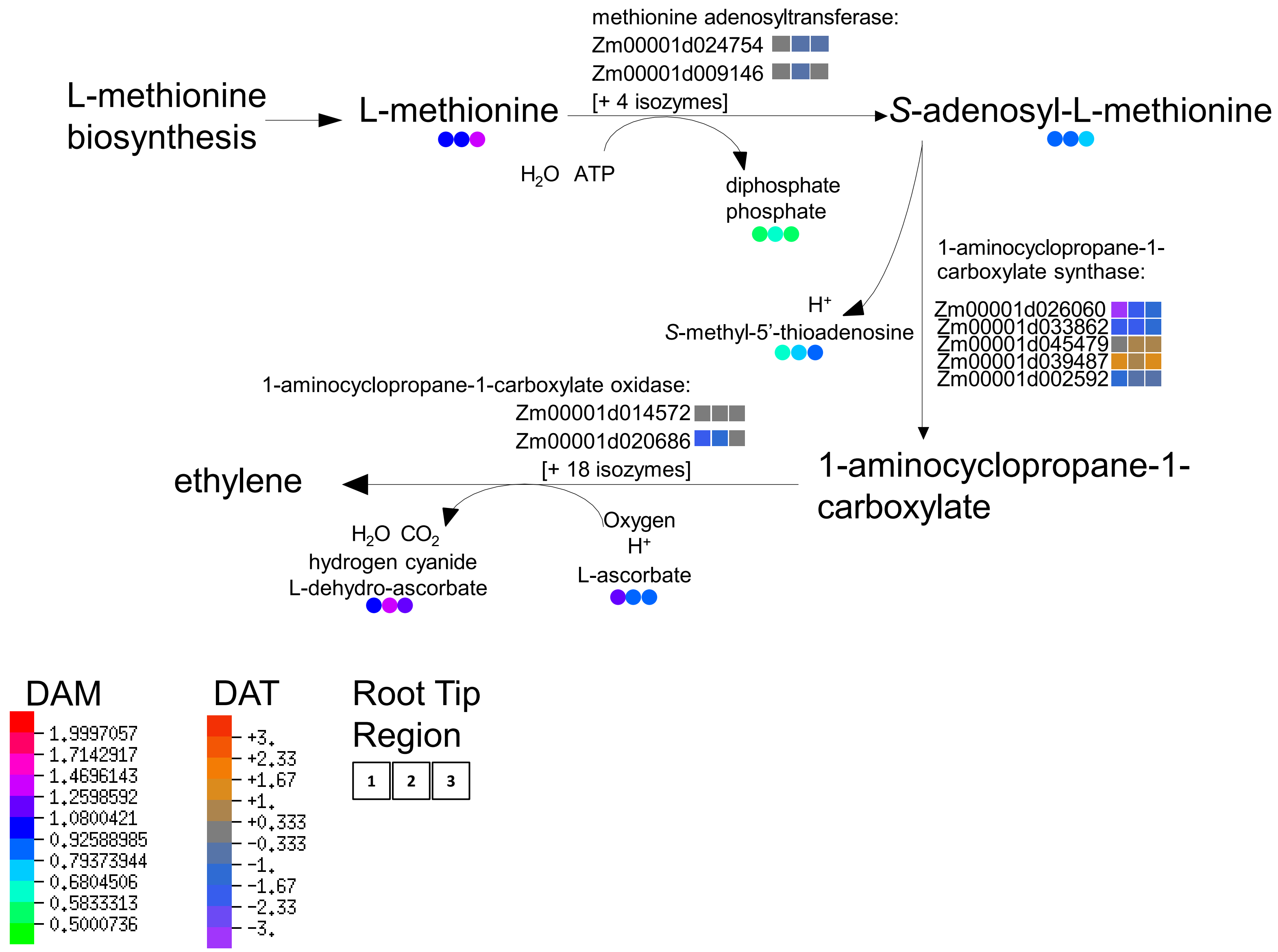

Supplementary Figure S9.

Supplementary Figure S9. Effect of WD on abundances of transcripts encoding ethylene biosynthesis-related genes and associated metabolites in the N2 root growth zone. Colored circles indicate metabolite fold-change (value >1 indicates enrichment in WD compared to WW); colored squares indicate $\log_2$ fold-change between transcript abundance of WD and WW (positive value indicates enrichment in WD relative to WW) in R1, R2 or R3 (left to right, respectively).

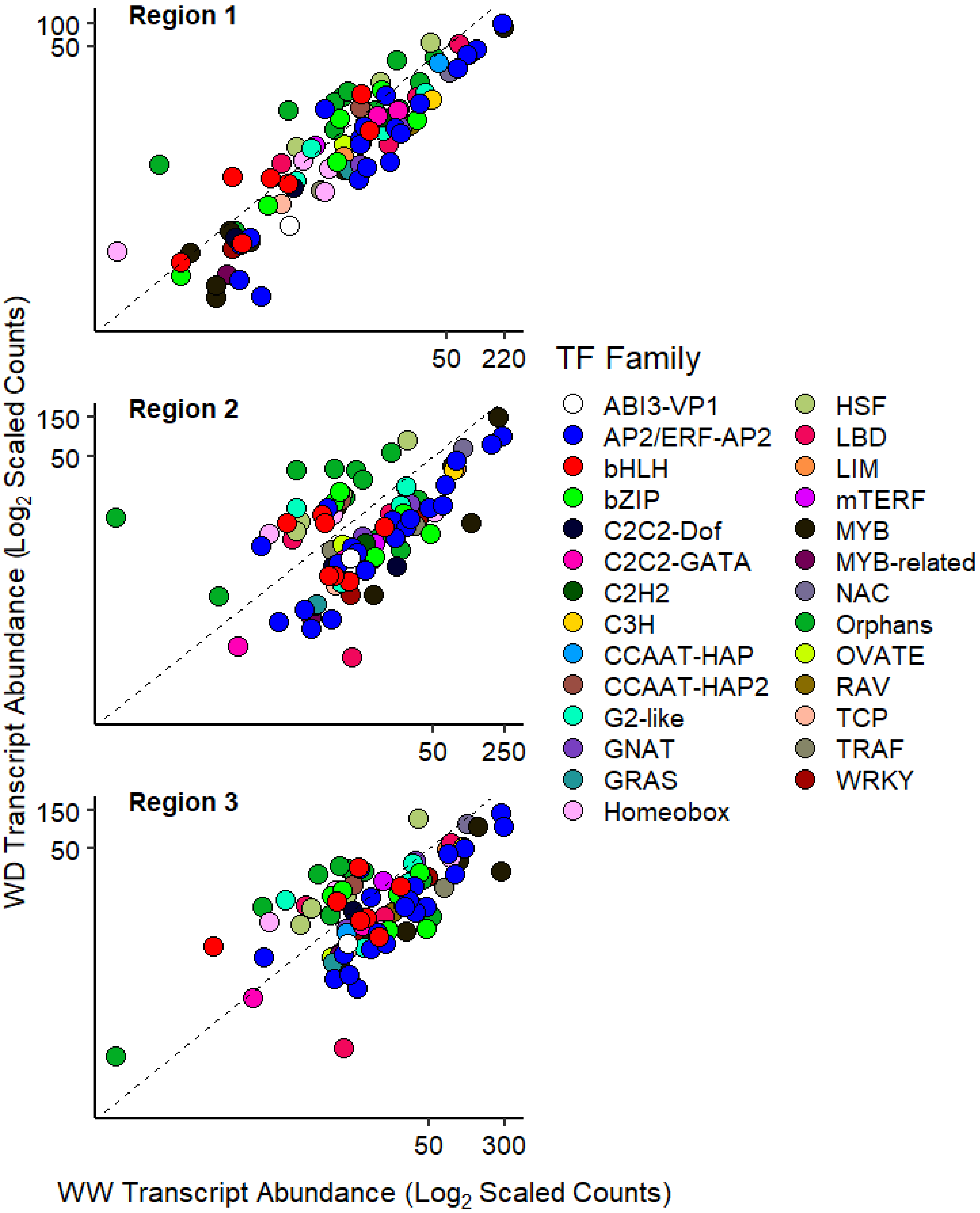

Supplementary Figure S10.

Supplementary Figure S10. Transcript abundances of transcription factors that were significantly altered ($log_2FC \geq |1.2|$) by WD in R1, R2 and R3 of the N2 root growth zone. Dashed line indicates a 1:1 ratio of transcript abundance, i.e. equivalent abundance in WD and WW. X and Y axes are both log_2 scaled.

A

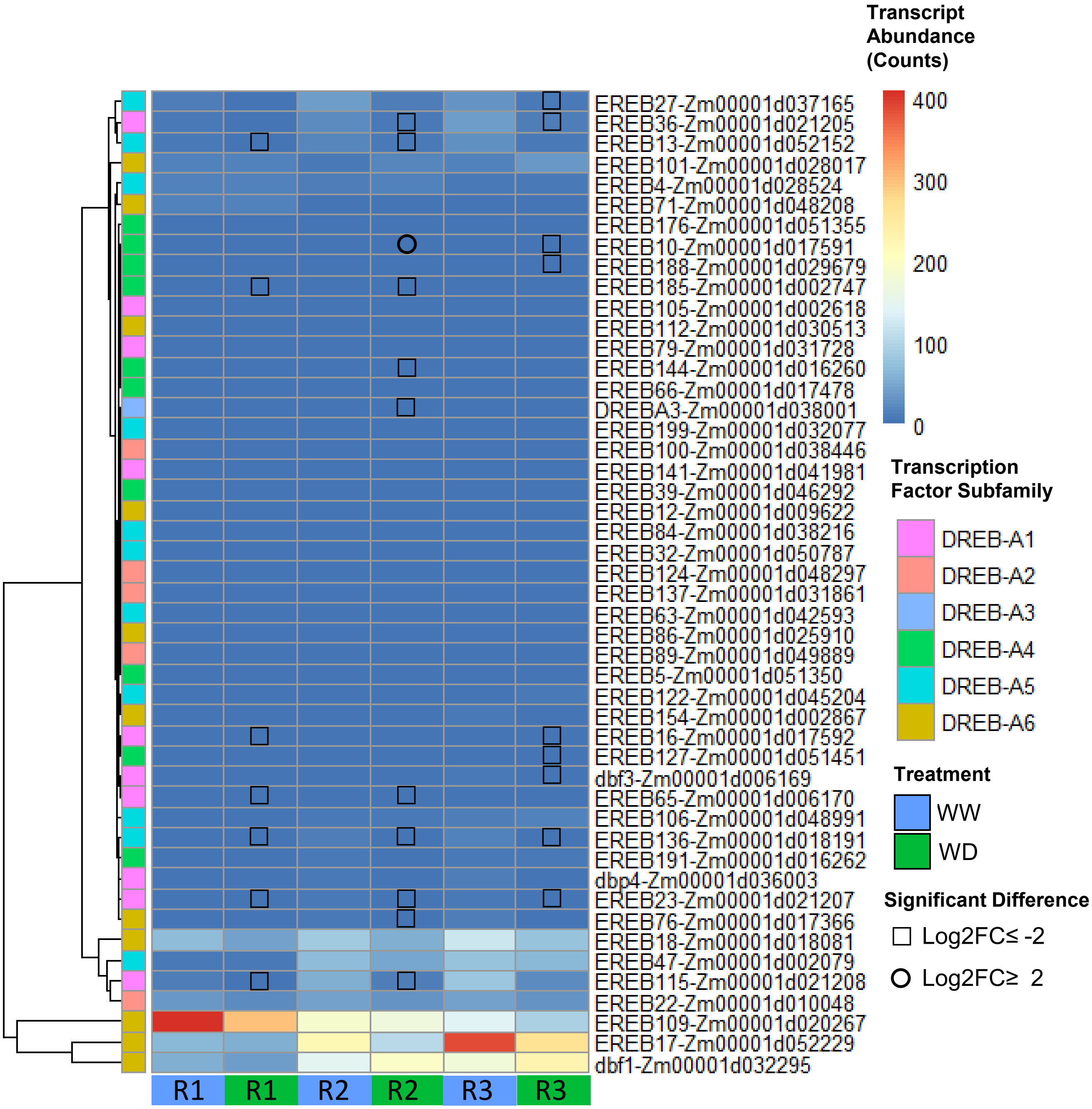

Supplementary Figure S11.

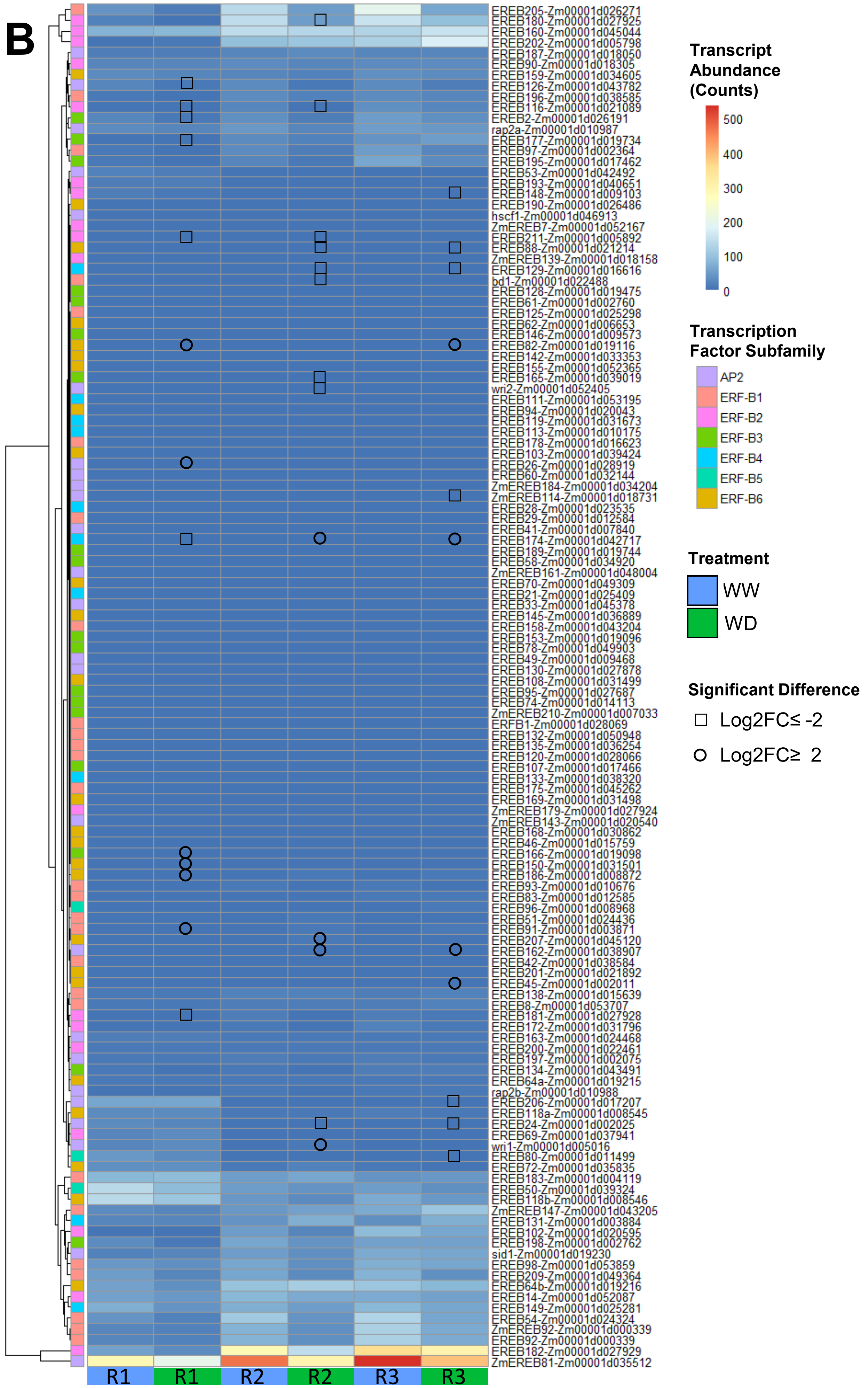

Supplementary Figure S11.

Supplementary Figure S11. Transcript abundances of ethylene-regulated transcription factors in R1, R2 and R3 of the N2 root growth zone in WW and WD treatments. A) DREB- type, B) ERF and AP2-types of ethylene response factors. Colored rectangles denote mean normalized transcript abundances (counts). Squares and circles in heatmap cells indicate significantly different transcript abundance (log2 FC $\leq$ -2 or $\geq$ 2, respectively) between treatments. Root regions indicated at bottom of heatmap.

A

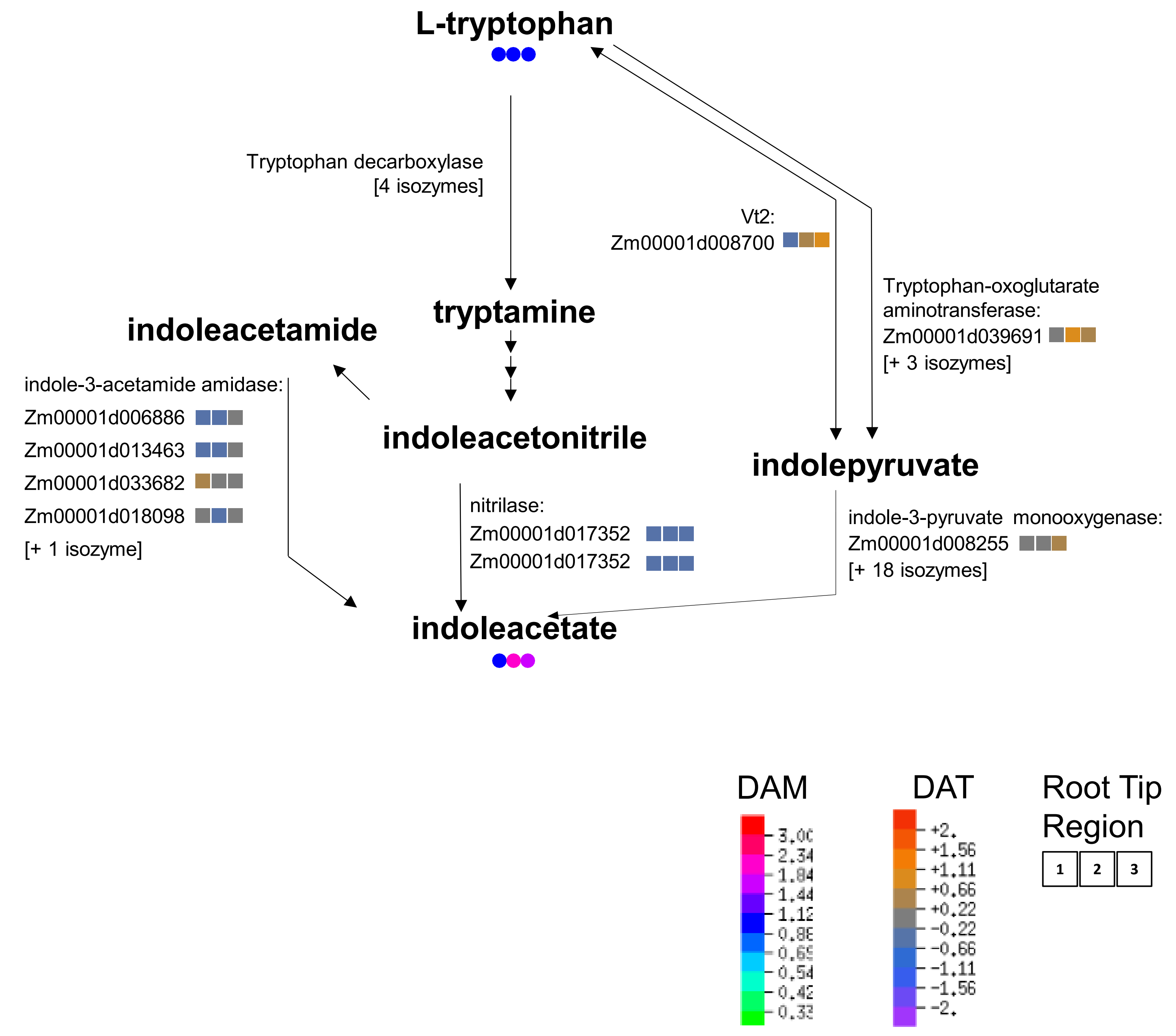

Supplementary Figure S12.

B

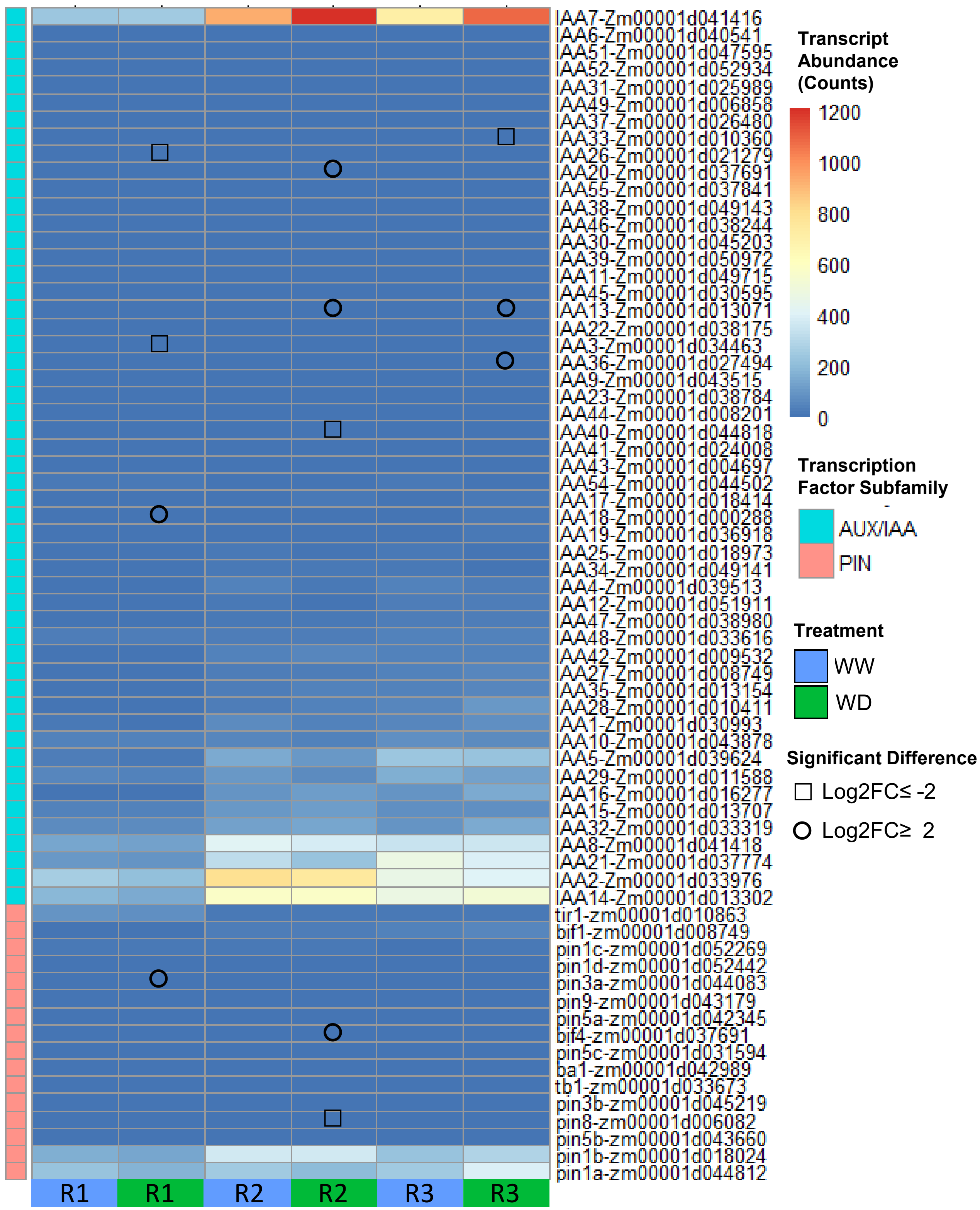

Supplementary Figure S12.

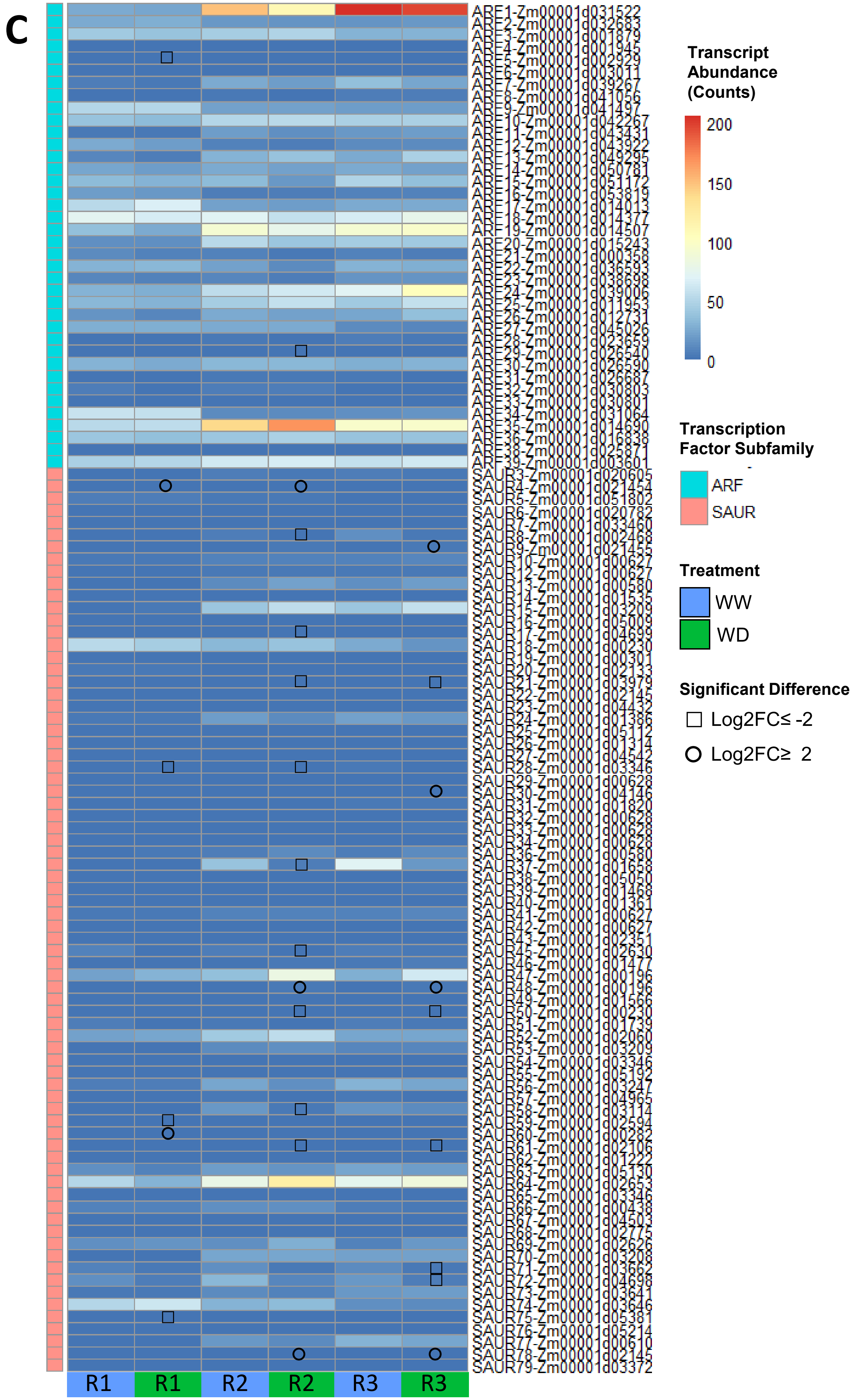

Supplementary Figure S12.

Supplementary Figure S12. Effect of WD on auxin perception and signaling in R1, R2 and R3 of the N2 root growth zone in WW and WD treatments. (A) Abundances of transcripts and metabolites associated with the auxin biosynthesis pathway. Colored circles indicate metabolite fold-change (value >1 indicates enrichment in WD compared to WW); colored squares indicate $\log_2$ fold-change between transcript abundance of WD and WW (positive value indicates enrichment in WD relative to WW) in R1, R2 or R3 (left to right, respectively). (B) Abundances of transcripts encoding PIN efflux carriers and AUX/IAA repressors; (C) abundances of auxin response factor (ARF) and small auxin upregulated RNA (SAUR) transcripts. Colored rectangles denote mean normalized transcript abundances (counts). Squares and circles in heatmap cells indicate significantly different transcript abundance (log2 FC $\leq$ -2 or $\geq$ 2, respectively) between treatments. Root regions indicated at bottom of heatmaps.

A

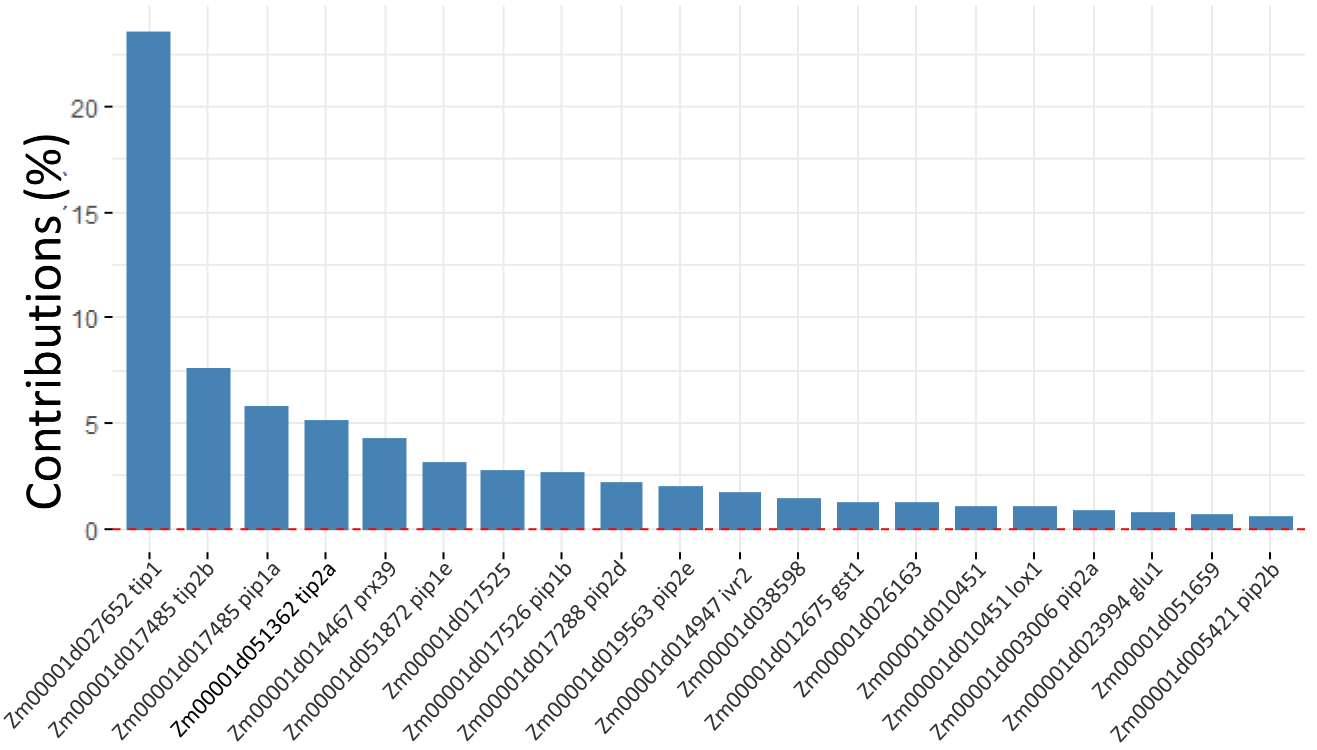

B

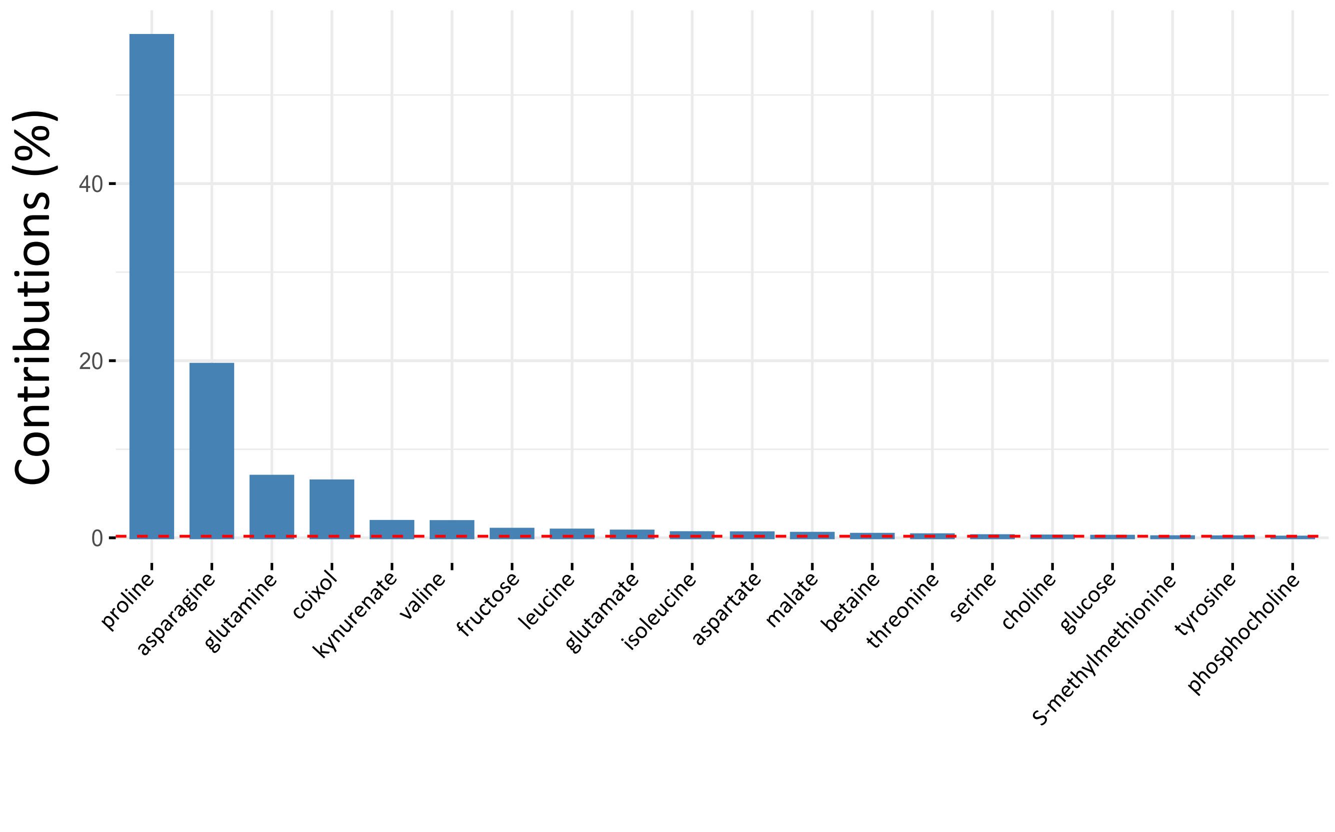

Supplementary Figure S13.

Supplementary Figure S13. Contribution of the 20 most significant transcripts (A) or metabolites (B) to loadings of PC1 and PC2 for respective principal components analyses. Dashed red line indicates expected contribution towards total variance if distributed equally (i.e. 100/ total number of transcripts or metabolites).

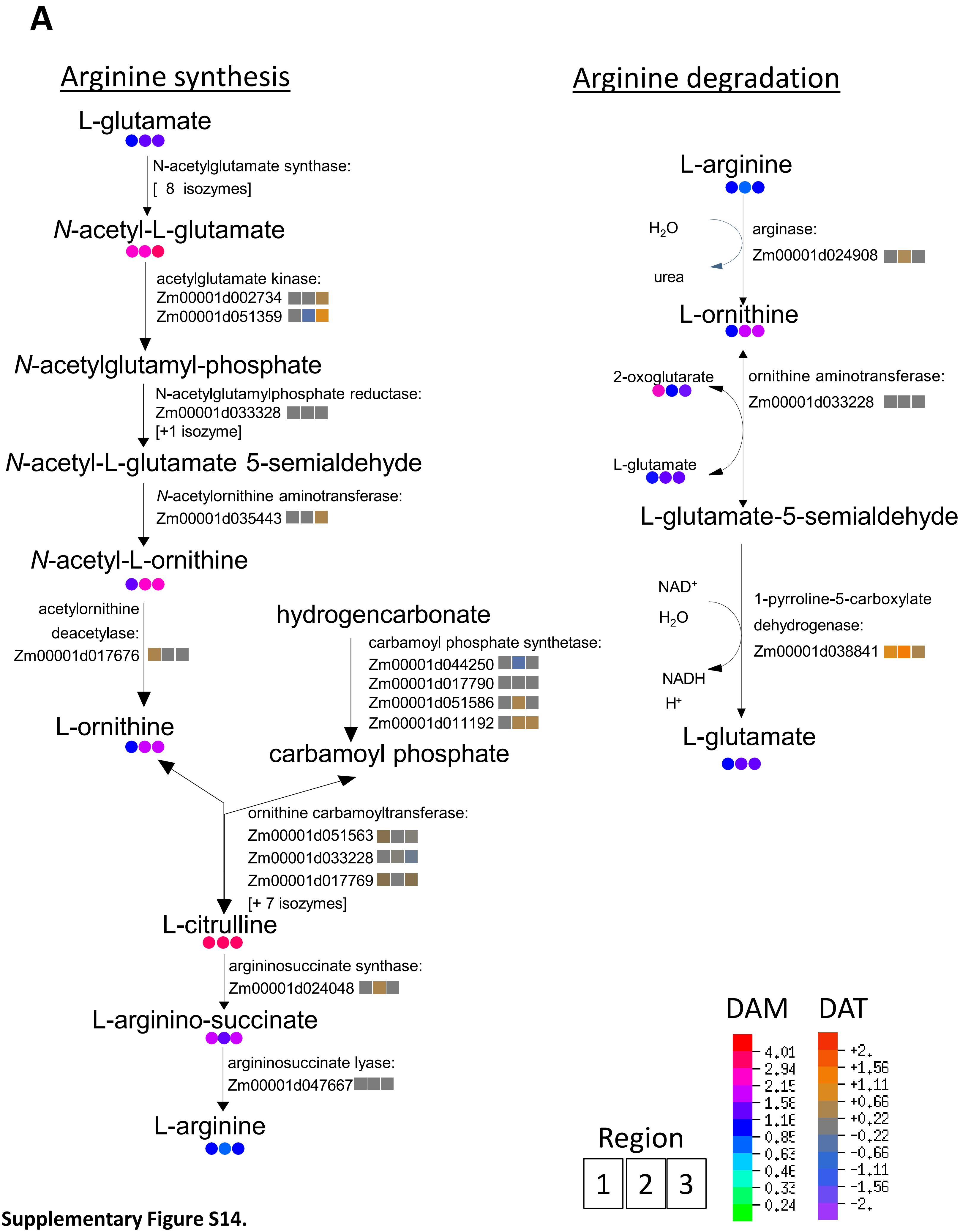

Supplementary Figure S14.

B

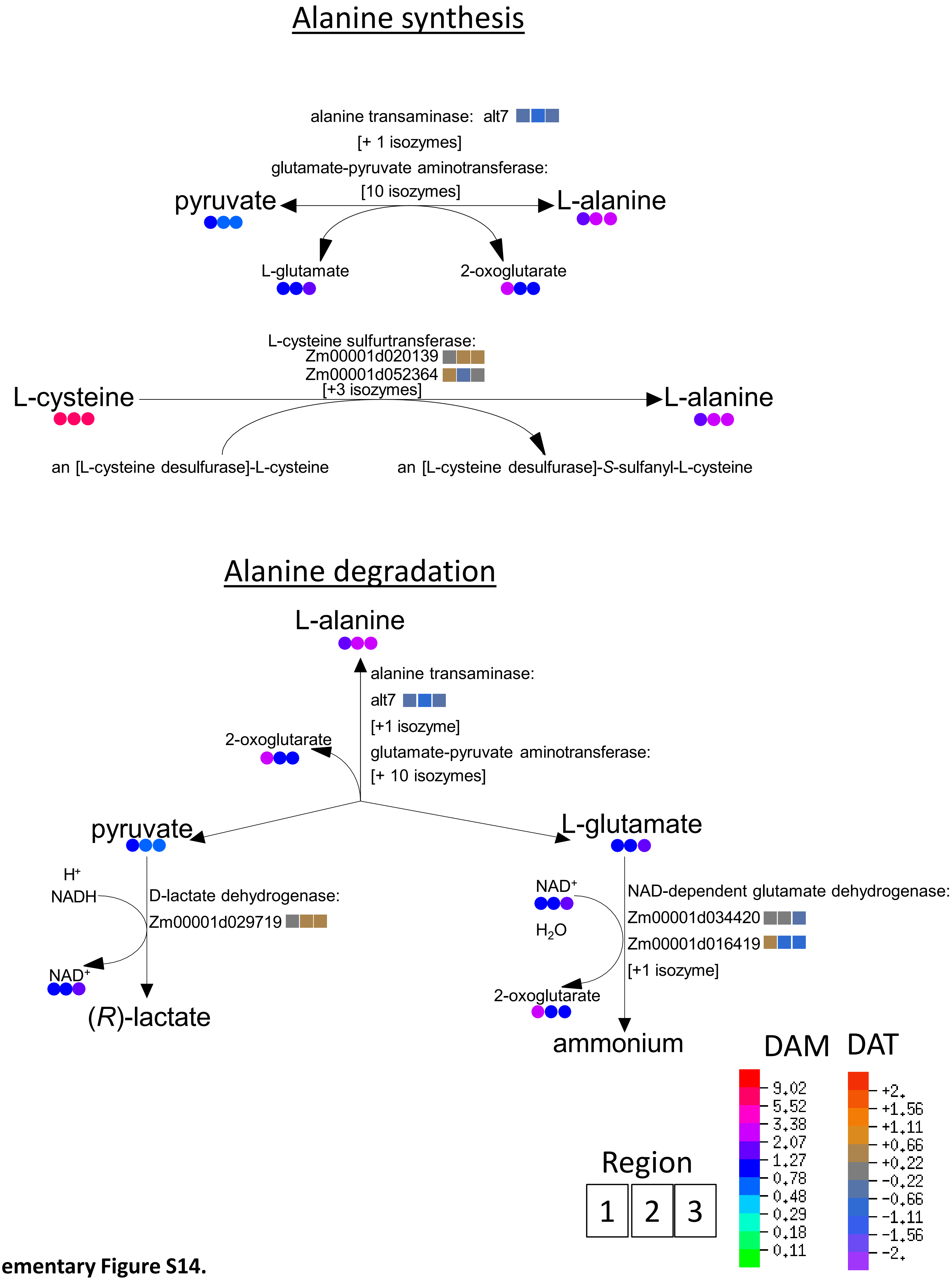

Supplementary Figure S14.

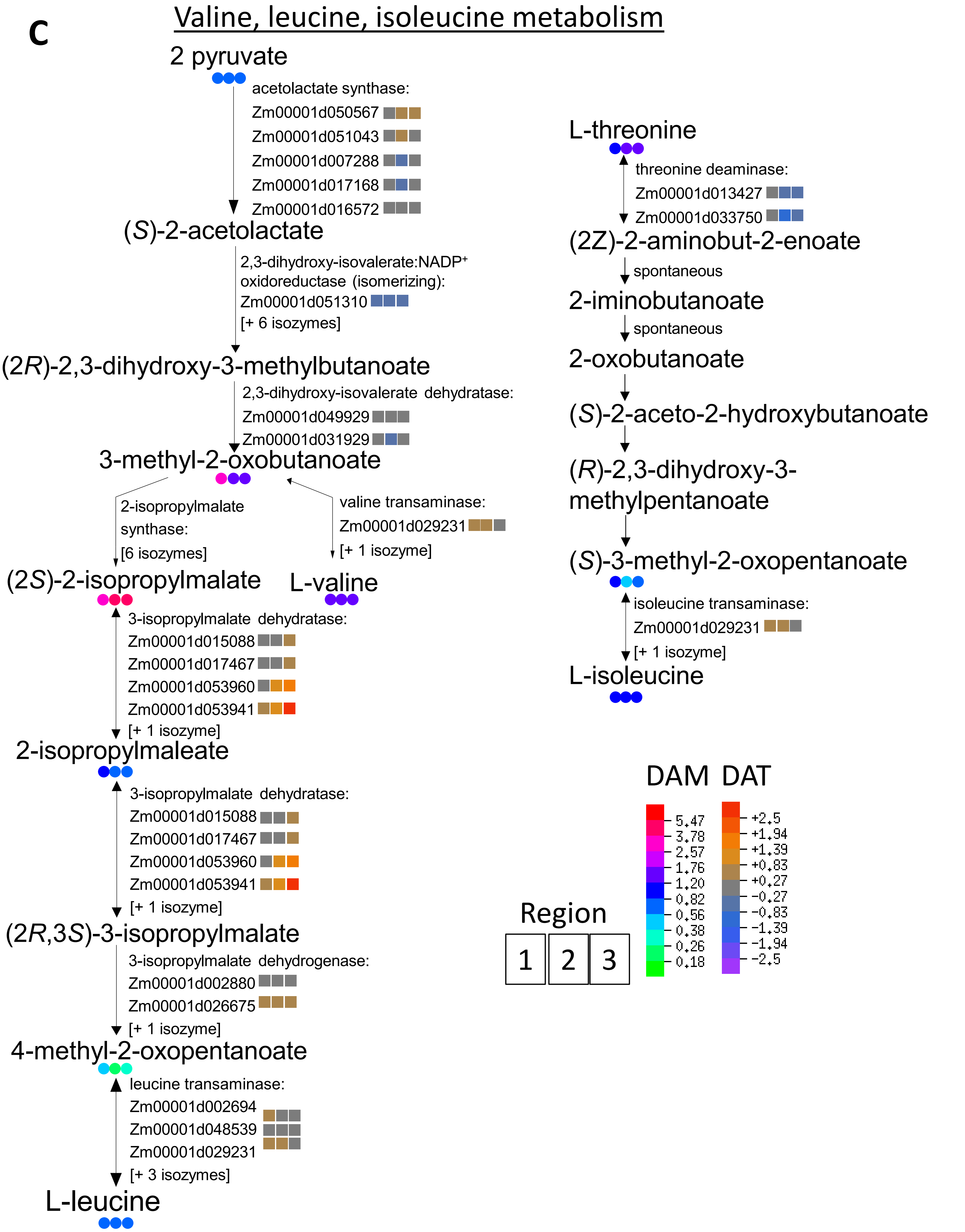

Supplementary Figure S14.

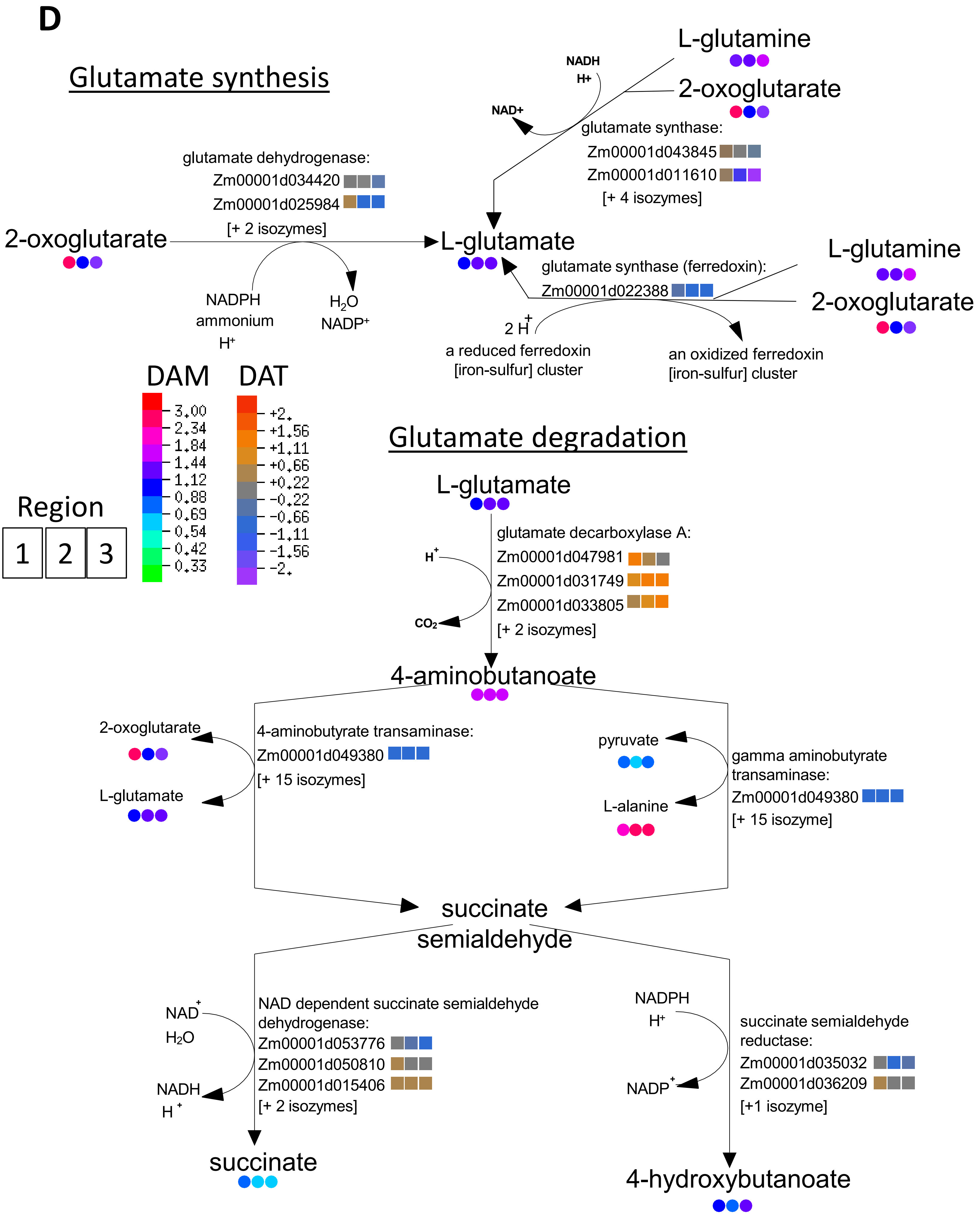

Supplementary Figure S14.

E

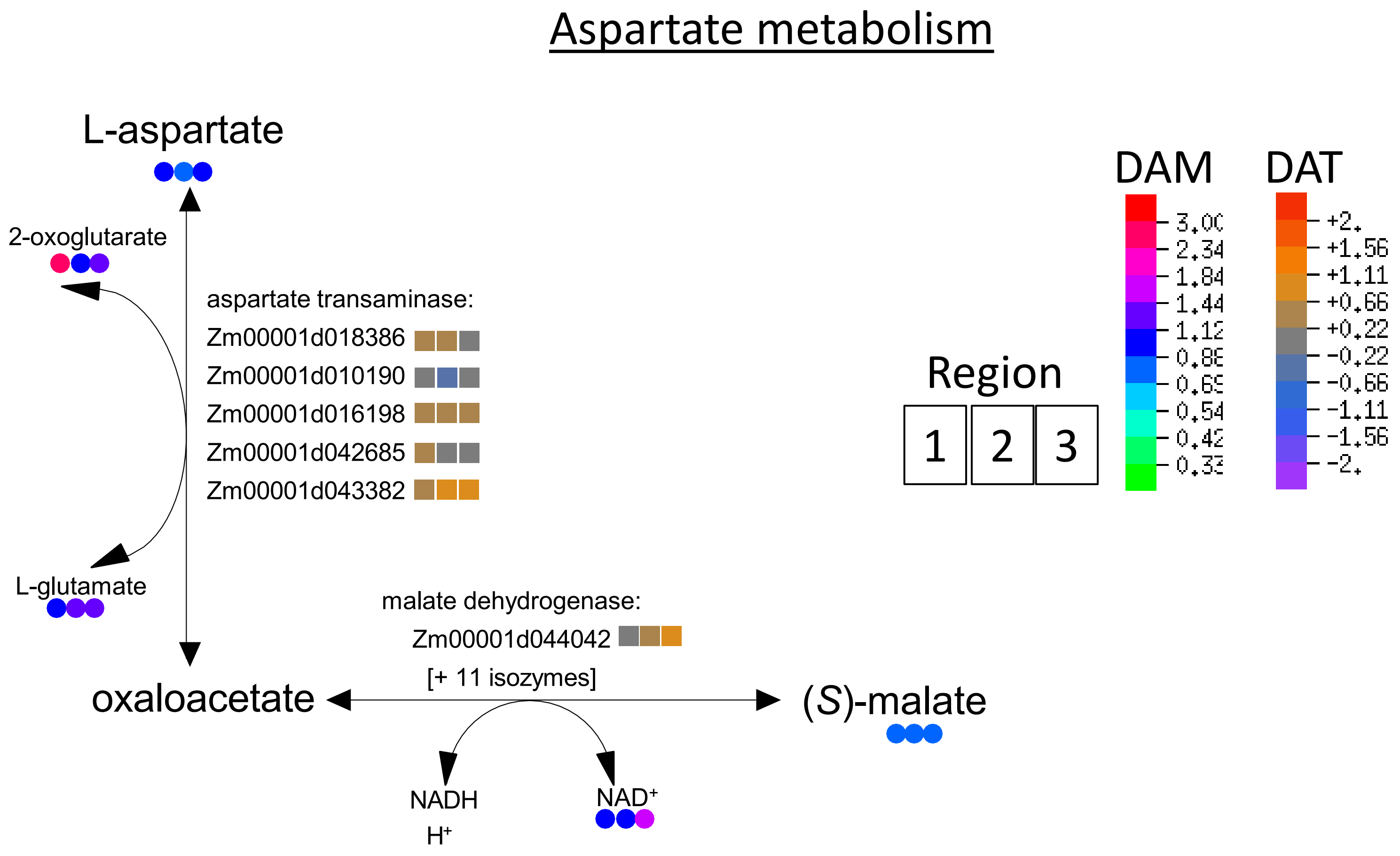

Supplementary Figure S14.

Supplementary Figure S14. Amino acid metabolic pathway analysis by integrated metabolomic and transcriptomic abundance changes in R1, R2 and R3 of the N2 root growth zone in response to WD. A) Arginine synthesis and degradation; B) alanine synthesis and degradation; C) valine, leucine and isoleucine metabolism; D) glutamate synthesis and degradation; E) aspartate metabolism. Colored circles indicate metabolite fold-change (value >1 indicates enrichment in WD compared to WW); colored squares indicate log_2 fold-change between transcript abundances of WD and WW (positive value indicates enrichment in WD relative to WW) in R1, R2 and R3 (left to right, respectively).

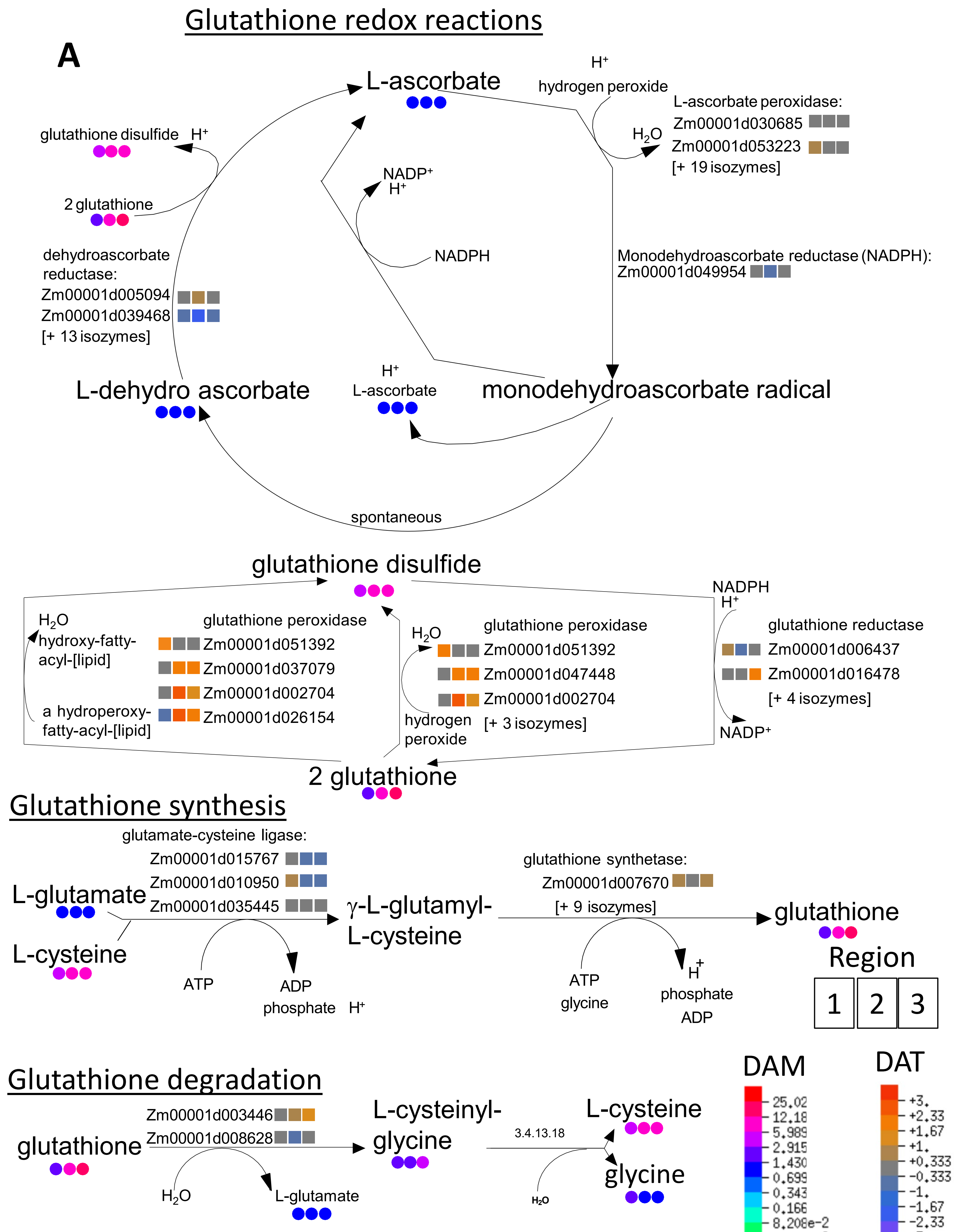

Supplementary Figure S15.

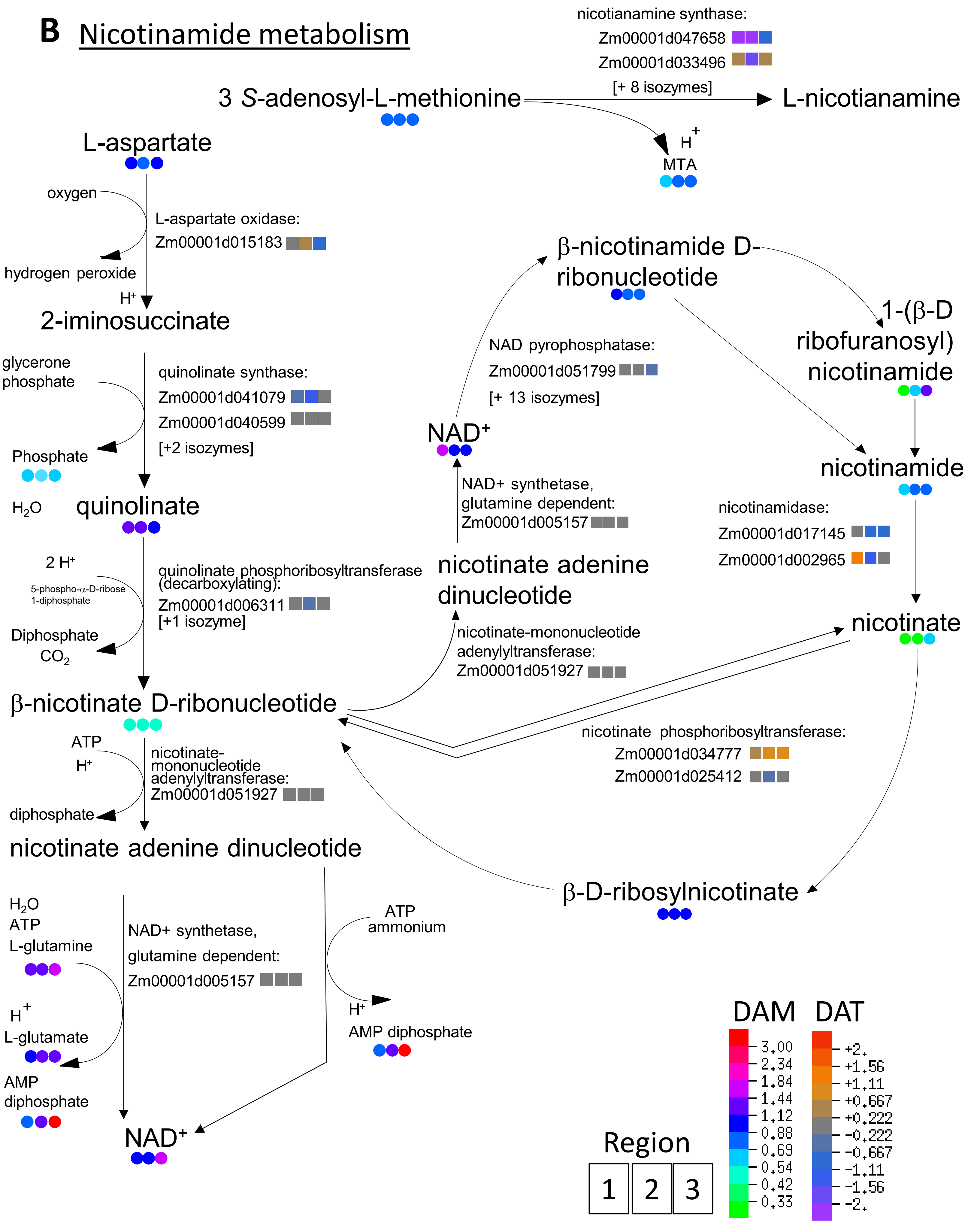

Supplementary Figure S15.

Supplementary Figure S15. Integration of metabolomic and transcriptomic abundance changes in response to WD reveals ROS homeostasis strategies in the N2 root growth zone. A) Glutathione synthesis, degradation, and redox reactions; B) nicotinamide metabolism. Colored circles indicate metabolite fold-change (value >1 indicates enrichment in WD compared to WW); colored squares indicate log_2 fold-change between transcript abundances of WD and WW (positive value indicates enrichment in WD relative to WW) in R1, R2 and R3 (left to right, respectively).

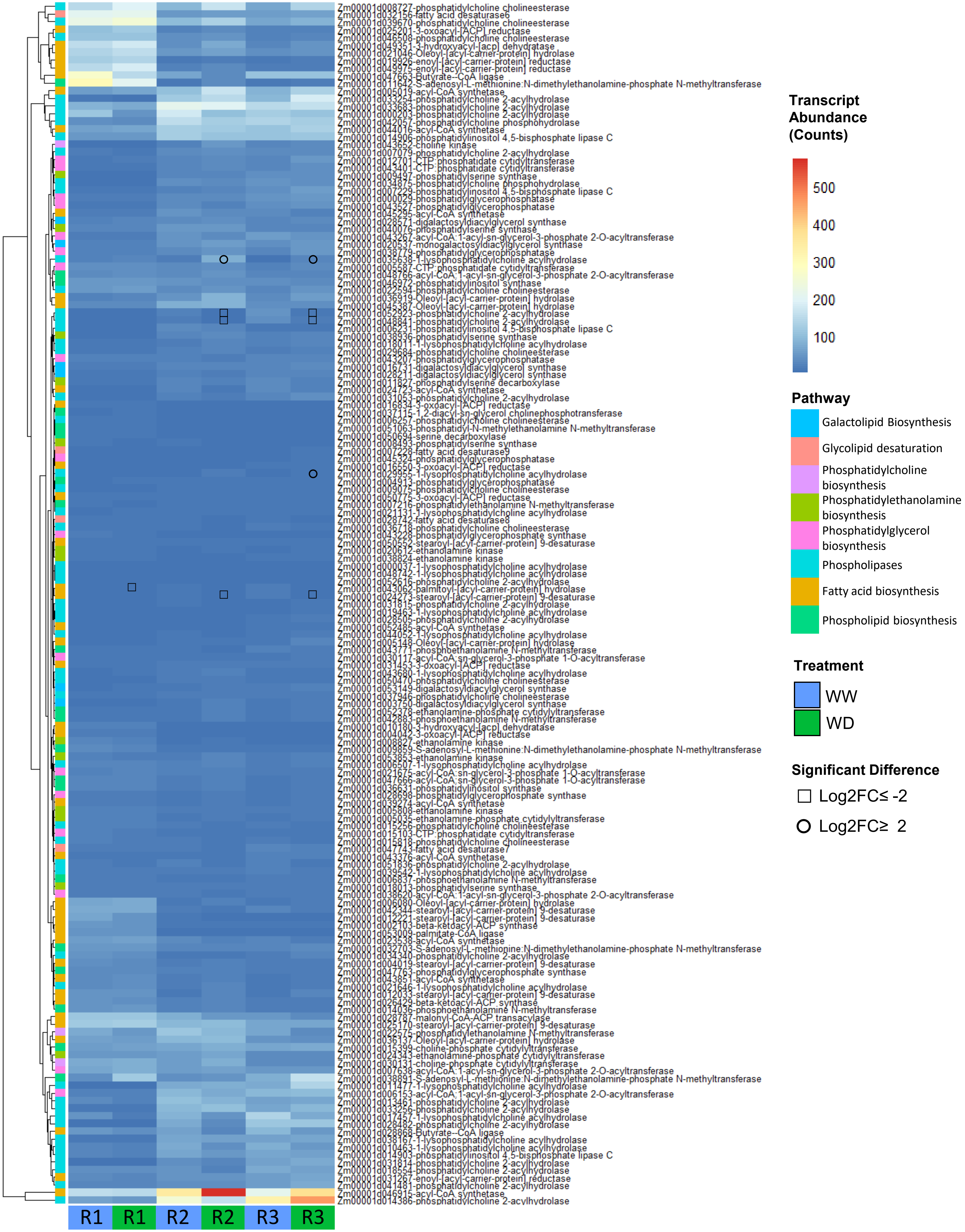

Supplementary Figure S16.

Supplementary Figure S16. Transcript abundances of phospholipid and glycerophospholipid metabolism related genes in R1, R2 and R3 of the N2 root growth zone in response to WD. Colored rectangles denote mean normalized transcript abundances (counts). Squares and circles in heatmap cells indicate significantly different transcript abundance (log2 FC $\leq$ -2 or $\geq$ 2, respectively) between treatments. Root regions indicated at bottom of heatmap.
